# Dual-constraint spatial planning unlocks proactive blue carbon expansion across China's coastlines

**DOI:** 10.1016/j.ese.2026.100771

**Published:** 2026-09-26

**Authors:** Yang Xu, Tianci Gu, Feng Xu, Lu Xing, Guangqing Chi, Ruibo Qiu, Chunyang He, Yangfan Li, Qingxu Huang

**Affiliations:** a State Key Laboratory of Earth Surface Processes and Disaster Risk Reduction, Beijing Normal University, Beijing, 100875, China; b School of Natural Resources, Faculty of Geographical Science, Beijing Normal University, Beijing, 100875, China; c Department of Land Resource Management, China University of Geosciences, Wuhan, 430074, China; d Department of Geography, Indiana University, Indiana, 47405, the United States; e Academy of Disaster Reduction and Emergency Management, Ministry of Emergency Management and Ministry of Education, Beijing, 100875, China; f State Key Laboratory of Marine Environmental Science, Key Laboratory of Coastal and Wetland Ecosystems (Ministry of Education), College of the Environment and Ecology, Xiamen University, Xiamen, 361005, China

**Keywords:** Proactive blue carbon, Dual-constraint spatial planning, Coastal transition zones, Land-sea anthropogenic pressures, Systematic conservation planning

## Abstract

Coastal blue carbon ecosystems—mangroves, salt marshes, and seagrasses—provide vital climate mitigation benefits, placing them at the forefront of the global 30×30 conservation agenda. Maximizing these benefits across complex coastal transition zones requires spatial frameworks that reconcile intrinsic ecological suitability with human threats. However, existing conservation assessments in intertidal habitats remain largely reactive, fragmented, and blind to cumulative land-sea stressors and future expansion potential. Here we show that a dual-constraint spatial planning framework coupling habitat suitability with land-sea anthropogenic pressures across China's 18,000-km coastline unlocks a proactive blue carbon expansion capacity 7 to 24 times larger than current extents. Integrating 60 environmental variables and 13 anthropogenic pressure indicators reveals distinct latitudinal shifts driven by temperature-salinity dynamics alongside sharp land-sea pressure gradients in industrial bays. Systematic spatial optimization demonstrates that existing nature reserves cover only approximately 20% of high-priority zones, leaving over 70% of target areas dependent on active restoration rather than passive preservation. Our typology-based grid-governance framework bridges science and policy, providing a scalable model for coastal nations to execute proactive blue carbon management across threatened intertidal environments.

## Highlights

• 1. Dual-constraint spatial planning reconciles habitat suitability with land-sea pressures across China's coastlines.
• 2. Suitability modeling reveals proactive blue carbon expansion potential 7–24 times larger than current extents.
• 3. Existing nature reserves cover only ∼20% of priority zones, leaving >70% needing active restoration.
• 4. Grid-based spatial governance provides a scalable model to achieve global 30×30 conservation targets.

## Graphical abstract

**Figure:**
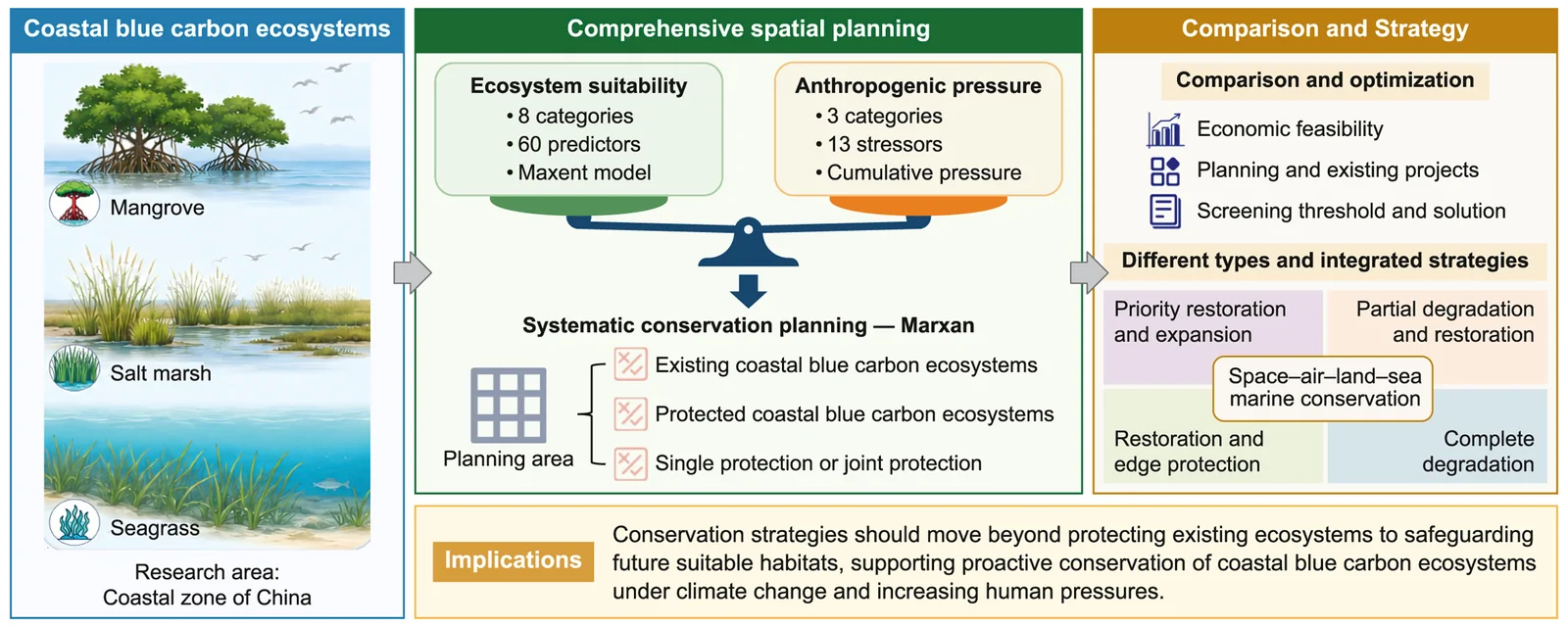

## 1 Introduction

Coastal blue carbon ecosystems (CBCEs) comprise mangrove, salt marsh, and seagrass ecosystems, which are complex socioecological systems comprising vegetation communities and associated soils, microorganisms, fauna, hydrological processes, and biogeochemical functions [[1], [2], [3]]. Similar to terrestrial ecosystems, coastal ecosystems can store substantial amounts of carbon compounds. Studies have shown that, compared with terrestrial systems, coastal habitats can have greater carbon sequestration capacity, higher carbon accumulation rates, and longer-term carbon storage, highlighting their role in climate change mitigation and adaptation [[4], [5], [6], [7], [8]]. Beyond climate regulation, CBCEs provide critical ecosystem services, such as shoreline stabilization, sediment trapping, and storm surge attenuation, and can also support recreational activities [8,9]. Because CBCEs are located primarily in intertidal zones at the land–sea interface, they face multiple stressors from climate change, anthropogenic activities, and marine influences [10]. Research has highlighted CBCE vulnerability to environmental changes, including sea-level rise [11], species invasion and competition [12,13], eutrophication [14], hydrological changes [15], land-use changes [16], and mariculture expansion [17]. Over the past two decades, substantial losses of mangroves [18], salt marshes [19], and seagrass areas [20] have reduced carbon sinks and increased coastal vulnerability [3,7,21]. Despite the increasing awareness of their socioecological value and ongoing conservation and restoration initiatives in regions [22,23] such as China [24,25] and North America [19,26], CBCE degradation and loss continue [16]. Given the essential services provided by CBCEs and the escalating pressures from anthropogenic activities and climate change, a full assessment of how environmental changes and anthropogenic activities affect these systems is urgently needed. Such an assessment is critical for proposing effective conservation strategies that increase coastal resilience and sustain vital functions [27].

The Kunming–Montreal Global Biodiversity Framework sets an ambitious target to effectively conserve at least 30% of the world's terrestrial and marine areas by 2030 [28]. Global consensus recognizes the urgent need to expand protected area networks while preventing ecosystem degradation. However, significant disagreements persist over where and how much to protect [29]. Existing conservation practices often fail to cover many high-priority regions [30], partly because they must reconcile two fundamental philosophies: proactive and reactive protection [31]. Proactive protection must conform to ecosystem suitability, which determines what ecosystems can be established, survive, and thrive under environmental conditions, thus defining the biophysical scope for conservation and restoration. Conversely, reactive protection conforms to anthropogenic pressure, reflecting the intensity and spatial variation in anthropogenic impacts. These impacts threaten ecosystem integrity, drive habitat loss, and constrain management feasibility.

Ecosystem suitability and anthropogenic pressure are prioritized in the conservation and management of CBCEs because they jointly determine the feasibility and sustainability of conservation measures. Suitability ensures efforts are ecologically viable, while pressure ensures they are socially and practically urgent. Neglecting either risks preserving unviable areas or failing to protect ecosystems under immediate threat, undermining the effectiveness and resilience of the entire conservation network. Long-term conservation strategies have largely focused on terrestrial green carbon ecosystems [32]. However, scientific understanding and practical conservation strategies for CBCEs remain limited, and a research gap persists in assessing ecosystem suitability and anthropogenic pressure for CBCEs [9,33]. Recent assessments of ecosystem suitability have largely integrated broad environmental variables, such as climate, topography, and soil properties, while neglecting critical biotic interactions and fine-scale abiotic drivers [[34], [35], [36], [37]]. These limitations have led to misjudgments of ecosystem suitability, causing numerous restoration projects to fail [38]. Moreover, quantifying anthropogenic pressure across coastal transition zones remains a major challenge because integrating land- and sea-based stressors is complex. Restored ecosystems are often difficult to sustain because of unpredictable and persistent anthropogenic disturbances [10,39]. A more critical issue is the methodological gap when identifying conservation priorities. Although various frameworks exist, such as two-dimensional overlay [40], gap analysis [41], multicriteria decision analysis [42], and systematic conservation planning (SCP) [43], these approaches remain insufficient for comprehensively addressing the dual constraints of ecosystem suitability and anthropogenic pressure and for responding to multi-ecosystem conservation needs [[44], [45], [46]]. Consequently, a more specific and robust methodological system that identifies CBCE protection priority areas must be developed. Although some innovative approaches have addressed the limitations of terrestrial systems, the research framework cannot be transferred to marine and coastal contexts [47,48]. This oversight results in protection plans that are often subjective (e.g., the strategies either overestimate restorability in highly stressed areas or underestimate the opportunities in suitable yet underexplored areas), lack regional comparability [[49], [50], [51]], and neglect connectivity within protected area networks [52].

In response, global conservation efforts have shifted from scientific advocacy to capacity building. The launch of the International Blue Carbon Initiative in 2011 significantly advanced scientific understanding of CBCEs and promoted their protection, restoration, and sustainable management within national policy frameworks, such as nationally determined contributions and greenhouse gas inventories. The establishment of the Chinese Regional Hub for Blue Carbon under the United Nations Decade of Ocean in 2025 strengthens the implementation of nature-based solutions and marine sustainability goals. As one of the few countries globally that host all three types of CBCEs, China possesses extensive CBCEs with high carbon sequestration potential [53]. However, anthropogenic activities heavily affect these ecosystems, making China a critical region for global CBCE conservation and restoration [54,55]. Existing research remains limited to local areas or single ecosystem types. The fragmented findings are inadequate for guiding the strategic layout of nationwide protection networks [56,57]. Therefore, integrating ecosystem suitability and anthropogenic pressure into a generalizable framework for identifying national-scale CBCE conservation priorities remains a pressing scientific challenge.

This study aims to quantify how ecosystem suitability and anthropogenic pressures jointly shape CBCE distribution and conservation priorities. Using China's coastal zone as a case study, encompassing mangrove, salt marsh, and seagrass ecosystems, we develop an integrated framework that combines potential ecosystem suitability, anthropogenic pressure assessment, and SCP. By integrating multiple environmental drivers and anthropogenic stressors, we identify spatial mismatches between ecosystem potential and current pressures, developed differentiated and grid-based management strategies, and delineate near- and long-term conservation priorities. This framework provides a basis for more targeted protection and restoration strategies for CBCEs (The conceptual diagram of the adopted methodological approach is shown in Supplementary Fig. S1).

## 2 Materials and methods

### 2.1 Distribution and protection dataset for CBCEs

We selected China's coastal zone as the study area, covering all regions along the Chinese coast that contain CBCEs, and extended the scope to inland seas, and territorial waters up to 12 nautical miles offshore, representing areas that could theoretically sustain CBCEs (Fig. 1). We compiled spatial distribution datasets for the three CBCEs: mangroves, salt marshes, and seagrasses. Mangroves and salt marshes for 2020 (the most recent year available) were obtained from the Geographic Data Sharing Infrastructure (www.gis5g.com), and seagrass distributions were obtained from the National Earth System Science Data Center (http://www.geodata.cn). The seagrass dataset was developed from field surveys, including Global Positioning System point collection along meadow boundaries and/or sonar scanning to accurately delineate seagrass patches and identify species composition between 2015 and 2020. It includes 127 georeferenced field survey records (Supplementary Table S2).

**Fig. 1:**
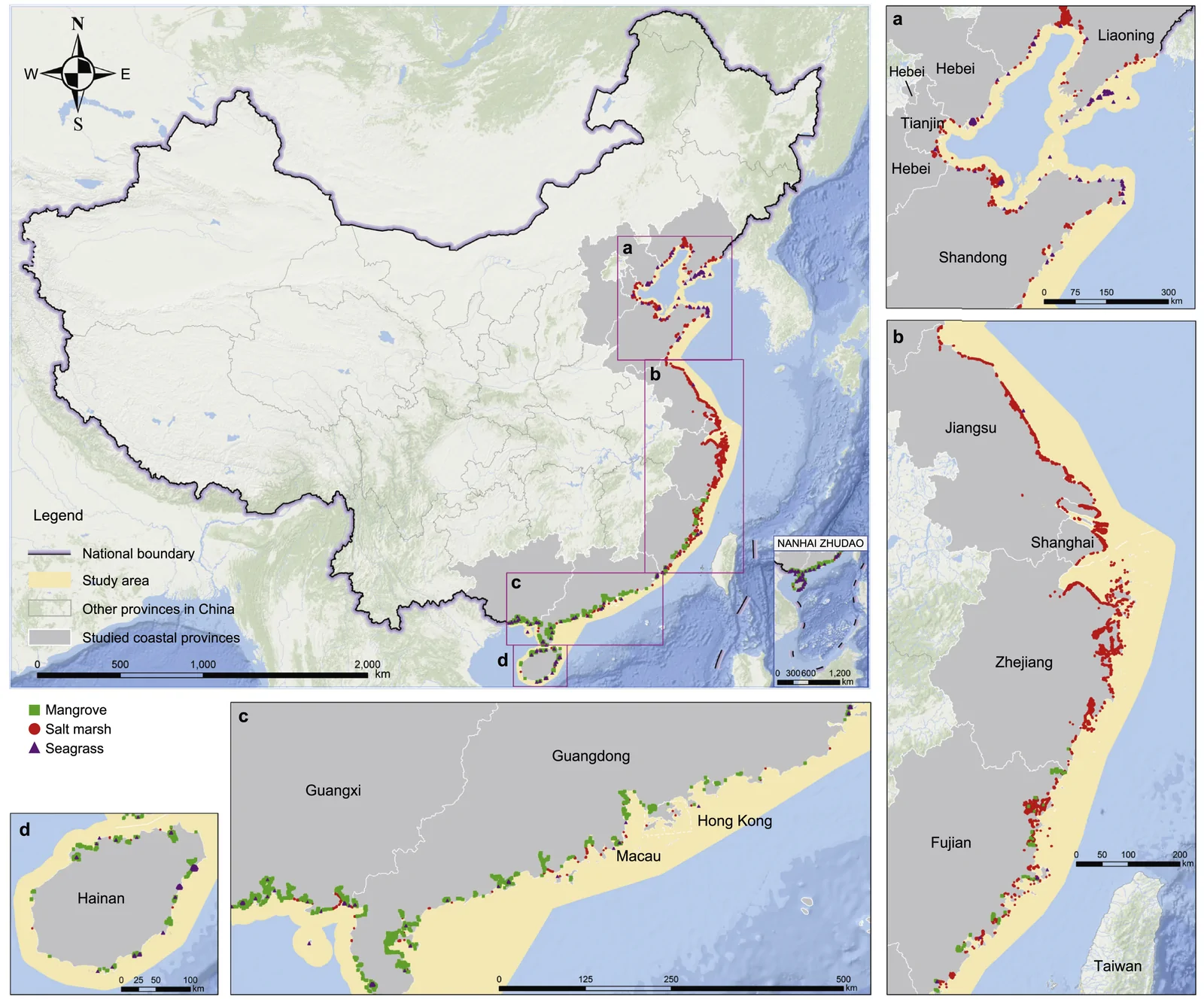
Distribution of coastal blue carbon ecosystems along the coast of China. The main map shows the study area and the distributions of mangroves, salt marshes and seagrasses. **a**–**d**, Enlarged views of the Bohai Rim (**a**), the Jiangsu–Shanghai–Zhejiang–Fujian coast (**b**), the Guangxi–Guangdong coast (**c**) and Hainan (**d**). Green squares, red circles, and purple triangles indicate mangroves, salt marshes, and seagrasses, respectively. Coastal provinces included in this study are shaded grey; Data were unavailable for Taiwan and islands in the South China Sea.

To resolve classification uncertainties in mixed and transitional zones, we supplemented these data with maps generated by Multiclass Blue Carbon Ecosystem Mapping based on Tide-level, Phenological, and Biophysical features derived from Sentinel-1 and Sentinel-2 imagery [58]. We resolved areas with conflicting classifications by visually interpreting high-resolution satellite imagery to determine the final ecosystem type. The final mapped extents covered 315 km^2^ of mangroves, 1745 km^2^ of salt marshes, and 267 km^2^ of seagrass areas.

Nature reserve data were downloaded from Zenodo (https://zenodo.org/records/14875797). In this study, we selected reserves where CBCEs are key conservation targets, thereby ensuring that existing protection efforts are considered in the subsequent prioritization (Supplementary Table S3).

### 2.2 Environmental predictors dataset and anthropogenic pressure dataset

To identify potential suitable distribution areas (PSDAs) rather than explain year-specific ecosystem distributions, we selected environmental predictors to represent the long-term ecological conditions governing CBCE occurrence. CBCEs are shaped by combined terrestrial and marine effects. To capture this duality, we compiled 60 environmental predictors across eight categories: (1) bioclimate (temperature and precipitation parameters), which defines the physiological tolerance of species [[59], [60], [61], [62]]; (2) soil, which serves as a foundational substrate affecting species distribution and function [63]; (3) offshore environment, which is critical for seagrass survival and expansion [64,65]; (4) weather [[66], [67], [68]]; (5) terrain, specifically elevation and slope, which are used as proxies for the inundation frequency and geomorphic setting, respectively [69,70]; (6) sea surface temperature, a key regulator of physiological processes [[71], [72], [73]]; (7) sea surface salinity, a major driver of ecosystem structure and function [74,75]; and (8) biotic interactions, representing inter-ecosystem facilitative or competitive effects [76].

CBCE distributions are primarily constrained by long-term environmental filtering and species physiological tolerances, making long-term climate normals more appropriate than short-term climatic observations for ecological niche modeling. Accordingly, climatic variables from WorldClim represented long-term baseline conditions, while terrain and soil variables were treated as relatively stable environmental attributes [77,78]. We derived marine environmental variables from multi-year datasets to characterize representative oceanographic conditions. Although the observation periods of different predictors are not identical, we selected all variables to describe broad-scale environmental suitability rather than short-term environmental fluctuations, following common practice in species distribution modeling studies [79,80].

While distribution modeling typically considers abiotic factors, biotic interactions are often overlooked despite their documented significance. These interactions can be facilitative or competitive, with their direction and strength often context-dependent [[81], [82], [83], [84]]. For example, salt marsh vegetation promotes mangrove seedlings at high latitudes, whereas the effect becomes inhibitory at low latitudes [85]. To quantitatively incorporate this mechanism, we adopted Cui et al.’s (2024) [86] biotic interaction index, calculated based on the proportion of the species distribution area in each grid, to characterize the pairwise interaction strength among CBCEs. Supplementary Table S4 provides detailed sources and descriptions of all variables.

Direct anthropogenic impacts, such as mechanical damage, eutrophication, mariculture, siltation, and coastal construction, drive widespread CBCE loss [87]. Following the pressure-source classification framework proposed by Halpern et al. [88,89], which includes fishing, climate change, ocean, and land-based factors, and excluding climate-related pressures already represented by the environmental predictors, we compiled an anthropogenic pressure source dataset comprising three major categories: land-based, ocean-based, and fishing-related pressures, with a total of 13 subcategories (see Supplementary Table S5 for the data sources, descriptions, and processing). Stressors vary by impact pathway. Land-based stressors include indicators such as light pollution [90], population density [91], and port activities [92], which directly affect ecosystem habitat quality. The main sources of ocean pressure are shipping [93] and mariculture [94,95]. Shipping can be further divided into bulk, cruise, and container types based on ship type. Fishing includes two modes, commercial and artisanal, which pose significant threats to marine ecosystems because of declining fish resources, waste discharge, and the accumulation of floating debris [96]. Considering that different types of fishing equipment can influence marine ecosystems to varying degrees [97,98], we classified commercial fishing equipment into five categories based on three factors (Supplementary Table S6).

We resampled all variables to a uniform spatial resolution of 30 arcsec and aligned them to a common grid extent and projected coordinate system to ensure spatial consistency in subsequent analysis. Because CBCEs are distributed mainly in the nearshore zone, we extended data coverage to the coastline to avoid inaccurate results due to spatial mismatch [99]. In most cases, gaps in the coastal shoreline data must be eliminated. In addition, most pressure sources exhibit a highly skewed normal distribution, with extremely high values compressing the numerical range of low-to medium-value data after standardization, thereby underestimating their relative impact strength. To address this, we log-transformed the pressure source values before rescaling and re-standardized the pressure source data to a 0–1 range, yielding a unified dimensionless scale for direct comparison.

### 2.3 Estimating PSDAs for CBCEs

We used the maximum entropy (MaxEnt) model (version 3.4.4) to identify the PSDAs. The MaxEnt model has shown strong performance in modeling species distributions and clarifying relationships between species and environmental variables [100,101]. The model estimates the probability distribution of ecosystem suitability by maximizing entropy subject to constraints defined by observed occurrence data and environmental predictors [102].

Occurrence records for each ecosystem were randomly divided into training (75%) and testing (25%) datasets. ‌We used the receiver operating characteristic‌ curve to evaluate MaxEnt performance, plotting the false positive rate (*x* axis) against the true positive rate (*y* axis). The area under the curve (AUC) value indicates model accuracy, with a larger value indicating higher accuracy. Variable importance was assessed using percent contribution (i.e., the increase in regularized gain during model training) and permutation importance (i.e., the decrease in the AUC value when the variable values are permuted). We analyzed the variables' impact on the distribution by creating response curves.

Models were run using 10 replicates with random resampling. Other parameters were set to default values, including a convergence threshold of 10^−5^, a maximum of 500 iterations, and 10,000 background points. In this study, ecosystem suitability refers to the probability of occurrence predicted by the MaxEnt model for each CBCE type under a given set of environmental conditions. We then delineated PSDAs by applying a suitability threshold to the continuous suitability outputs. Following common practice, we classified areas with predicted probabilities ≥0.6 as PSDAs, ≥0.8 as highly PSDAs, and <0.5 as unsuitable areas [[103], [104], [105]].

To reduce multicollinearity and model overfitting, we considered environmental variables with a Pearson correlation coefficient |*r*| ≥0.8 highly collinear and retained only one from each correlated pair for initial modeling (Supplementary Fig. S2). We then ran an initial MaxEnt model incorporating all noncollinear variables relevant to each ecosystem type to estimate each variable's contribution. Final predictor sets for each ecosystem were selected by integrating the correlation analysis results, contribution scores, and known ecological drivers of CBCE distribution (Supplementary Table S4).

### 2.4 Assessing the effect of anthropogenic pressure on CBCEs

While assessments of anthropogenic footprints on land are well established, the cumulative effects of anthropogenic activities in coastal transition zones remain poorly quantified [106]. To address this gap, we drew on Halpern et al.’s [89] ecosystem-specific impact assessment framework, which integrates multiple anthropogenic stressors by weighting their relative impacts across ecosystem types. The cumulative anthropogenic pressure ($A_{q}$) for CBCE type *q* was calculated as:

(1) $$A_{q}=\sum_{p=1}^{k}D_{p}\times \mu_{p,q}$$

where *p* denotes a specific anthropogenic stressor from the set of *k* stressors, *D*_*p*_ is the standardized intensity of stressor *p* (0–1), and *μ*_*p*,*q*_ is a weighting factor representing the relative vulnerability of CBCE type *q* to stressor *p* (see Supplementary Table S7 for the expert-derived weight matrix). These weights, derived from structured expert surveys, reflect the expected relative impact per unit pressure when a stressor co-occurs with an ecosystem in a given grid cell, not its global-scale importance.

### 2.5 Determination of the priority order of protection and comprehensive protection strategies

***Theoretical basis and tool selection*.** SCP theory provides a quantitative framework for identifying priority areas for biodiversity conservation and has become a mainstream paradigm in conservation practice by comprehensively evaluating conservation effectiveness, spatial connectivity, and socioeconomic costs [[107], [108], [109]]. We used Marxan, a widely applied decision-support tool in SCP based on a simulated annealing algorithm. Marxan is designed to achieve predefined conservation targets at minimal cost while maximizing the spatial compactness and connectivity of the reserve network. Its rigorous, transparent, and flexible nature renders this tool particularly suitable for marine and coastal protected area designs [[110], [111], [112]]. The target function (*T*) can be expressed as:

(2) $$T=\sum_{i=1}^{n}x_{i}c_{i}+b\sum_{i=1}^{n}\sum_{h=1}^{n}x_{i}\left(1-x_{h}\right)v_{i,h}+\sum_{j=1}^{m}F_{j}S_{j}$$

where *n* is the number of planning units (PUs), and *m* is the number of conservation features. The binary variable *x*_*i*_ indicates whether PU *i* is included in the conservation network (*x*_*i*_ = 1) or excluded (*x*_*i*_ = 0), and *c*_*i*_ denotes the cost of PU *i*. *b* is the boundary length modifier (BLM), a parameter that determines the degree of aggregation of the protected area system; *v*_*i,h*_ reflects the cost of the connection between PUs *i* and *h*, typically measured as the shared boundary between these two PUs. *F*_*j*_ represents the penalty factor of feature *j*, a scaling factor that determines the relative importance of meeting the representation target; and *S*_*j*_ represents the penalty score of feature *j*.

***Parameterization and scenario design*.** The study area was divided into 397,961 PUs. PU status was defined using two categories: (1) PUs currently within a protected area and containing existing CBCEs (locked-in; PU status = 2), which were mandated to remain in the final solutions, and (2) all other PUs (PU status = 0), which could be either selected or not. In total, 359 mangroves, 1320 salt marshes, and 63 seagrass PUs were locked in.

We developed a parameterized conservation prioritization framework based on three core SCP criteria: importance, vulnerability, and feasibility [113]. Importance reflects the conservation value of natural assets, following the principles of comprehensiveness, representativeness, and irreplaceability, and forms the basis for determining the types and areas to be protected, focusing on biodiversity factors such as species abundance and habitat status [114,115]. In this study, we used the potential distribution probability of CBCEs as a quantitative indicator of the number of protection features. Vulnerability reflects ecosystem sensitivity to anthropogenic interference [116,117], which was incorporated as a cost parameter. Feasibility reflects the implementation cost and efficiency of protective actions, considering multiple practical options [118]. Therefore, we constructed nine scenario models by setting protected area targets ranging from 10% to 90% (10% increments) to simulate protection effectiveness under different policy commitments and funding levels. In addition, because the PSDAs and anthropogenic pressures differ across the three CBCE types, Marxan optimization was performed separately for mangroves, salt marshes and seagrasses.

***Scheme comparison and selection*.** The optimal BLM value depends on the cost and geometric characteristics of a given PU. From low to high, solutions range from less expensive, fragmented solutions to more compact, expensive solutions. In this study, we used the ArcMarx Toolbox to estimate the optimal BLM value (Supplementary Table S8). Moreover, we selected PUs with potential distribution probabilities greater than 0.1, 0.2, 0.3, 0.4, and 0.5 for the different CBCEs as new research areas to improve protection efficiency and reduce planning costs while balancing protection goals and effectiveness. By comparing different probability screening methods with unfiltered schemes, we identified the most suitable screening probability. For each combination of conservation scenario and threshold, Marxan was run for 100 iterations. Selection frequency across iterations (0–100) was used to evaluate the relative importance of PUs. Solutions with frequency ranges of 80–100 (R1), 60–100 (R2), 40–100 (R3), and 20–100 (R4) were considered frequency solutions under different protection importance levels. In addition, the Marxan model provided the lowest-cost solution among 100 iterations (R0). The final conservation scheme was selected by comparing these alternative solutions.

***Final conservation scheme design*.** We divided the final PUs into five categories: mangrove protected areas, salt marsh protected areas, seagrass protected areas, priority areas for anthropogenic pressure management, and other areas (Supplementary Fig. S3a and b). We overlaid the three types of protected CBCE areas obtained with the Marxan model for a comprehensive design (Supplementary Fig. S3c). When a PU already contained one or more CBCEs, we divided them into two protection types based on whether these ecosystems are planned for protection: maintaining current protection and facing degradation risks. When a PU does not currently contain a CBCE but is planned for protection, we assigned a restoration protection strategy. PUs containing multiple CBCE types were assigned a joint protection strategy, whereas PUs containing a single CBCE type were assigned a single protection strategy. For joint protection PUs, the main protected ecosystem should first consider the existing CBCE, and then determine it by comparing potential distribution probabilities. For unprotected PUs without current CBCEs, priority areas for anthropogenic pressure management were identified according to anthropogenic pressure intensity. PUs with relatively low anthropogenic pressure were classified as other areas. Finally, all PUs were classified and visualized using different colors to generate a grid-based differentiated governance strategy (Supplementary Fig. S3d). Although uncertainty remains about the final strategy under the protection target, higher protection targets generally provide greater protection effectiveness. Therefore, the protection strategy allows flexibility to adjust protection targets in the future (Supplementary Fig. S3e).

## 3 Results and discussion

### 3.1 PSDA pattern and environmental drivers of CBCE suitability

The MaxEnt simulation results revealed substantial spatial expansion potential for all CBCEs. The PSDAs (occurrence probability >0.6) covered 4188.85 km^2^ for mangroves, 13,477.29 km^2^ for salt marshes, and 6255.88 km^2^ for seagrasses, representing approximately 13-, 7-, and 24-fold increases relative to their current extents, respectively (Supplementary Fig. S4). Among the three ecosystems, salt marshes exhibited the largest potential distribution area. Suitable CBCE types differed significantly among provinces, with a general latitudinal trend from mangrove dominance in the south to salt marsh dominance and then to seagrass dominance in the north (Supplementary Fig. S4d). The predicted PSDA patterns were consistent with previous independent habitat suitability assessments and were further supported by model validation and occurrence record sensitivity analyses (Supplementary Text S1; Supplementary Fig. S20–S21; Supplementary Table S16). In addition, the response mechanisms to environmental variables varied across CBCEs (Supplementary Fig. S5). Elevation was a key determinant of habitat suitability for all CBCEs, with the highest suitability occurring near sea level. As intertidal ecosystems, CBCEs rely on specific tidal inundation and hydrological conditions, which largely restrict their PSDAs to coastal zones.

Mangrove PSDAs were concentrated in southern coastal bays, accounting for nearly all PSDAs (Fig. 2a–c; Supplementary Fig. S4a). The suitability of mangroves decreased with increasing latitude, and mangroves were rarely found in areas north of 28° N. Temperature- and precipitation-related variables were the dominant predictors, indicating that warm and humid conditions favor mangrove establishment (Supplementary Fig. S5a). High water vapor pressure (WVP) and sea surface salinity increase the distribution probability of mangroves, indicating that suitable hydrothermal and salinity conditions are key to the survival of mangroves.

**Fig. 2:**
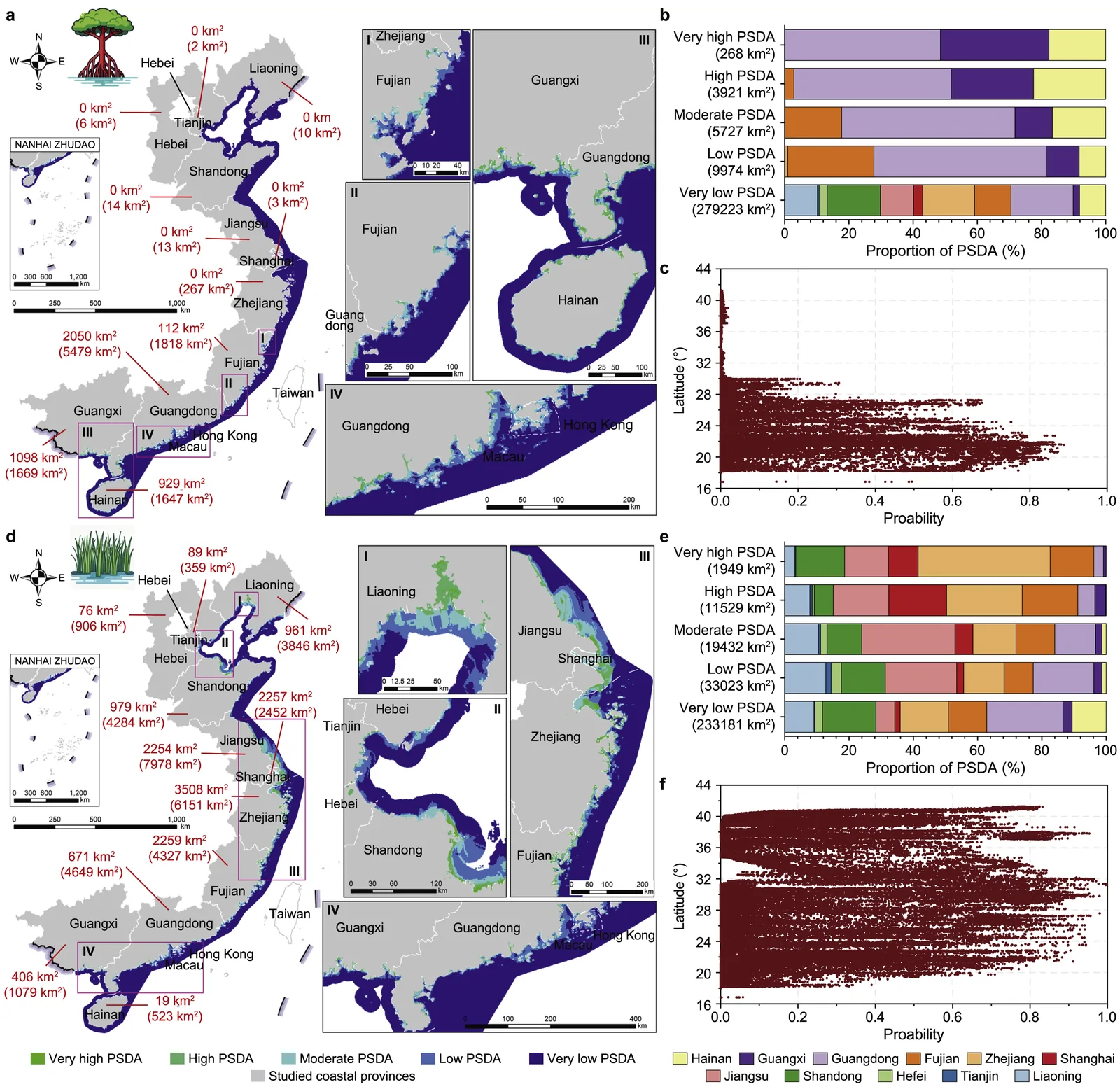

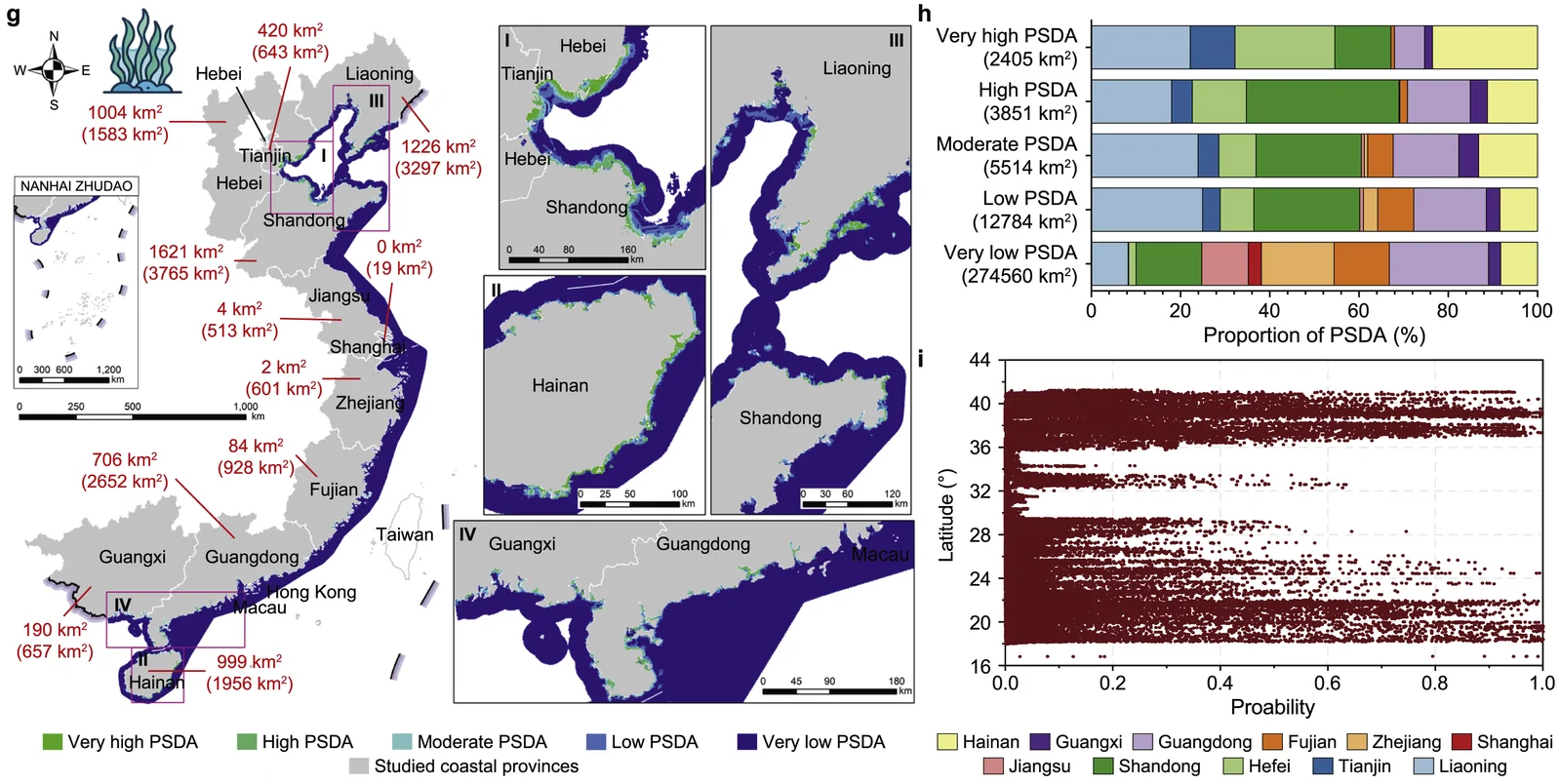
Potential suitable distribution areas for coastal blue carbon ecosystems. **a,d**,**g**, Spatial distributions of five PSDA classes for mangroves (**a**), salt marshes (**d**), and seagrasses (**g**). For each province, red annotations indicate the total area with a predicted occurrence probability greater than 0.6; values in parentheses denote the expected suitable area, calculated as the sum of predicted probability multiplied by planning unit area across all units. Insets enlarge representative coastal regions. **b**,**e**,**h**, Provincial contributions to each PSDA class for mangroves (**b**), salt marshes (**e**), and seagrasses (**h**). Numbers in parentheses indicate the total area within each class. **c**,**f**,**i**, Latitudinal distributions of predicted occurrence probabilities for mangroves (**c**), salt marshes (**f**), and seagrasses (**i**); each point represents a planning unit. PSDA, potential suitable distribution area. Coastal provinces included in this study are shaded grey; Data were unavailable for Taiwan and islands in the South China Sea.

Salt marsh PSDAs were concentrated along the eastern coast, showing an outward extension trend along the coast (Fig. 2d–f; Supplementary Fig. S4b). In addition, the bays along the Bohai Sea coast represented the main PSDAs for salt marshes in the north. Environmental factors indicate that distribution probability is highest when isothermality ranges from 20 to 25, corresponding to mid-latitude coastal regions in eastern China (Supplementary Fig. S5b). Low WVP and high sea surface salinity seasonality benefit salt marsh expansion, while soil physical and chemical properties, such as soil conductivity, coarse particle content, and exchangeable sodium percentage, also affect their distribution.

Seagrass PSDAs were concentrated mainly along the northern and southern coasts, with almost no PSDAs along the central coast (Fig. 2g–i; Supplementary Fig. S4c). High PSDAs for seagrass areas accounted for a larger proportion and were concentrated on several major peninsulas. Seagrass suitability showed a non-linear latitudinal pattern, decreasing initially and then increasing towards higher latitudes. Sea surface temperature exhibited a bimodal distribution, with peaks at 13–15 °C and 27–29 °C (Supplementary Fig. S5c). In addition, hydrological environmental factors such as chlorophyll content, suspended sediment concentration, sea surface salinity, and off-water radiation are key drivers of their distribution.

The environmental controls identified in this study provide insights into the major climatic threats facing CBCEs in China. As ecosystems located at the land–sea interface, CBCEs are simultaneously exposed to terrestrial and marine climatic influences. Temperature emerged as the dominant driver of ecosystem suitability, largely determining the latitudinal distribution of CBCEs, followed by salinity, which strongly constrains species establishment and persistence [34,40,86]. Beyond these common controls, mangroves were more sensitive to precipitation, salt marshes to soil conditions, and seagrasses to marine environmental properties. Consequently, climate warming, altered hydrological regimes, and changing marine environmental conditions may affect different CBCE types through distinct pathways, potentially reshaping their future distributions.

Biotic interactions exhibited marked asymmetry across habitats (Supplementary Fig. S5). The mangrove and seagrass interaction indices exhibited weak effects, whereas the salt marsh interaction index (SMII) strongly negatively affected mangrove suitability, leading to near-complete spatial exclusion (Supplementary Fig. S4d). Its effect on seagrasses was context-dependent. When the proportion of salt marshes was low, the effect was manifested mainly as competitive inhibition, and when the proportion increased, SMII yielded a promoting effect, which could be related to the improvement of habitats or the provision of nutrients by salt marshes. These effects diminished significantly under extreme salt marsh dominance. These interactions could help explain regional coexistence patterns in the north, salt marsh dominance at the center, and low co-occurrence in the south (Supplementary Fig. S4d).

### 3.2 Anthropogenic pressure on CBCEs

Anthropogenic pressure exhibited a clear land–sea gradient, which systematically decreased from the coast toward the open ocean, with the land–sea interface being the most significantly affected by anthropogenic activities (Fig. 3; Supplementary Fig. S6). Coastal regions of Shandong, eastern Tianjin, and southeastern Liaoning experienced higher anthropogenic pressure, characterized by large-scale mariculture and fishing activities. Coastal regions of Zhejiang, Fujian, and Guangdong also experienced high anthropogenic pressure, mainly because they lie along major shipping routes, where maritime traffic amplifies environmental stress on top of preexisting anthropogenic impacts. By contrast, anthropogenic pressure along the Jiangsu coast, particularly along its central coast, remained extremely low. This area has no large-scale mariculture or fishing operations, a sparse port distribution, and low shipping activity. This makes it a place with a stable marine environment and minimal anthropogenic disturbance. Across the three CBCE types, distribution patterns were largely similar, although seagrass areas experienced greater pressure. Overall, the coastal regions of Shandong and Guangdong were most intensely and extensively affected by anthropogenic pressure, while the coasts of Jiangsu, Shanghai, and Hainan experienced weaker impacts.

**Fig. 3:**
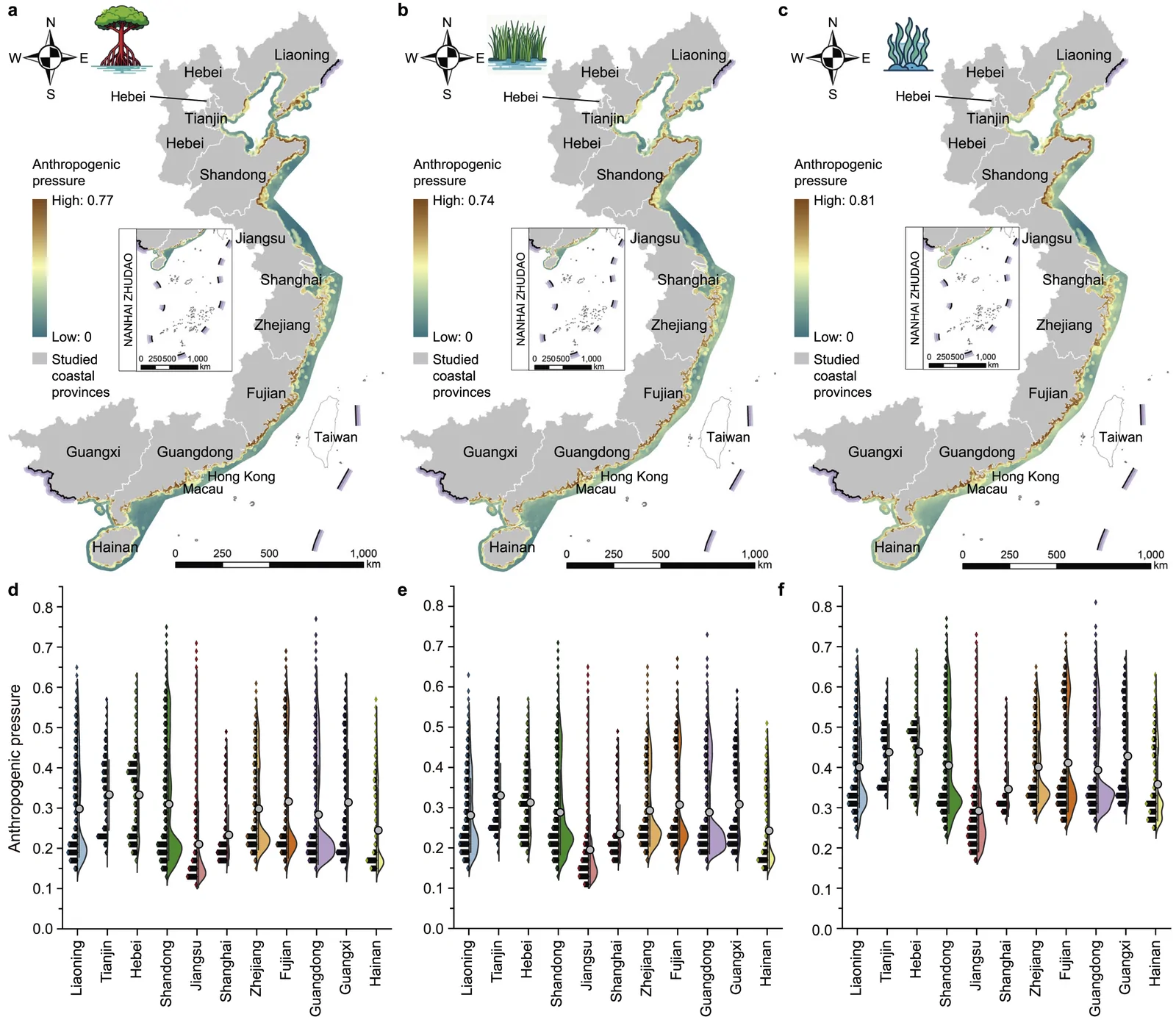
Anthropogenic pressure across coastal blue carbon ecosystems. **a–c**, Spatial distributions of anthropogenic pressure in mangrove (**a**), salt-marsh (**b**), and seagrass (**c**) areas. **d**–**f**, Corresponding distributions of the anthropogenic pressure index among coastal provinces in mangrove (**d**), salt marsh (**e**), and seagrass (**f**) areas. Violin widths represent the kernel density distribution of anthropogenic pressure values across planning units. Diamonds indicate the binned frequency distribution of observations, the vertical line represents the interquartile range (25–75th percentiles), and the grey circle indicates the mean. Coastal provinces included in this study are shaded grey; Data were unavailable for Taiwan and islands in the South China Sea.

The observed pressure patterns indicate that coastal urbanization and marine resource exploitation remain the most immediate anthropogenic threats to CBCEs in China. High-pressure hotspots are primarily associated with intensive aquaculture activities, commercial shipping routes, port development, and fishing operations, all of which contribute to habitat fragmentation, sediment disturbance, water quality deterioration, and ecological degradation [87,92,94]. The pronounced land–sea gradient further shows that CBCEs are simultaneously exposed to terrestrial and marine stressors, underscoring the need for integrated land–sea conservation strategies. These findings align with global anthropogenic impacts on marine areas reported by Halpern et al. [89], reinforcing the conclusion that anthropogenic activities exert spatially heterogeneous pressures on coastal ecosystems.

### 3.3 Comparison of solutions and the optimal selection

Through a comparative analysis of overall spatial patterns, local distributions, and various evaluation metrics (Supplementary Fig. S7–S17 and Table S9–S14), we obtained the optimal screening threshold for potential distribution probability and the preferred frequency solution. The results indicated that a higher screening threshold concentrated protection efforts in fewer, more ecologically viable PUs. Given the vast study region and limited conservation management resources, applying a threshold based on the potential ecosystem suitability probability of CBCEs can exclude areas unsuitable for growth, thereby optimizing conservation networks.

However, higher thresholds did not always improve conservation outcomes. Although increasing the screening threshold slightly increases anthropogenic pressure per unit area (with a minimal increase under high conservation targets), this increase is substantially outweighed by the increase in suitability probability. When the screening threshold exceeds 0.3, the average potential distribution probability per unit area surpasses 0.5 across all scenarios. Furthermore, a higher threshold corresponds to a significantly lower anthropogenic pressure per unit of distribution probability. However, a higher threshold is not invariably preferable. Excessively high thresholds can undermine ecological connectivity. Under low conservation targets, overreliance on the potential distribution probability may force the inclusion of areas with high anthropogenic pressure in the protection network, thereby disconnecting the planned layout from actual conservation needs. In addition, with a PU status of 2, these units nearly constitute the entire solution when the threshold is high and the conservation target is low. Based on a comprehensive comparison, a threshold for the potential distribution probability >0.4 was ultimately adopted.

The Marxan-derived optimal solution (R0) showed limited stability because it represents only the lowest-cost outcome among 100 runs and does not adequately prioritize the optimal potential distribution probability. Frequency-based solutions provide a more robust alternative by identifying PUs selected repeatedly across iterations. Among the frequency solutions, R1–R4 encompass progressively greater conservation extents. The expansion rate decreases under higher conservation targets but increases under lower ones. We observed significant differences in the average unit potential distribution probability and average anthropogenic pressure between the frequency solutions at thresholds of 0.0 and 0.1. However, these differences essentially disappeared when the threshold was increased to 0.2 or above. Spatial analysis showed that, compared with the R1 solution, the R2 solution substantially reduced instability in PU selection and enhanced protected area connectivity. Compared with R3 and R4, R2 offered greater spatial focus, helping identify conservation priorities. Moreover, the proportional curves of the various metrics for R2 most closely conformed to the diagonal, indicating its superior performance in achieving the predefined conservation target and accurately reflecting the intended conservation effort and effectiveness.

### 3.4 Spatial pattern of the comprehensive CBCE protection strategy

Using a potential distribution probability threshold of 0.4 and the R2 frequency solution, we developed a comprehensive CBCE conservation strategy under a 30% protection target consistent with the Kunming–Montreal Global Biodiversity Framework (Fig. 4; Supplementary Table S15). Salt marshes accounted for the largest proportion of planned protected areas (16,850.93 km^2^; >67%), followed by mangroves (4366.26 km^2^) and seagrasses (3833.83 km^2^). Notably, more than 70% of the planned protection strategies target areas where CBCEs are currently absent (17,745.03 km^2^, of which mangroves, salt marshes, and seagrass account for approximately 11%, 46.7%, and 13.5% of the total area, respectively). This highlights the urgent need to restore and expand CBCEs, focusing primarily on existing distributions and their surrounding areas. Conversely, existing but unprotected CBCEs are primarily concentrated on the landward side near the shore, which is largely associated with increased anthropogenic pressure and corresponding degradation risks.

**Fig. 4:**
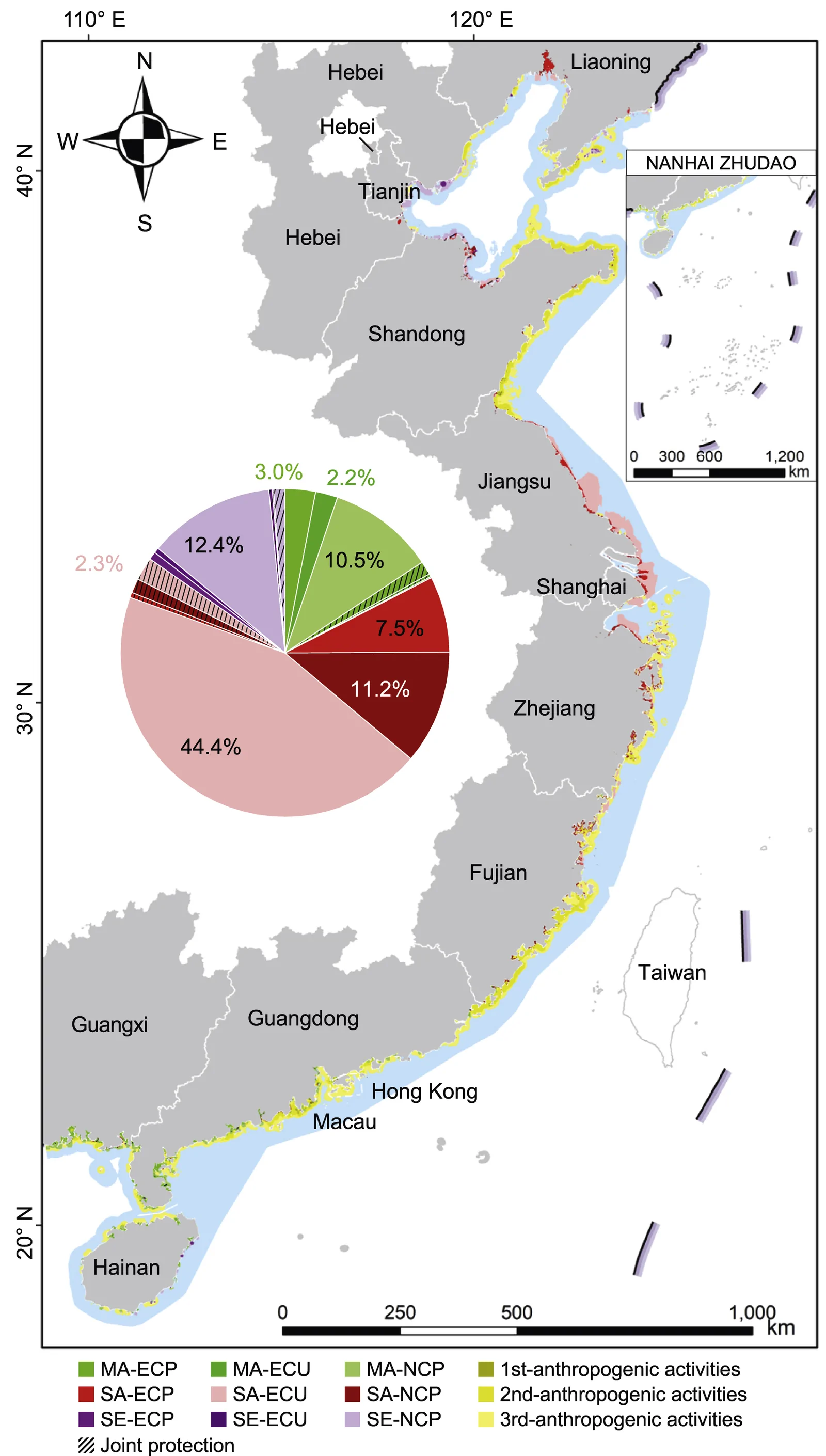
Spatially differentiated conservation and management strategies. Spatial allocation of protection and management strategies for mangrove, salt marsh, and seagrass ecosystems across the full study area. Colors distinguish ecosystem type, existing ecosystem status, protection status, and anthropogenic-use priority. Pie charts show the proportions of the strategy classes. Percentage labels are omitted for values < 2%. MA, mangrove; SA, salt marsh; SE, seagrass; CBCE, coastal blue carbon ecosystem; ECP, existing CBCEs requiring protection; ECU, existing CBCEs not requiring additional protection; NCP, areas without existing CBCEs but identified as conservation priorities. Hatched areas indicate joint protection zones. Joint protection zones are areas designated for the protection of two or three coastal blue carbon ecosystem types. Coastal provinces included in this study are shaded grey; Data were unavailable for Taiwan and islands in the South China Sea.

In regions with high anthropogenic pressure, even areas with high ecosystem suitability are mostly designated for anthropogenic activities or as non-protected zones. More than 90% of protected areas involve only a single CBCE type, which may reduce the implementation complexity but requires attention to potential ecological linkages and synergistic needs in jointly protected zones. Spatially, protection strategies shift from north to south, moving from seagrass dominance (with salt marshes as secondary CBCEs) to salt marsh dominance and then to mangrove dominance (with seagrass areas as secondary CBCEs) (Supplementary Fig. S4). This trend reflects the controlling influence of large-scale environmental gradients, such as temperature and salinity, on CBCE distribution and function, indicating that our conservation planning scheme follows natural eco-geographical principles.

Mangrove conservation and restoration focus primarily on Guangdong, followed by Guangxi and Hainan, which showed similar spatial planning patterns, whereas other coastal regions were less represented (Fig. 4; Supplementary Fig. S4a). More than half of the planned protected areas currently encompass mangrove distributions and require protective measures based on this status, which are concentrated mainly in southern Guangdong and the coastal regions of Guangxi. In addition, many bays face both restoration potential and degradation risk.

Salt marsh protection focuses on Jiangsu, followed by Zhejiang and Shanghai, while protection areas are also distributed in other provinces (Fig. 4; Supplementary Fig. S4b). Jiangsu encompasses extensive salt marsh restoration areas. Although many are not highly PSDAs, they are included in restoration plans because these coastal waters have relatively low anthropogenic disturbance and stable environmental conditions. The Yangtze River coast in Shanghai and more than half of the bays in Zhejiang and Fujian, face significant degradation risks. Similarly, the outer waters of Shanghai and Hangzhou Bay, and the border area between Zhejiang and Fujian, show considerable restoration potential. Bays in the northeastern and southwestern Bohai Sea face a dual governance model of degradation and restoration, while salt marshes in Tianjin, Guangdong, and Guangxi face nearly comprehensive degradation risks.

Seagrass protection focuses on the Bohai Sea region, including Liaoning, Tianjin, Hebei, and Shandong provinces (Fig. 4; Supplementary Fig. S4c). Although the South China Sea region contains many PSDAs, most are not designated as core seagrass protection zones. Seagrass protection strategies are largely restoration-oriented, primarily involving expansion around existing distributions. Furthermore, numerous zones coexist, and the transition between seagrass areas and salt marshes is often characterized by concurrent salt marsh degradation and seagrass restoration potential, necessitating integrated management.

In summary, the proposed spatial protection plan integrates ecosystem PSDAs, anthropogenic pressure, and restoration feasibility to identify ecosystems that require urgent protection and to proactively reveal key areas for future restoration and expansion. The identified priorities reflect differences in ecosystem suitability, connectivity to existing CBCEs, and strategic value in strengthening the ecological network's resilience, emphasizing a shift from passive preservation to active restoration and expansion. This approach provides a spatial basis for balancing protection and restoration objectives in CBCE management.

### 3.5 Bridging conservation planning with existing protected projects: aligning strategy with implementation

Nature reserves that primarily aim to conserve CBCEs play a critical role in maintaining their integrity and carbon sequestration function [119]. Our analysis revealed that existing nature reserves cover approximately 20% of the spatial extent identified as protected areas in this study, which is a clear indication that the existing protected area network is inadequate to meet conservation targets, particularly for CBCEs (Fig. 5). This gap highlights a systemic shortfall in the spatial allocation and functional design of existing conservation efforts. While the selected existing reserves align well with our identified priority zones, others fall outside areas of high ecosystem suitability or restoration potential, suggesting that historical reserve placement has not consistently followed systematic, spatially explicit ecological criteria. As climate change continues to reshape the future distribution of CBCEs, conservation strategies focused solely on current ecosystem locations may overlook highly suitable habitats that could support future expansion, migration, and restoration.

**Fig. 5:**
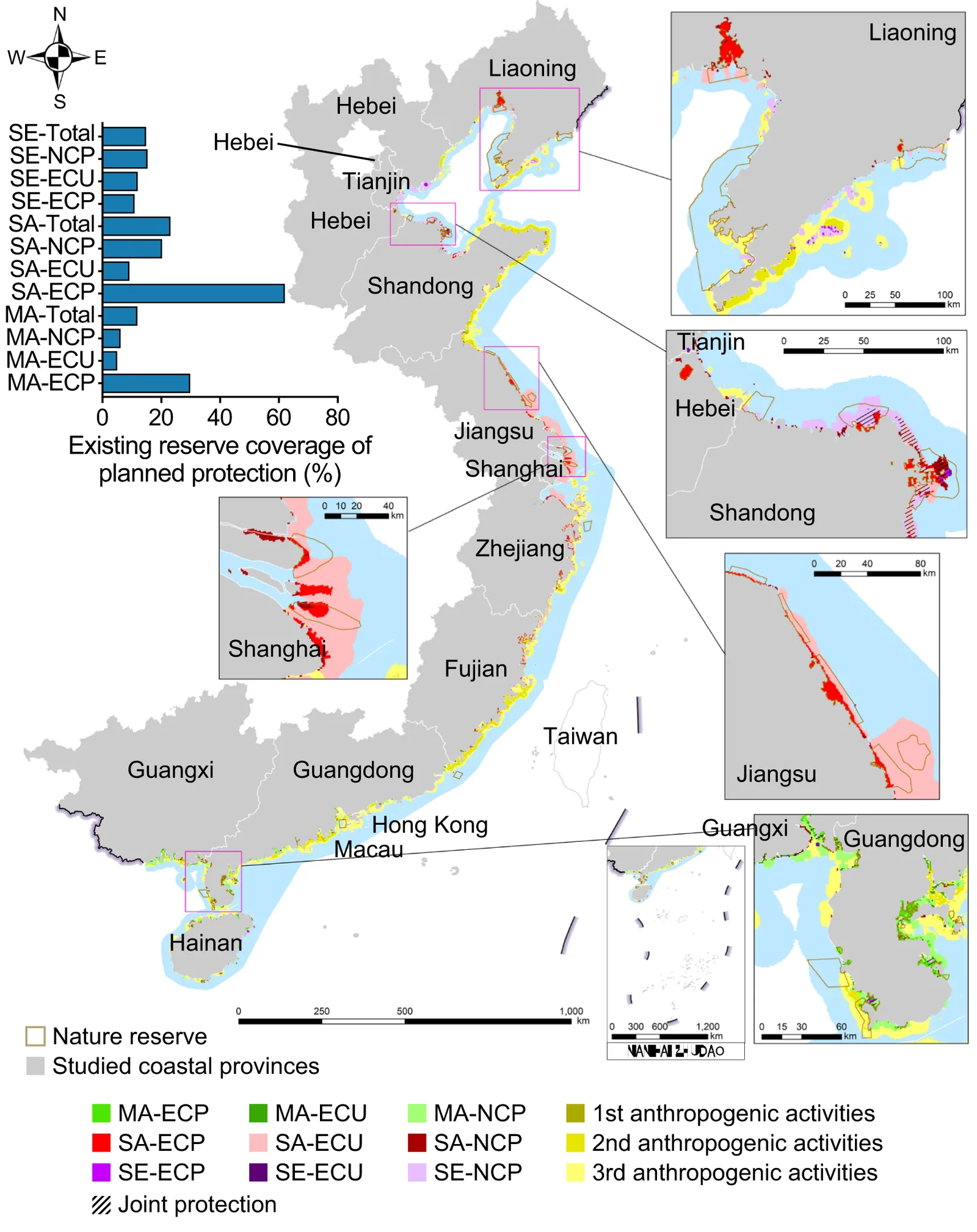
Coverage of planned conservation areas by existing nature reserves. Maps show the spatial distributions of existing nature reserves and planned conservation areas for mangroves, salt marshes, and seagrasses along the study coast; insets enlarge representative coastal regions. The bar chart shows the proportion of each planned conservation category already covered by existing nature reserves, both overall and for individual ecosystem types. MA, mangrove; SA, salt marsh; SE, seagrass; CBCE, coastal blue carbon ecosystem; ECP, existing CBCEs requiring protection; ECU, existing CBCEs not requiring additional protection; NCP, areas without existing CBCEs but identified as conservation priorities; Total, all planned conservation areas within the corresponding ecosystem type. Joint protection zones are areas designated for the protection of two or three coastal blue carbon ecosystem types. Coastal provinces included in this study are shaded grey; Data were unavailable for Taiwan and islands in the South China Sea.

China's existing conservation paradigm largely prioritizes established CBCEs facing substantial degradation risks, reflecting a predominantly reactive approach to ecosystem management [24,25]. However, growing international evidence suggests that reactive conservation alone cannot halt ecosystem decline. Such approaches typically intervene only after significant degradation has occurred, resulting in high restoration costs, prolonged recovery periods, and uncertain ecological outcomes [120,121]. A global synthesis of 954 coastal restoration projects demonstrated that restoring CBCEs is usually expensive and does not consistently recover original ecosystem functions, while preventing degradation is generally more cost-effective than post hoc restoration [122]. Similarly, delayed conservation interventions have been shown to increase management costs while reducing the likelihood of successful ecological recovery [123]. Together, these findings highlight the limitations of restoration-centered conservation strategies and underscore the need for earlier intervention.

By contrast, proactive conservation seeks to identify and safeguard future ecological opportunities before irreversible losses occur. In Europe, managed coastal realignment has been adopted to reserve space for future salt marsh migration as sea levels rise [121]. Along the Gulf Coast of the United States, climate warming has facilitated poleward mangrove expansion, highlighting the need to protect not only existing ecosystems but also future suitable habitats that may support climate-driven range shifts [61]. More broadly, emerging conservation frameworks increasingly distinguish the proactive protection of high-integrity ecosystems from reactive intervention in highly threatened areas. Proactive planning identifies areas where ecosystems can be maintained, restored, or expanded under suitable environmental conditions, whereas reactive intervention targets areas where existing ecosystems face greater anthropogenic pressures [124,125].

The Greater Bay Area in China provides an example where existing conservation initiatives showed substantial spatial agreement with the conservation network identified in this study (Supplementary Fig. S18). This congruence supports the practical relevance of our framework and highlights how spatially explicit planning can inform and optimize project placement, ensuring that limited conservation resources are directed toward areas of greatest ecological potential and need. The proposed conservation network extends beyond existing CBCE distributions by identifying climatically and environmentally suitable areas currently absent from conservation initiatives, representing potential targets for future protection or restoration, particularly in rapidly developing coastal regions. By identifying such areas in advance, proactive conservation planning can enhance the effectiveness, adaptability, and long-term resilience of China's CBCE conservation network under accelerating climatic and anthropogenic pressures.

### 3.6 Typology-based framework for differentiated CBCE governance and policy recommendations

The climatic and anthropogenic pressures identified in this study highlight a broader challenge facing CBCE conservation globally: static protected area networks often cannot accommodate rapidly changing environmental conditions and escalating anthropogenic pressures. In China, where coastal development and climate change occur simultaneously, these challenges reinforce the need for a proactive conservation framework that protects existing ecosystems and anticipates future habitat redistribution, restoration opportunities, and emerging degradation risks. However, traditional protected area planning often relies on assessments of isolated grid cells, which fail to capture the continuity and synergy among ecosystems at landscape scale. Effective conservation requires spatially connected protected areas supported by appropriate management strategies [126]. To support integrated CBCE management, we integrate existing conservation patterns, restoration and degradation intensities, and anthropogenic pressures on CBCEs and identify four types of protection areas (Fig. 6; Supplementary Fig. S19):

**Fig. 6:**
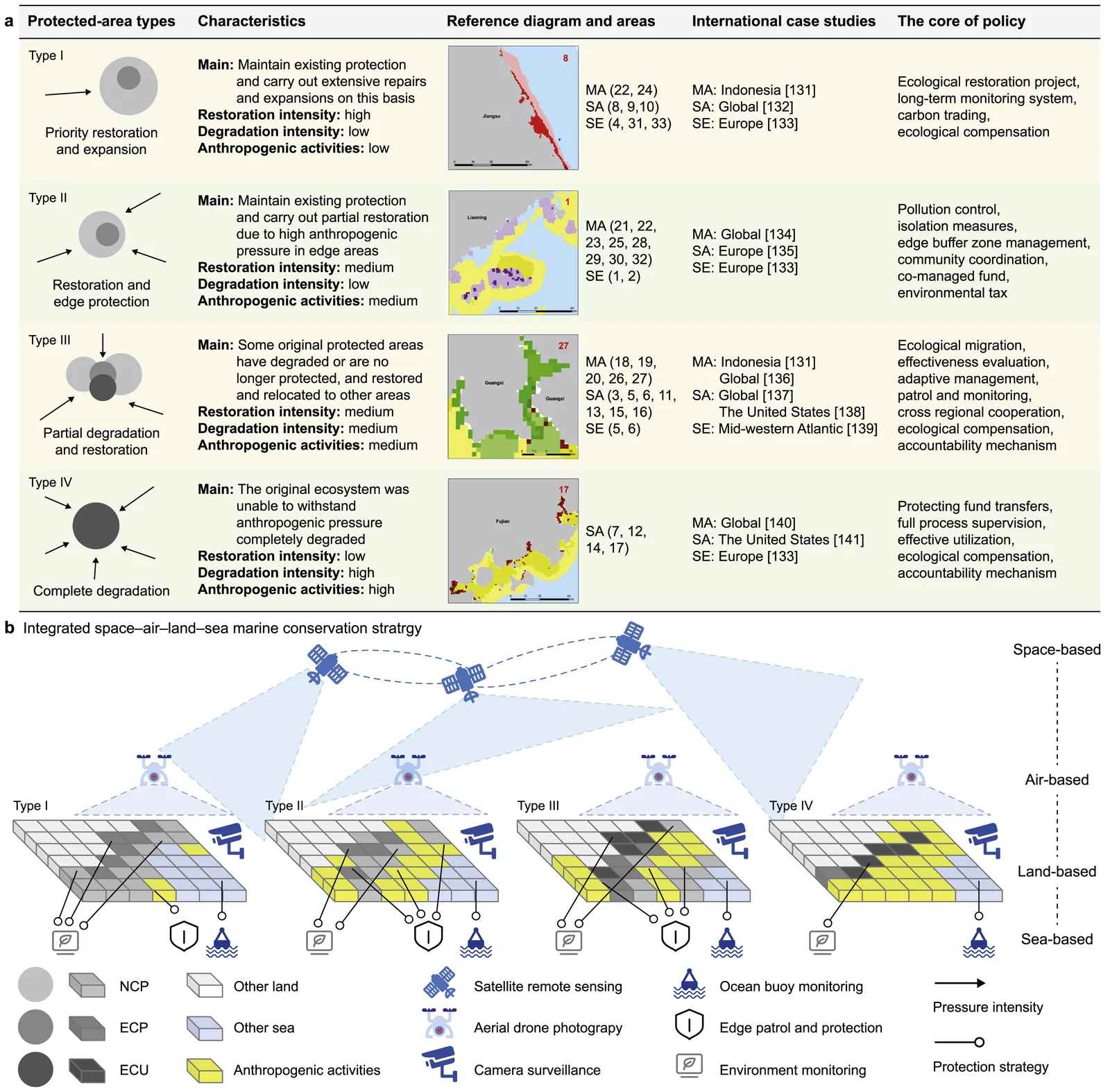
Spatially differentiated conservation strategies for coastal blue carbon ecosystems [[127], [128], [129], [130], [131], [132], [133], [134], [135], [136], [137]]. **a,** Classification framework and corresponding management strategies for four CBCE conservation zones, including priority restoration and expansion, restoration and edge protection, partial degradation and restoration, and complete degradation. Each category integrates ecosystem status, restoration intensity, degradation intensity, and anthropogenic pressure to define management priorities and associated policy approaches. A reference diagram and numbers in parentheses indicate the corresponding core distribution areas, as defined in Supplementary Fig. S19. **b,** Conceptual framework for an integrated space–air–land–sea conservation strategy, combining satellite remote sensing, aerial drone observations, camera surveillance, ocean monitoring, and environmental assessments to support dynamic CBCE management. MA, mangrove; SA, salt marsh; SE, seagrass; CBCE, coastal blue carbon ecosystem; ECP, existing CBCEs requiring protection; ECU, existing CBCEs not requiring additional protection; NCP, areas without existing CBCEs but identified as conservation priorities.

***Priority restoration and expansion*.** These areas center on existing CBCEs, have substantial restoration potential, and experience relatively low anthropogenic pressure. The spatial combination of existing ecosystem distribution, restoration potential, and anthropogenic pressure indicates that these areas are suitable for prioritizing long-term restoration and expansion. Conservation planning in these areas could therefore focus on setting clear restoration targets and identifying sites for ecosystem expansion, while integrating available policy and financing mechanisms, such as carbon-sink trading, to support implementation.

***Restoration and edge protection*.** These areas contain existing CBCEs but experience relatively high anthropogenic pressure at ecosystem boundaries, limiting opportunities for further expansion. Conservation priorities in these regions include implementing strict boundary management to prevent external pollution and, through community coordination and sustained monitoring, maintaining a balance between conservation and development. Such areas are commonly found along the southern coast, including Guangdong, Guangxi, and Hainan, where mangroves dominate.

***Partial degradation and restoration*.** These zones show land–sea divergence in pressure patterns. The landward side faces high ecosystem degradation risks due to land development and intensive anthropogenic activities, while the seaward side offers conditions suitable for restoration. This pattern necessitates flexible protection strategies that address landward threats through regional coordination while guiding adaptive ecosystem expansion toward the seaward side.

***Complete degradation*.** Ecosystems in these areas face comprehensive degradation risks under extremely high anthropogenic pressure, particularly in salt marsh distribution zones in Zhejiang, Shandong, and Fujian. Conventional protection approaches may be difficult to implement under these conditions. The current focus should shift toward improving ecological monitoring and damage assessment, exploring feasible ecological compensation mechanisms, and reallocating funds to address the conflict between conservation and development [138].

Given constraints on expanding protected area networks, such as limited funding and management inefficiencies, improving the effectiveness of protection areas through updated management frameworks, better monitoring, and alignment with local community needs is critical [139]. Building on the zoning strategies identified here, we proposed an integrated space–air–land–sea monitoring and management framework to further support CBCE conservation (Fig. 6b). The core of this framework involves the development of a high-resolution, real-time marine environment and ecological monitoring network to dynamically assess the impact of key factors (e.g., salinity and temperature) on CBCEs and provide early warnings of potential risks.

Coastal development activities (e.g., mariculture and port engineering activities) should incorporate environmentally friendly technologies (e.g., recirculating mariculture systems and eco-friendly disposal of dredged material) and spatial planning approaches that maintain the natural connectivity and ecological integrity of coastal wetlands [120]. Management should also consider species interactions, such as competition, facilitation, and succession dynamics, within the context of joint conservation and ecological restoration. Establishing a coordinated monitoring and control mechanism for invasive species, such as Spartina alterniflora, is vital to protect native ecosystem stability and ensure the effectiveness of restoration efforts [140]. Ultimately, grid-based management should be enhanced, and a national-scale CBCE monitoring and assessment system should be established by integrating multisource monitoring data and formulating unified standards. Regular reporting can provide enduring support for dynamically adjusting conservation strategies and increasing the adaptability and scientific rigor of ocean governance [141].

### 3.7 Economic feasibility of implementing the proposed conservation strategy

To evaluate the economic feasibility of implementing the proposed conservation network, we estimated restoration costs and potential carbon sequestration values under a nature-based solutions framework [142,143] (Supplementary Text S2). Achieving the 30% conservation target would require an investment of approximately USD 117 billion, consistent with the investment level needed for global blue carbon targets [125]. Upon implementation, annual carbon sequestration could increase by USD 21.33–25.87 million tons, corresponding to a long-term carbon value of approximately USD 2.77–3.36 billion per year, with contributions from mangroves, salt marshes, and seagrass areas in a ratio of approximately 5:23:2 (Supplementary Table S17). Although the annual carbon sequestration value alone is lower than the estimated restoration cost, this comparison should not be interpreted as a formal cost–benefit assessment. Rather, it provides a preliminary indication of the economic feasibility of implementing large-scale CBCE conservation under a blue carbon framework. Carbon sequestration is a long-term, continuously accruing ecosystem benefit that depends on sustained ecosystem integrity. Moreover, our assessment does not include the substantial economic benefits generated by other ecosystem services, such as coastal protection, biodiversity maintenance, water purification, and enhanced social well-being, which often exceed the value of carbon storage in coastal socioecological systems [144]. In addition, carbon valuation remains dynamic and may rise under stricter climate policies, enhancing future cost recovery.

A comprehensive evaluation of CBCE conservation therefore requires consideration of both market and non-market values of ecosystem services across temporal and spatial scales. This perspective reinforces the growing recognition that conservation represents a long-term socioecological investment capable of generating sustained climate, ecological, and societal returns. Furthermore, recent evidence shows that the benefits and costs of protected areas often extend beyond their formal boundaries, generating significant ecological and socioeconomic spillover effects [145]. Therefore, future conservation planning and evaluation must rely on a landscape–seascape approach that accounts for these transboundary flows, thereby ensuring that protection networks are designed not only to maximize ecological integrity but also to enhance systemic resilience, equitable benefit sharing, and long-term sustainability.

### 3.8 Limitations and future directions

This study has several limitations. First, the CBCE distribution data represent specific time periods and may not fully capture interannual dynamics or responses to anomalous events. For example, temporary reductions in anthropogenic activities, such as those associated with the COVID-19 pandemic, may have produced short-term effects. Future studies integrating long-term remote sensing and field observations could better characterize ecosystem dynamics.

Second, the conservation strategies developed here primarily considered ecological dimensions, while socioeconomic factors that influence implementation feasibility, including land tenure, current land-use intensity, and local livelihood dependencies, were not explicitly incorporated. Integrating these dimensions would improve the practical feasibility and long-term sustainability of restoration actions, such as wetland reclamation in densely developed coastal regions.

Third, although this study aimed to characterize broad-scale environmental suitability and ecological niches rather than short-term ecosystem dynamics, temporal mismatches existed between some predictor datasets and CBCE distribution records because consistent national-scale datasets were limited. Nevertheless, the selected variables were intended to represent long-term climatic conditions, relatively stable environmental attributes, or multi-year oceanographic backgrounds that are considered major determinants of ecosystem distributions. Future research could further improve predictions by incorporating dynamic environmental variables and modeling ecosystem responses under environmental change and human activities.

## 4 Conclusions

As nations worldwide strive to meet the Kunming–Montreal Global Biodiversity Framework and climate mitigation targets under the Paris Agreement, spatially explicit planning can help identify areas with different conservation and restoration priorities for CBCEs. Our study showed that effective CBCE conservation depends on a dual strategy: protecting existing high-value ecosystems from escalating anthropogenic pressures while strategically restoring habitats in areas of high environmental suitability and low socioecological conflict. By integrating ecosystem modeling, pressure mapping, and SCP approaches, we provide a scalable framework to inform national policies and support transboundary collaboration on coastal resilience. These results can support the spatial allocation of conservation and restoration efforts and provide a basis for incorporating ecological and anthropogenic constraints into CBCE management planning.

## CRediT authorship contribution statement

**Yang Xu:** Writing – review & editing, Writing – original draft, Visualization, Validation, Software, Methodology, Investigation, Formal analysis, Data curation, Conceptualization. **Tianci Gu:** Writing – review & editing, Validation. **Feng Xu:** Writing – review & editing, Validation, Supervision, Resources, Project administration, Methodology, Formal analysis, Conceptualization. **Lu Xing:** Investigation, Formal analysis, Data curation. **Guangqing Chi:** Validation, Supervision. **Ruibo Qiu:** Writing – review & editing. **Chunyang He:** Validation, Supervision. **Yangfan Li:** Writing – review & editing, Validation, Supervision, Data curation, Conceptualization. **Qingxu Huang:** Writing – review & editing, Supervision, Resources, Project administration, Methodology, Funding acquisition, Conceptualization.

## Data availability

The data on the Spatially Differentiated Conservation and Management Strategies for Coastal Blue Carbon Ecosystems in China generated in this study are publicly available on GitHub at https://github.com/q22huang/Spatially-Differentiated-Conservation-Strategies.

## Declaration of competing interest

The authors declare that they have no known competing financial interests or personal relationships that could have appeared to influence the work reported in this paper.
