# Supplementary material for: Dual-constraint spatial planning unlocks proactive blue carbon expansion across China's coastlines
Source: Environ Sci Ecotechnol. 2026 Sep 26;34:100771. doi: 10.1016/j.ese.2026.100771 (PMC13657239; doi:10.1016/j.ese.2026.100771)
Supplement: Supplementary file 1 [file mmc1.pdf]

Supplementary information

Dual-constraint spatial planning unlocks proactive blue carbon expansion across China's coastlines

Yang Xu[a,b], Tianci Gu[a,b], Feng Xu[c]*, Lu Xing[c], Guangqing Chi[d], Ruibo Qiu[a,e], Chunyang He[a,e], Yangfan Li[f], Qingxu Huang[a,b]*

[a] State Key Laboratory of Earth Surface Processes and Disaster Risk Reduction, Beijing Normal University, Beijing, 100875, China

[b] School of Natural Resources, Faculty of Geographical Science, Beijing Normal University, Beijing, 100875, China

[c] Department of Land Resource Management, China University of Geosciences, Wuhan, 430074, China

[d] Department of Geography, Indiana University, Indiana, 47405, the United States

[e] Academy of Disaster Reduction and Emergency Management, Ministry of Emergency Management and Ministry of Education, Beijing, 100875, China

[f] State Key Laboratory of Marine Environmental Science, Key Laboratory of Coastal and Wetland Ecosystems (Ministry of Education), College of the Environment and Ecology, Xiamen University, Xiamen, 361005, China

Summary Information:

Number of pages: 46

Supplementary Fig. S1-S19

Supplementary Table S1-S15

Supplementary Text S1

Supplementary Table S16

Supplementary Fig. S20 and S21

Supplementary Text S2

Supplementary Table S17

Supplementary References

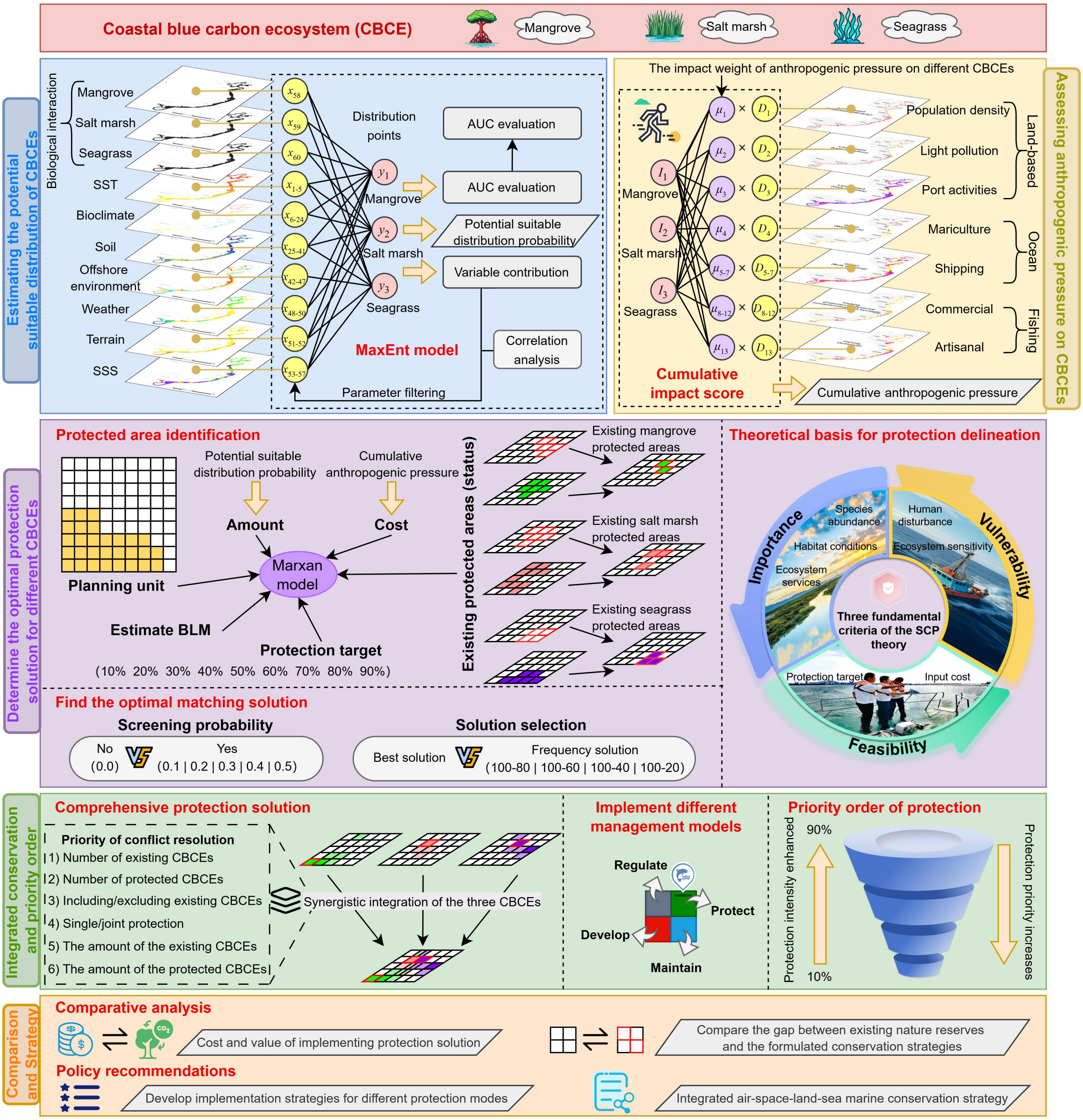

Supplementary Fig. S1 Conceptual framework.

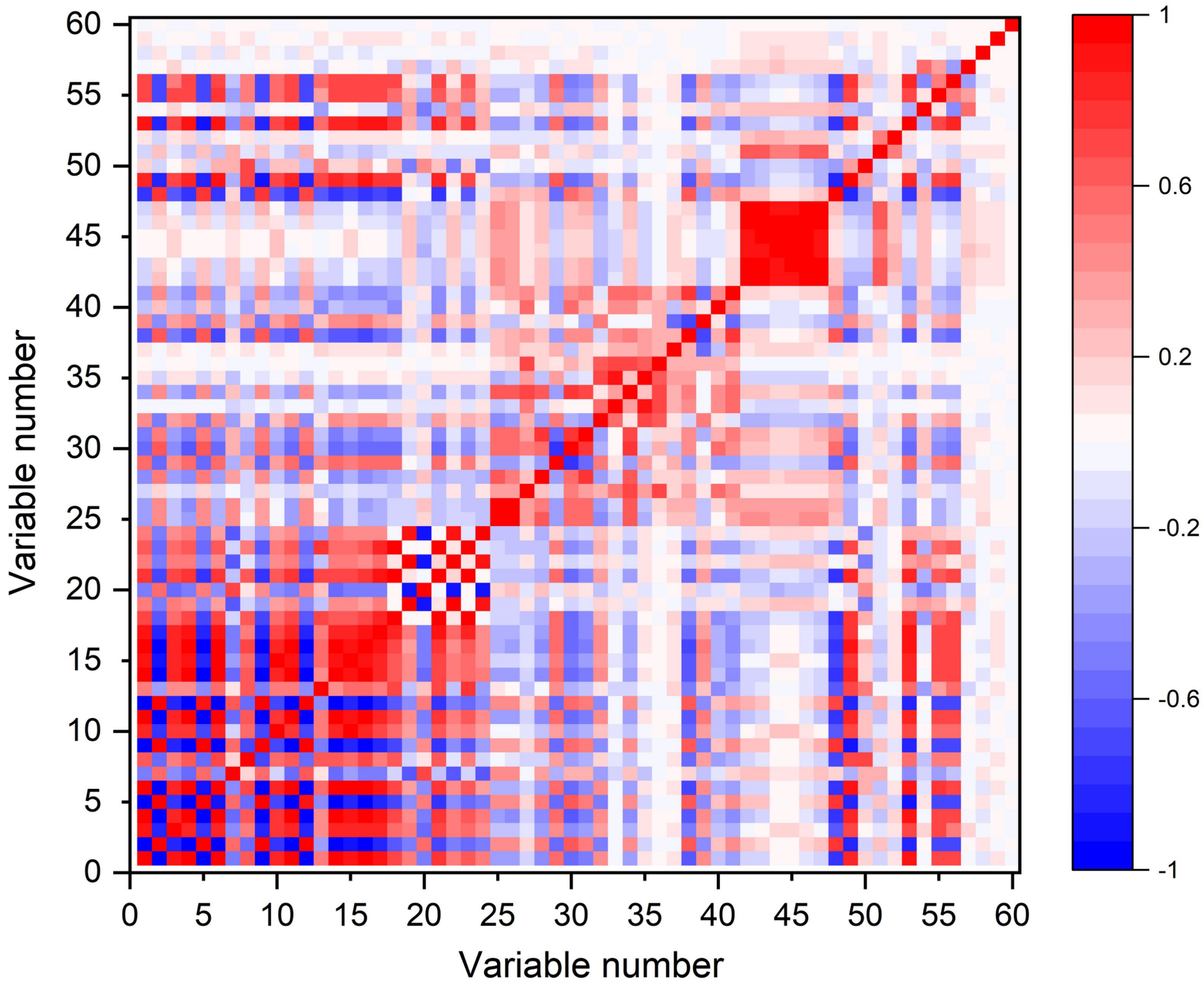

Supplementary Fig. S2 Correlation coefficient.

Note: Variable numbers can be found in **Supplementary Table S4**.

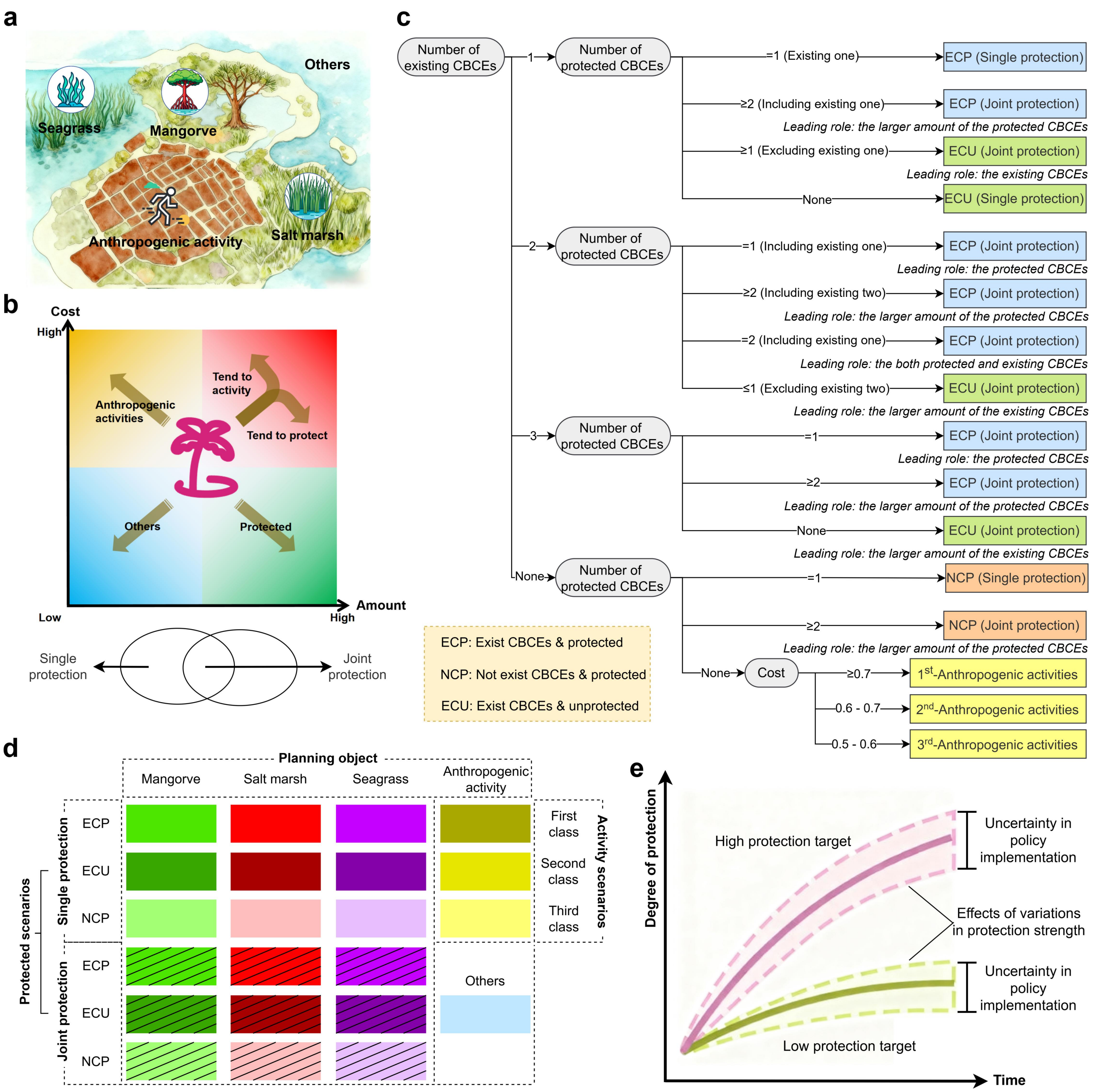

Supplementary Fig. S3 Final scheme design.

Note: a represents the four objects of the final PU division; b represents the governance strategy direction and different types of protection strategies for the final PU; c represents the basis and roadmap for the specific planning and strategy division; d represents the division and coloring of different planning and strategies; e represents the effectiveness under different protection targets.

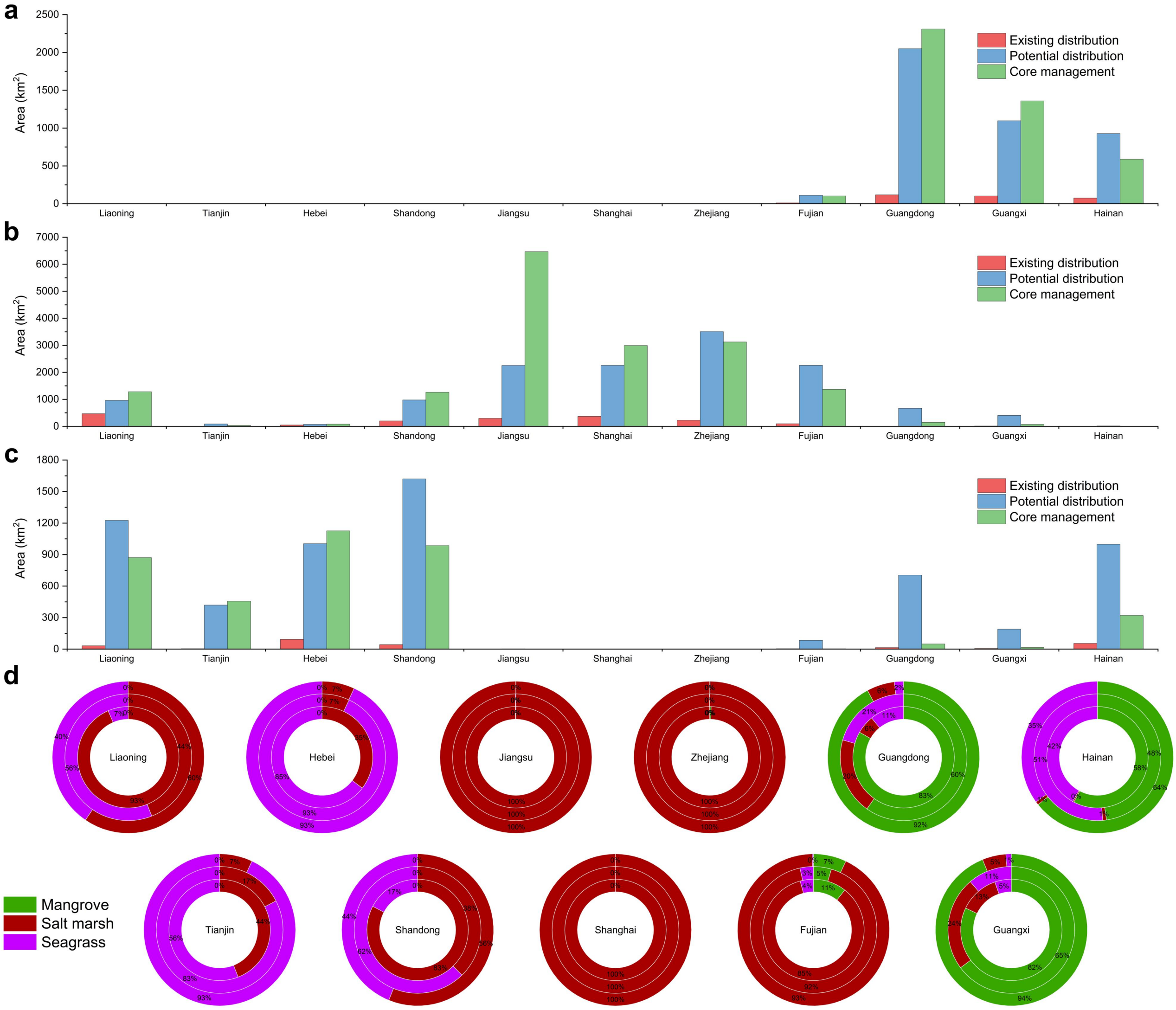

Supplementary Fig. S4 Existing distribution, potential distribution, and core management area of mangrove (a), salt marsh (b), and seagrass (c) in each province, as well as structural changes in each province (d).

Note: The circular diagram shows the existing distribution, potential distribution, and core management of CBCE structure from inside to outside.

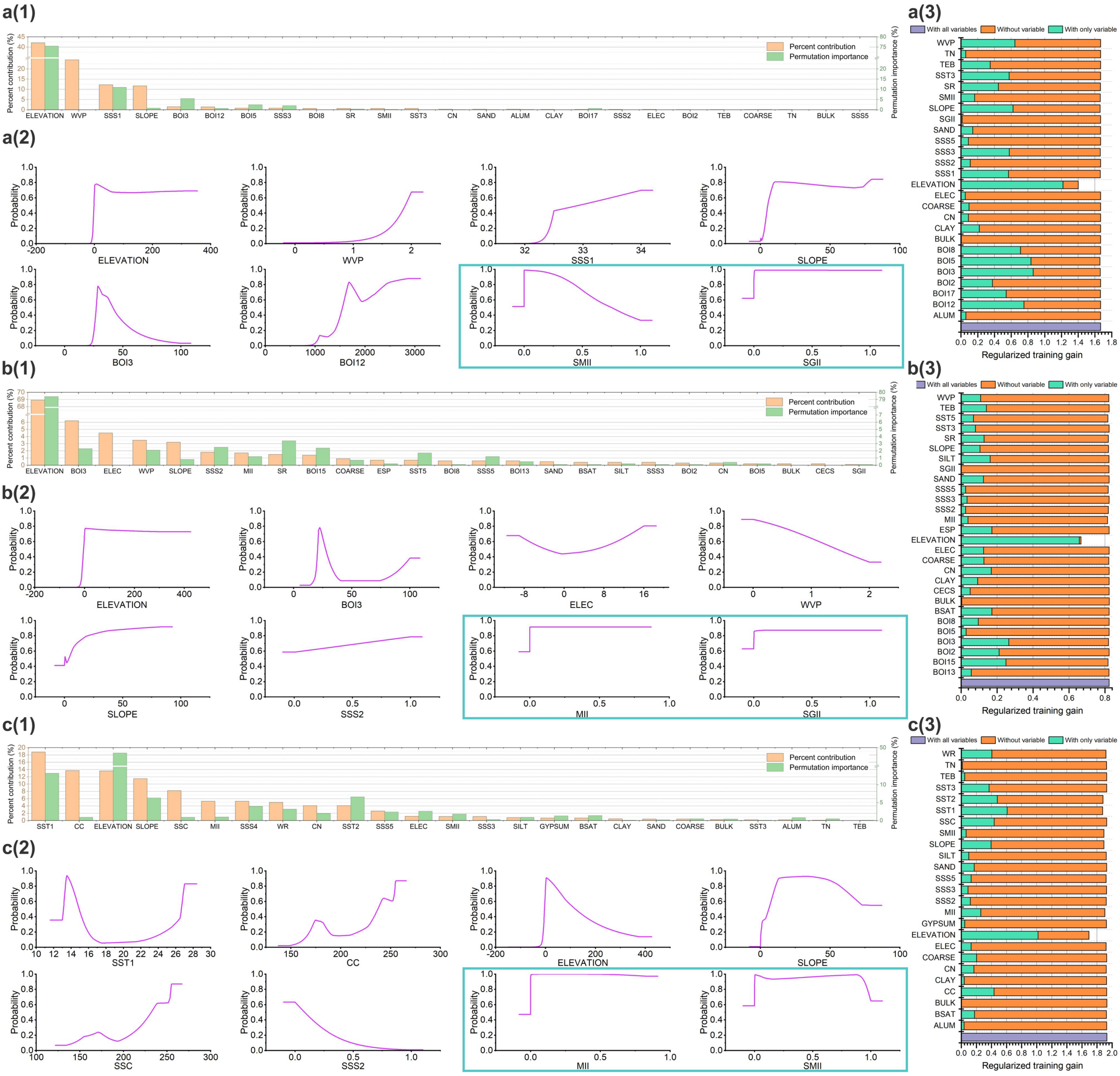

Supplementary Fig. S5 The contribution and response curves of environmental variables to mangrove (a), salt marsh (b), and seagrass (c).

Note: (1) displays the percent contribution and permutation importance of the top 25 variable; (2) displays the response curves of the top 6 percent contributions and biotic interactions; (3) displays regularized training gain of the variable.

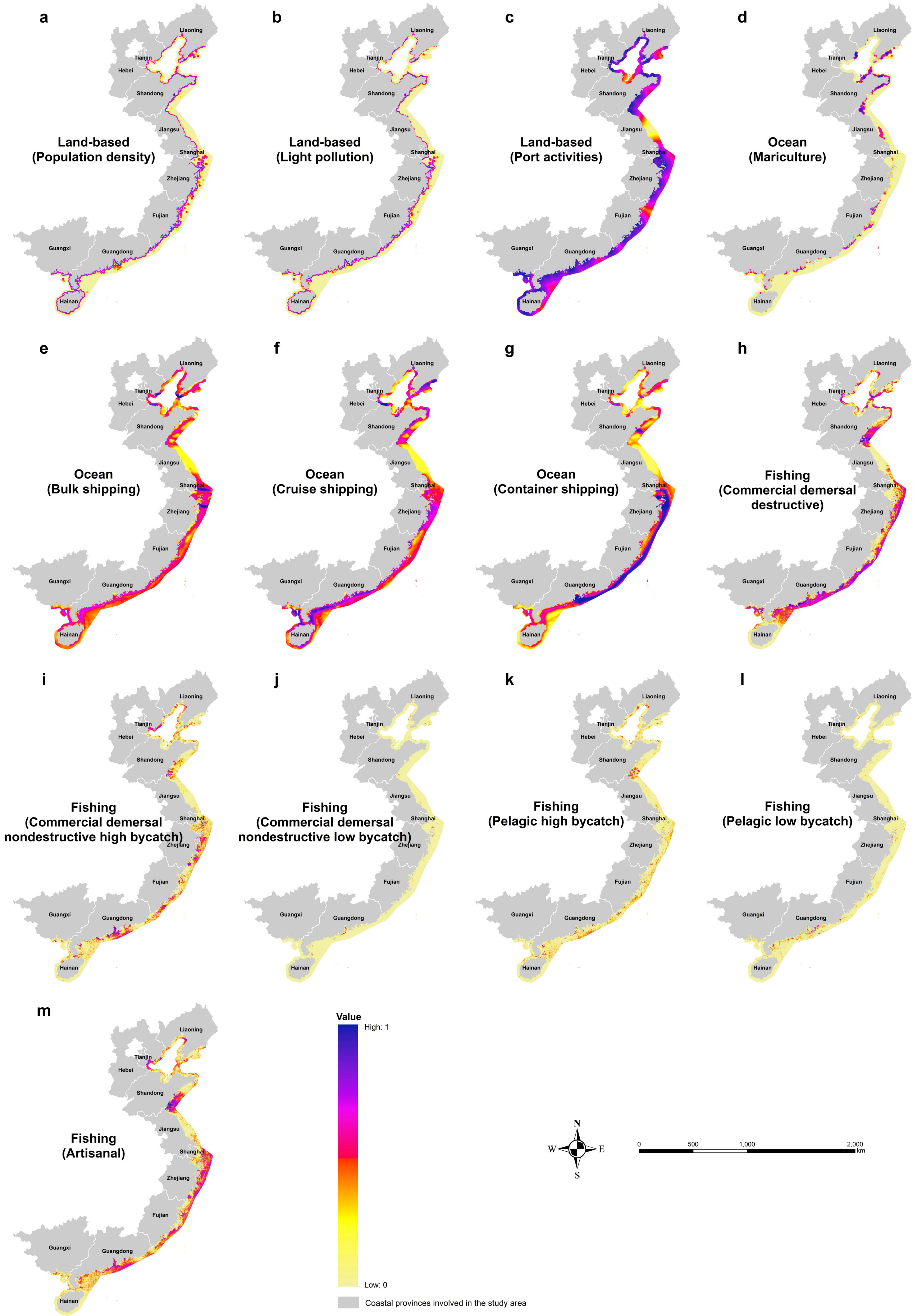

Supplementary Fig. S6 Spatial distribution of various pressure sources (a-m).

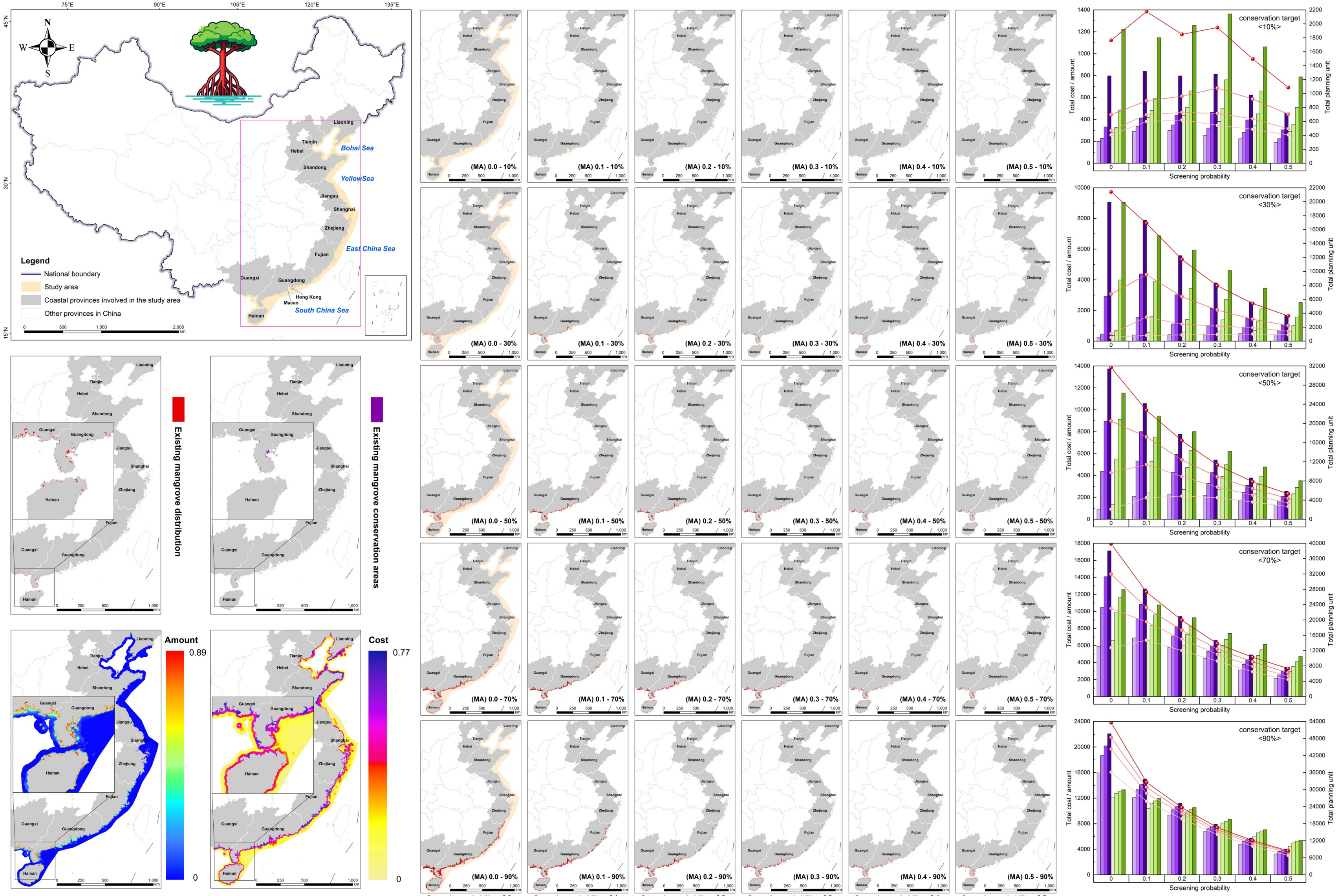

	Screening probability <0.0>	Screening probability <0.1>	Screening probability <0.2>	Screening probability <0.3>	Screening probability <0.4>	Screening probability <0.5>	
Total planning unit	398362 (100%)	35080 (8.81%)	25213 (6.33%)	17561 (4.41%)	12537 (3.15%)	8592 (2.16%)	R1 R2 R3 R4
Total cost	113760.23 (100%)	16107.25 (14.16%)	11940.85 (10.50%)	8374.30 (7.36%)	5980.06 (5.26%)	4090.84 (3.60%)	R1 R2 R3 R4
Total amount	13835.94 (100%)	12343.98 (89.22%)	10917.31 (78.91%)	8999.14 (65.04%)	7259.56 (52.47%)	5488.45 (39.67%)	R1 R2 R3 R4

Supplementary Fig. S7 Comparison of mangrove protected area delineation under different schemes and protection targets.

Note: The upper-left panel depicts the study area and an enlarged view of the coastal region. The lower-left panels show the current mangrove distribution, existing mangrove conversation areas, ecosystem suitability, and anthropogenic pressure. The central panels illustrate spatial selection frequency solutions under varying screening probability thresholds (0.0, 0.1, 0.2, 0.3, 0.4, and 0.5) and conservation targets (10%, 30%, 50%, 70%, and 90%). The table beneath summarizes the total number of planning units, total cost, and total amount for each screening probability threshold. The right-hand panels compare the corresponding total cost/amount, and planning units under different conservation targets and screening probability thresholds.

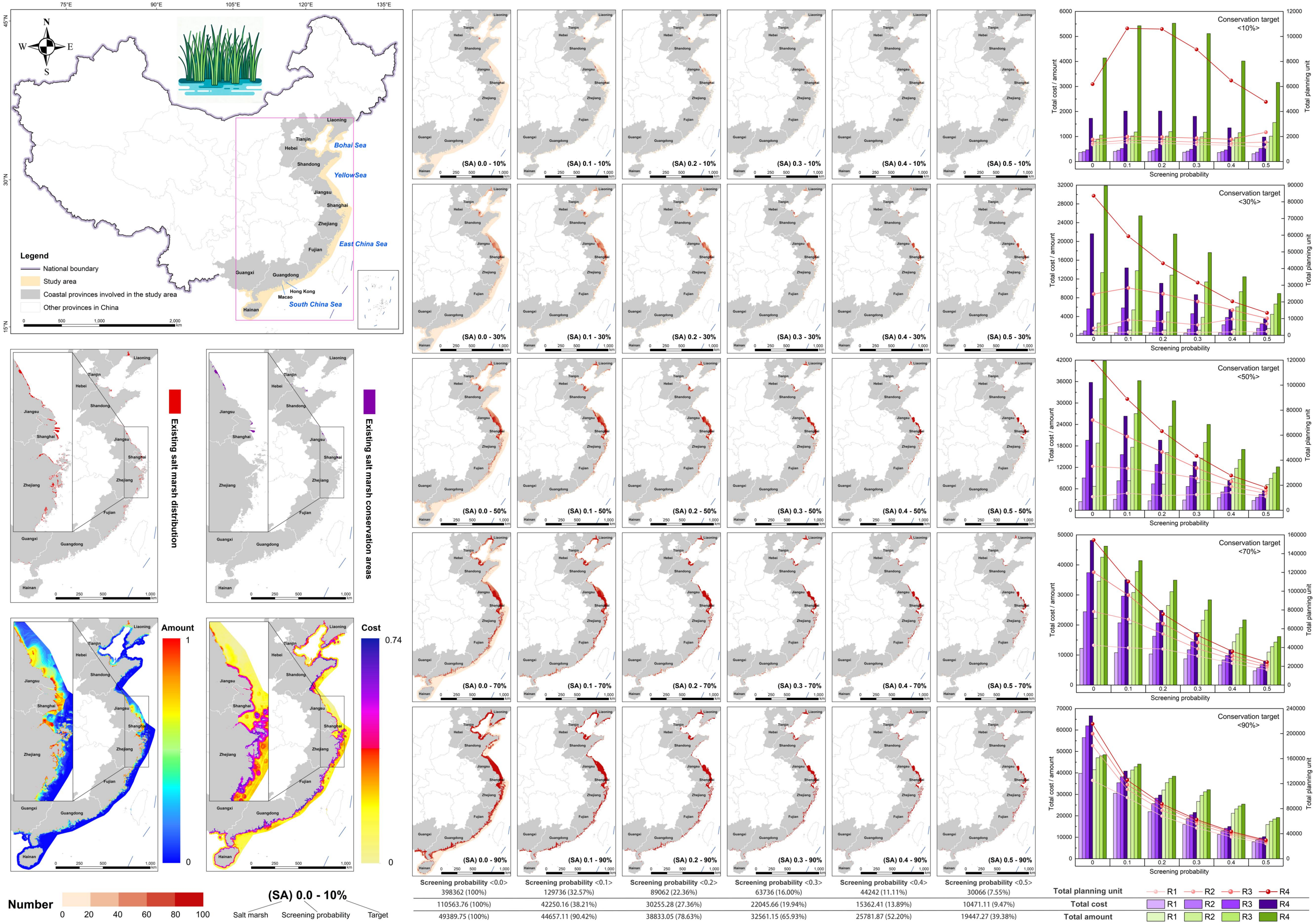

	Screening probability <0.0>	Screening probability <0.1>	Screening probability <0.2>	Screening probability <0.3>	Screening probability <0.4>	Screening probability <0.5>
Total planning unit	398362 (100%)	129736 (32.57%)	89062 (22.36%)	63736 (16.00%)	44242 (11.11%)	30066 (7.55%)
Total cost	110563.76 (100%)	42250.16 (38.21%)	30255.28 (27.36%)	22045.66 (19.94%)	15362.41 (13.89%)	10471.11 (9.47%)
Total amount	49389.75 (100%)	44657.11 (90.42%)	38833.05 (78.63%)	32561.15 (65.93%)	25781.87 (52.20%)	19447.27 (39.38%)

Supplementary Fig. S8 Comparison of salt marsh protected area delineation under different schemes and protection targets.

Note: The upper-left panel depicts the study area and an enlarged view of the coastal region. The lower-left panels show the current salt marsh distribution, existing salt marsh conversation areas, ecosystem suitability, and anthropogenic pressure. The central panels illustrate spatial selection frequency solutions under varying screening probability thresholds (0.0, 0.1, 0.2, 0.3, 0.4, and 0.5) and conservation targets (10%, 30%, 50%, 70%, and 90%). The table beneath summarizes the total number of planning units, total cost, and total amount for each screening probability threshold. The right-hand panels compare the corresponding total cost/amount, and planning units under different conservation targets and screening probability thresholds.

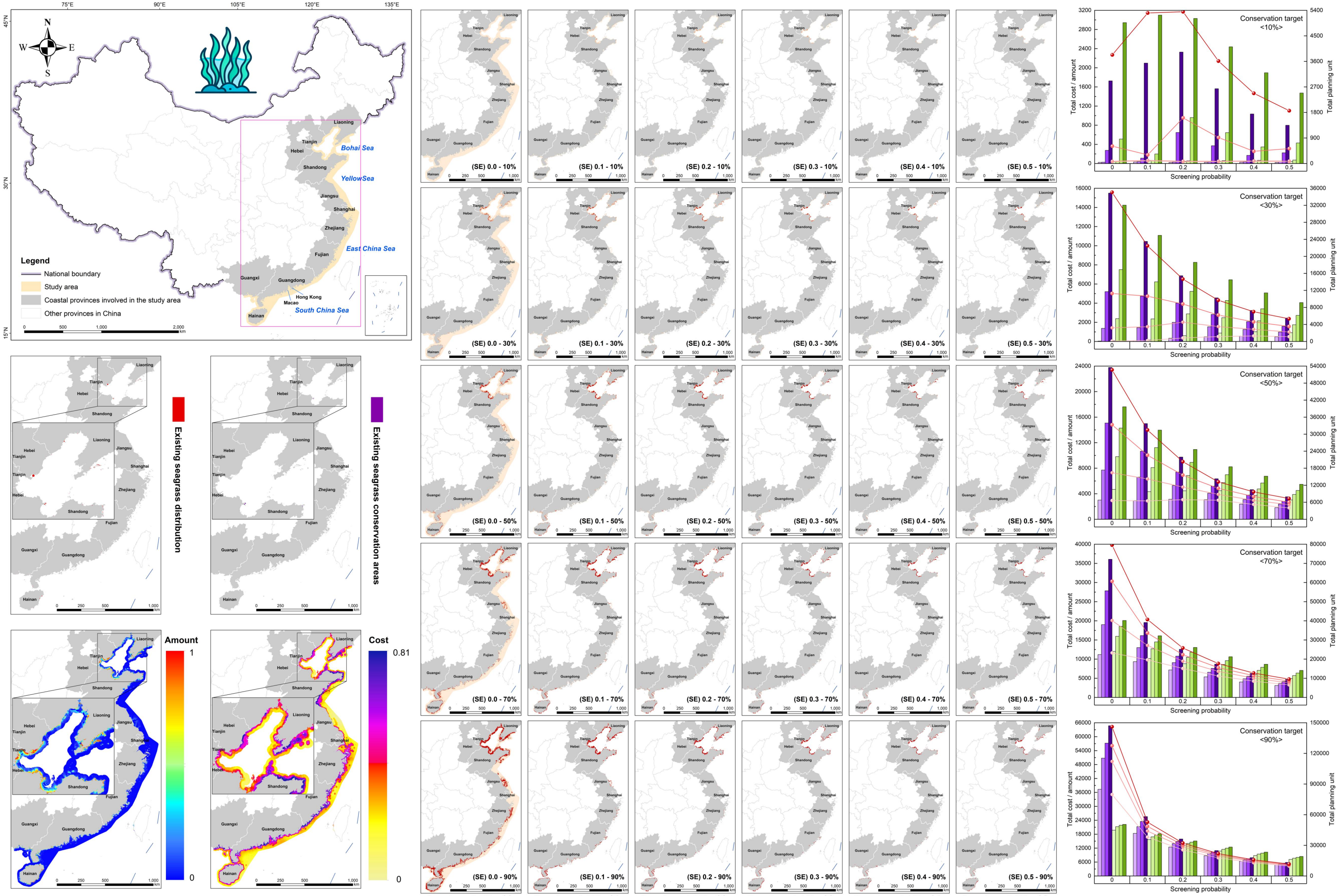

	Screening probability <0.0>	Screening probability <0.1>	Screening probability <0.2>	Screening probability <0.3>	Screening probability <0.4>	Screening probability <0.5>
Total planning unit	398362 (100%)	56392 (14.16%)	34233 (8.59%)	23003 (5.77%)	16430 (4.12%)	12058 (3.03%)
Total cost	154311.42 (100%)	27417.05 (17.77%)	16987.61 (11.01%)	11509.22 (7.46%)	8230.14 (5.33%)	6029.30 (3.91%)
Total amount	22963.01 (100%)	18722.63 (81.53%)	15577.62 (67.84%)	12817.72 (55.82%)	10540.24 (45.90%)	8579.42 (37.36%)

Supplementary Fig. S9 Comparison of seagrass protected area delineation under different schemes and protection targets.

Note: The upper-left panel depicts the study area and an enlarged view of the coastal region. The lower-left panels show the current seagrass distribution, existing seagrass conversation areas, ecosystem suitability, and anthropogenic pressure. The central panels illustrate spatial selection frequency solutions under varying screening probability thresholds (0.0, 0.1, 0.2, 0.3, 0.4, and 0.5) and conservation targets (10%, 30%, 50%, 70%, and 90%). The table beneath summarizes the total number of planning units, total cost, and total amount for each screening probability threshold. The right-hand panels compare the corresponding total cost/amount, and planning units under different conservation targets and screening probability thresholds.

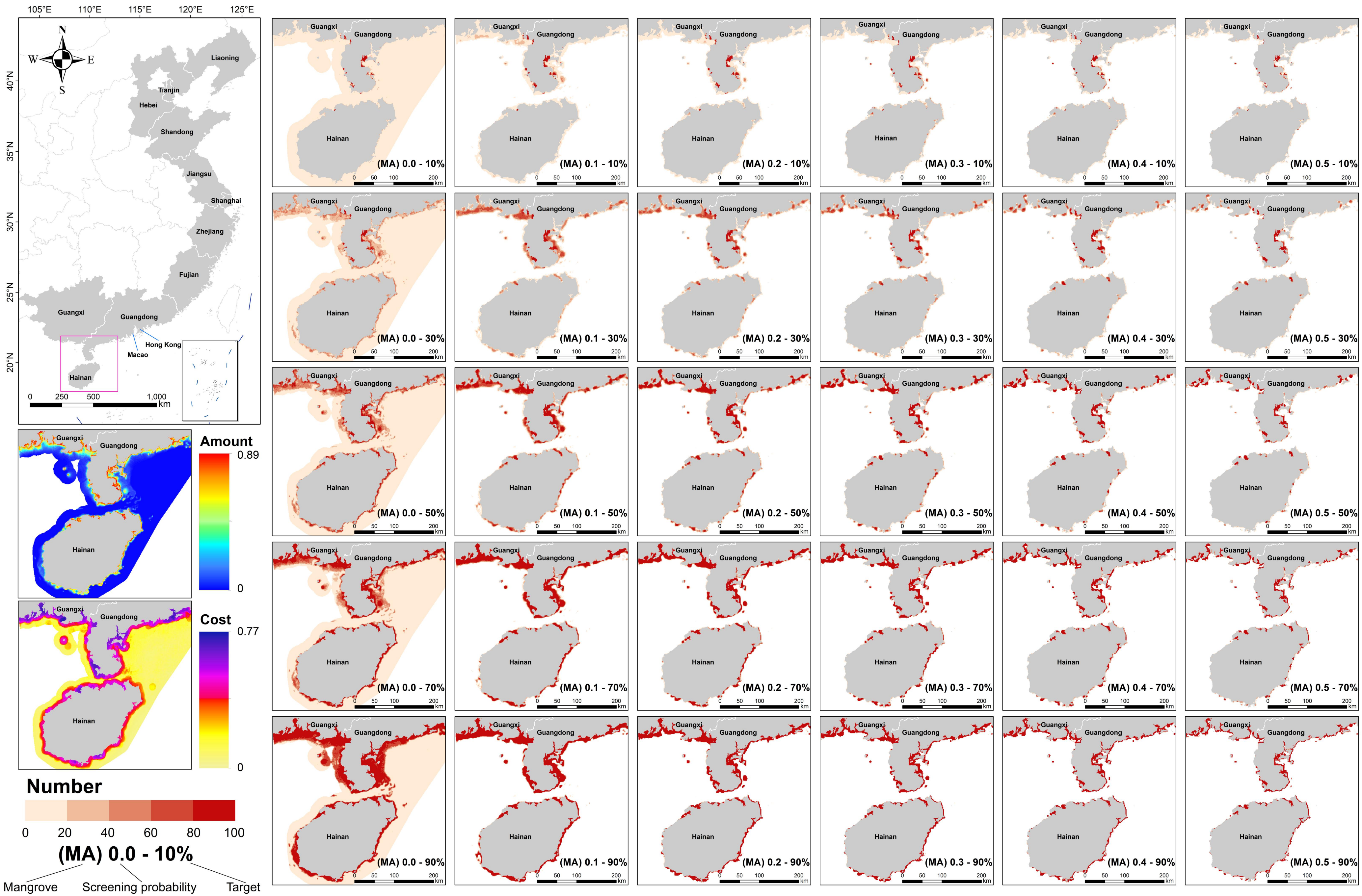

Supplementary Fig. S10 Comparison of mangrove protected area delineation under different schemes and protection targets (partial map).

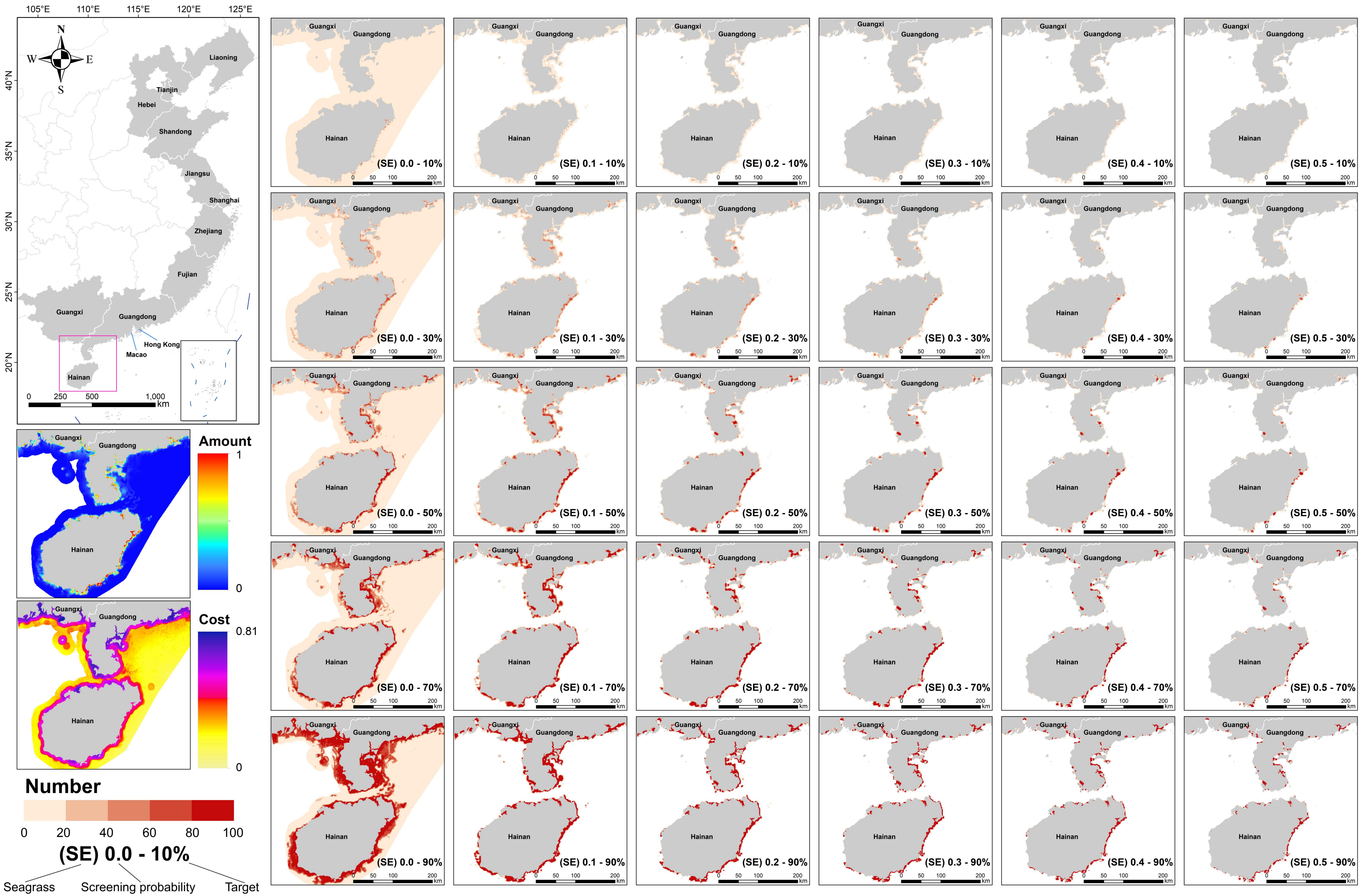

Supplementary Fig. S11 Comparison of seagrass protected area delineation under different schemes and protection targets (partial map).

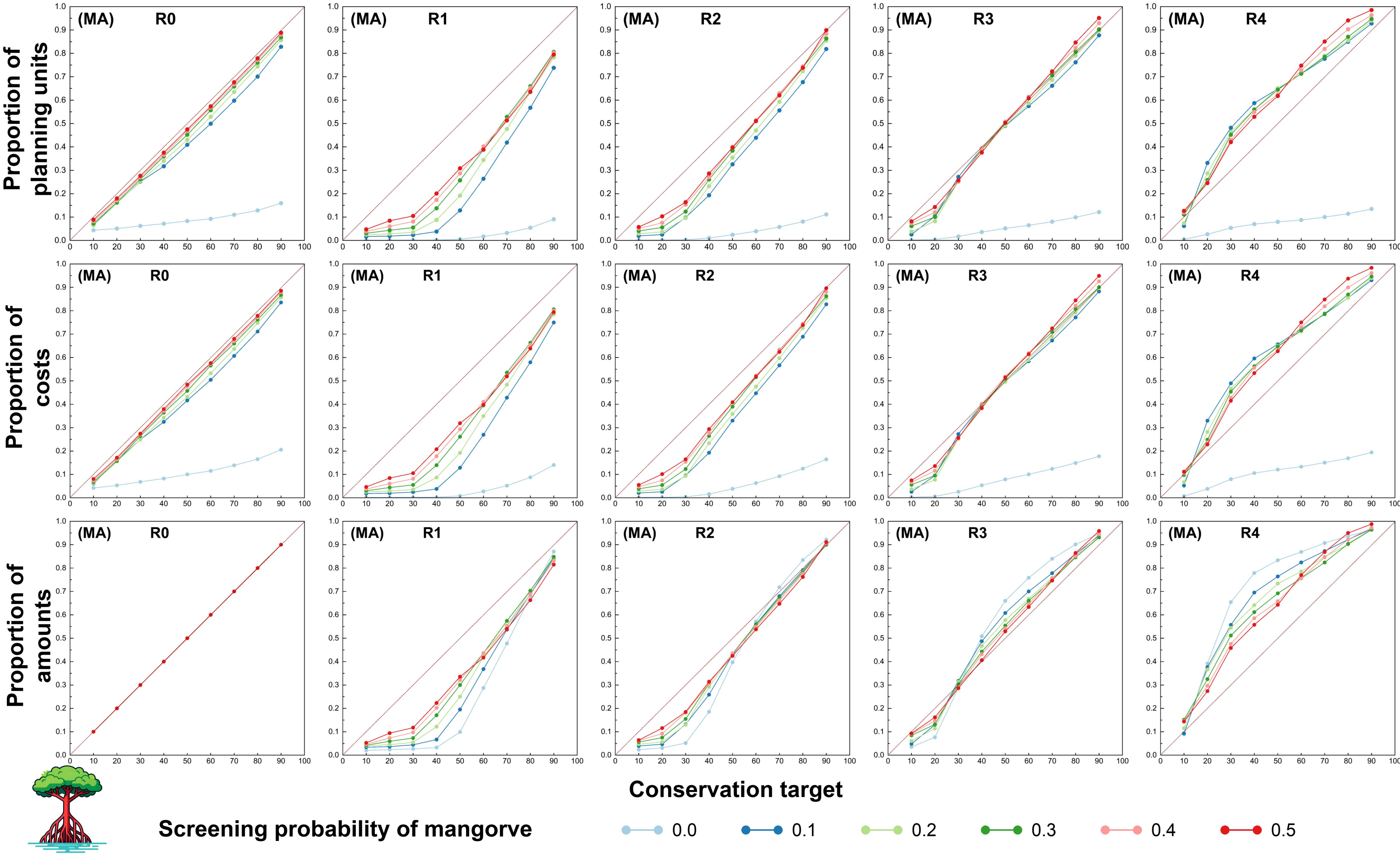

Supplementary Fig. S12 Comparison of the proportion of amounts, costs, and planning units under different schemes and protection targets (mangrove).

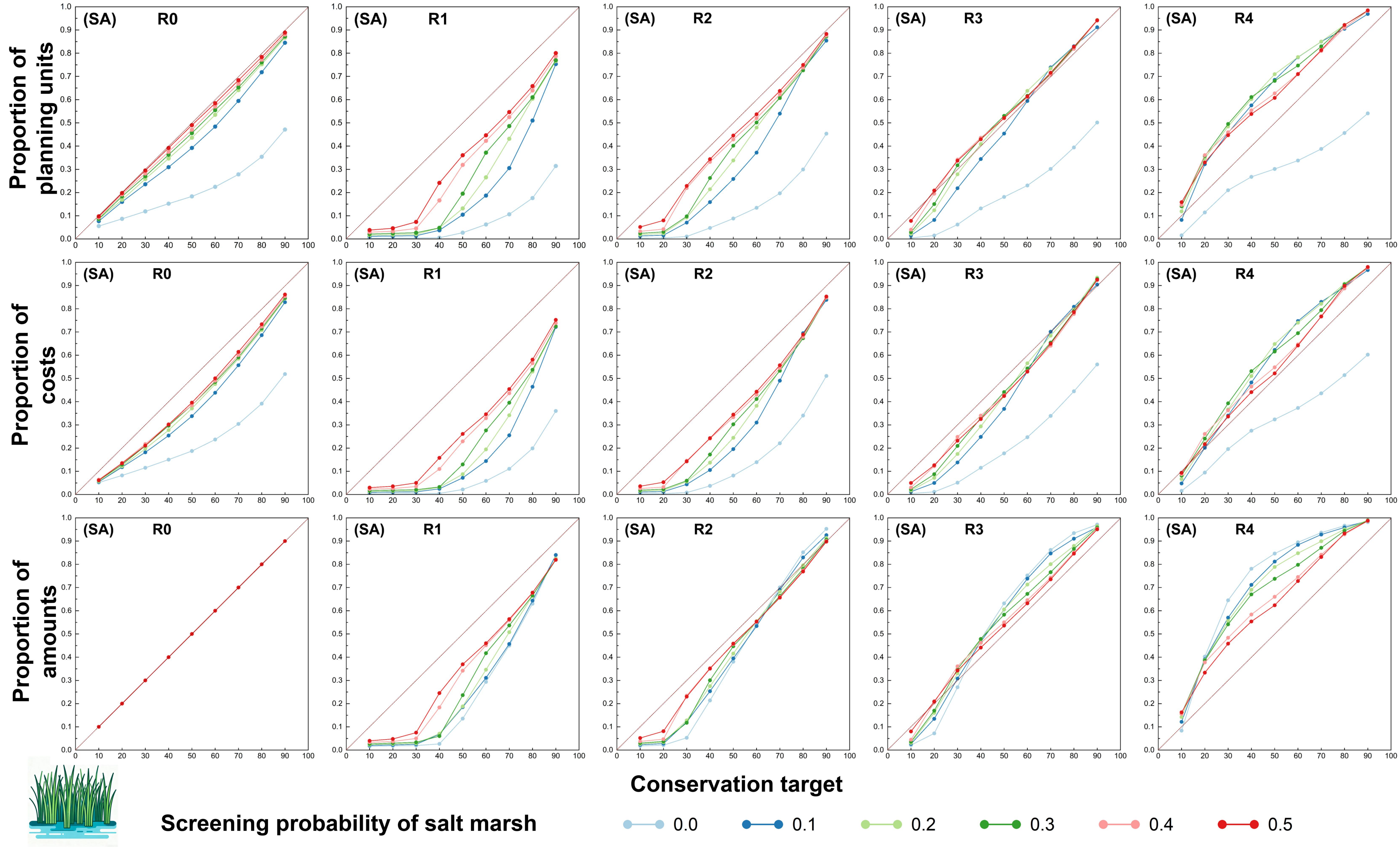

Supplementary Fig. S13 Comparison of the proportion of amounts, costs, and planning units under different schemes and protection targets (salt marsh).

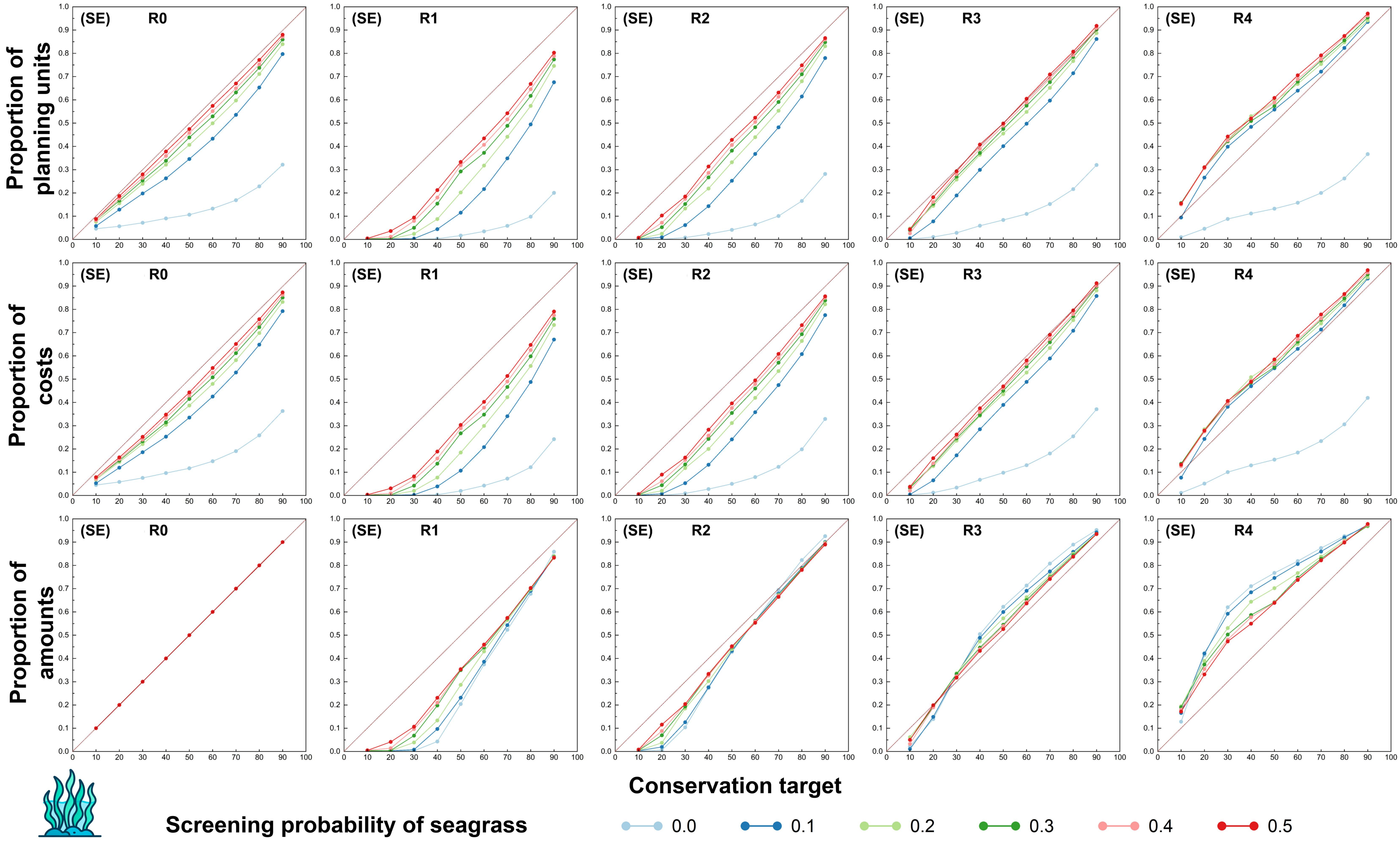

Supplementary Fig. S14 Comparison of the proportion of amounts, costs, and planning units under different schemes and protection targets (seagrass).

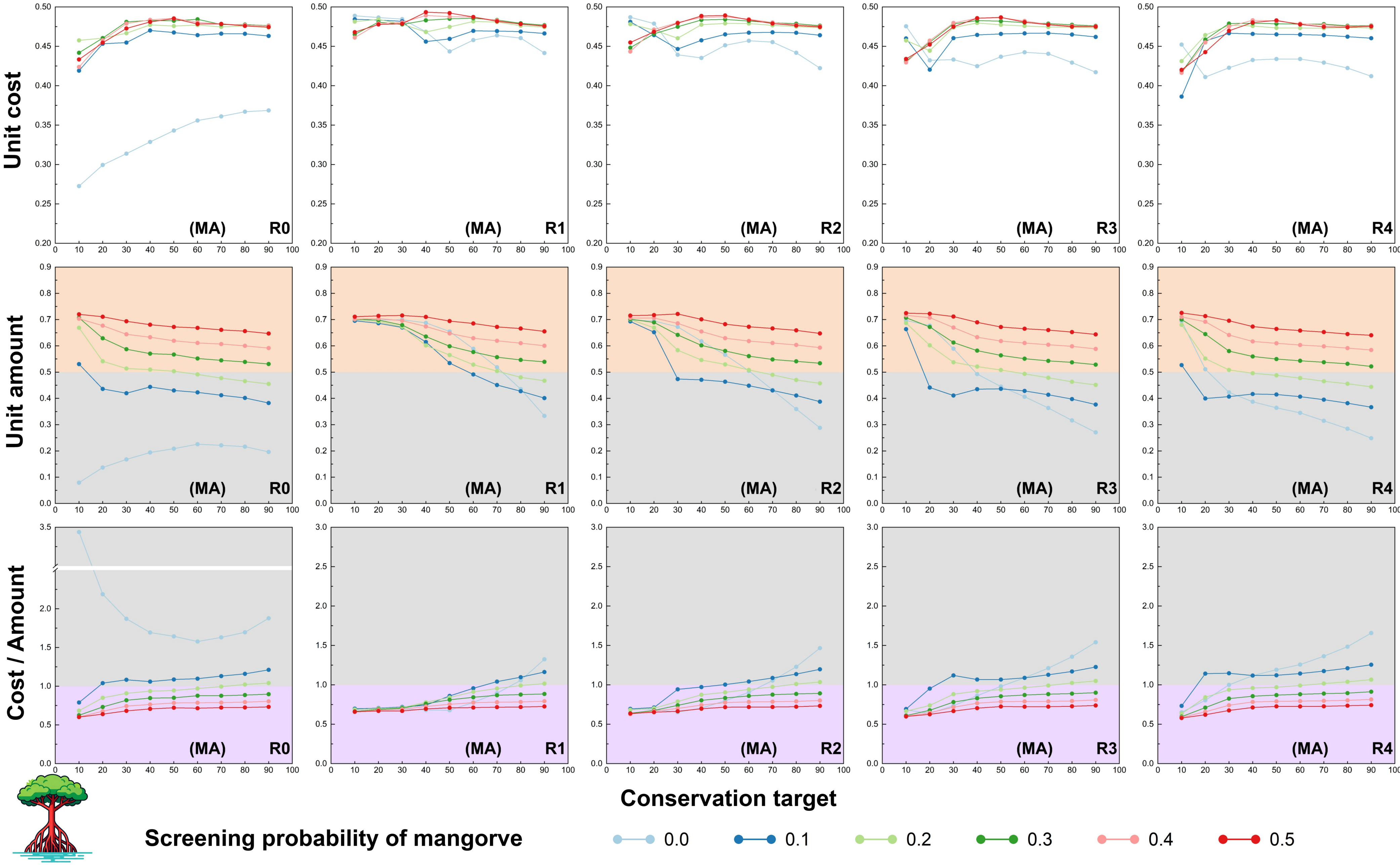

Supplementary Fig. S15 Comparison of unit amount, unit cost, and unit amount cost under different schemes and protection targets (mangrove).

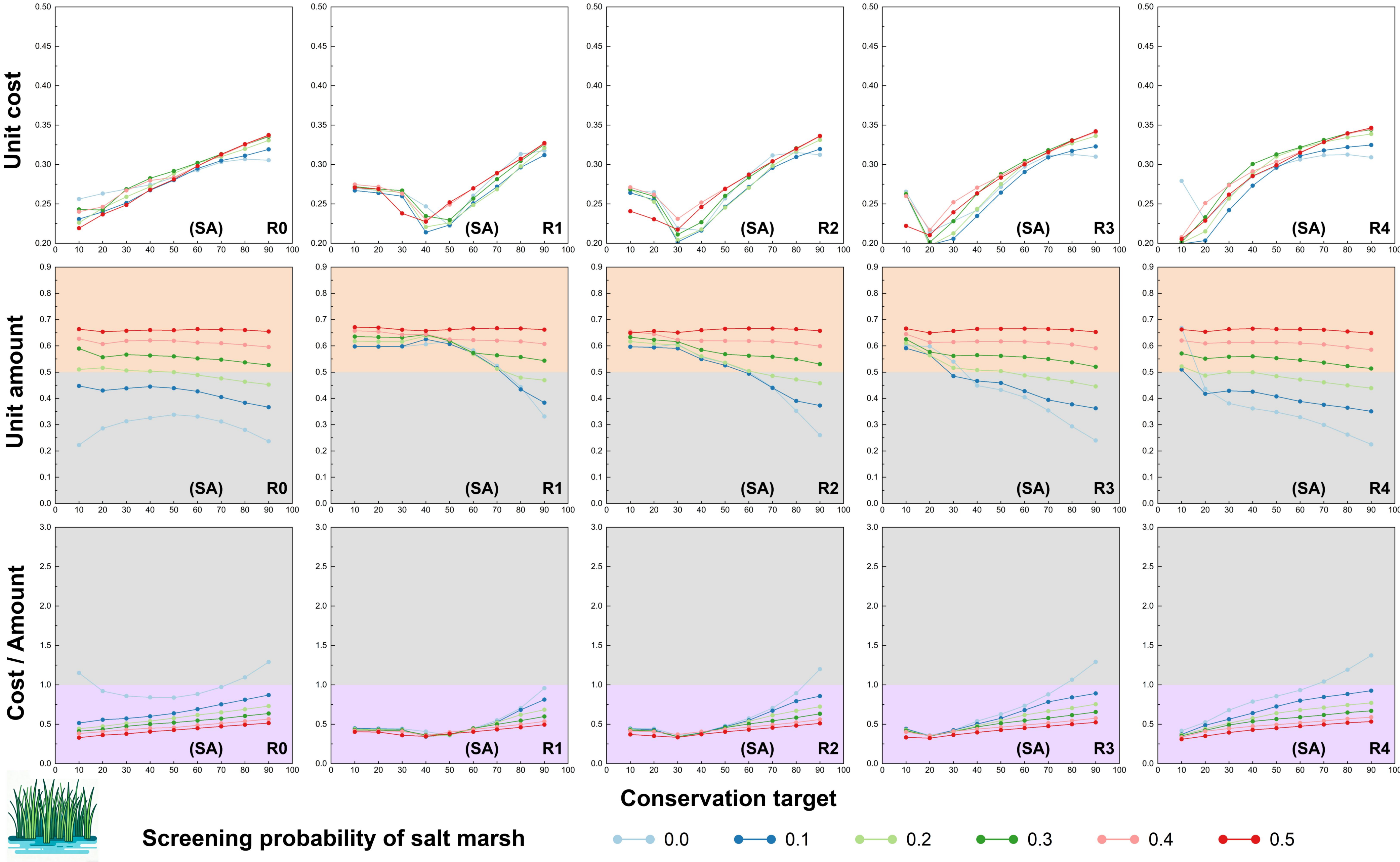

Supplementary Fig. S16 Comparison of unit amount, unit cost, and unit amount cost under different schemes and protection targets (salt marsh).

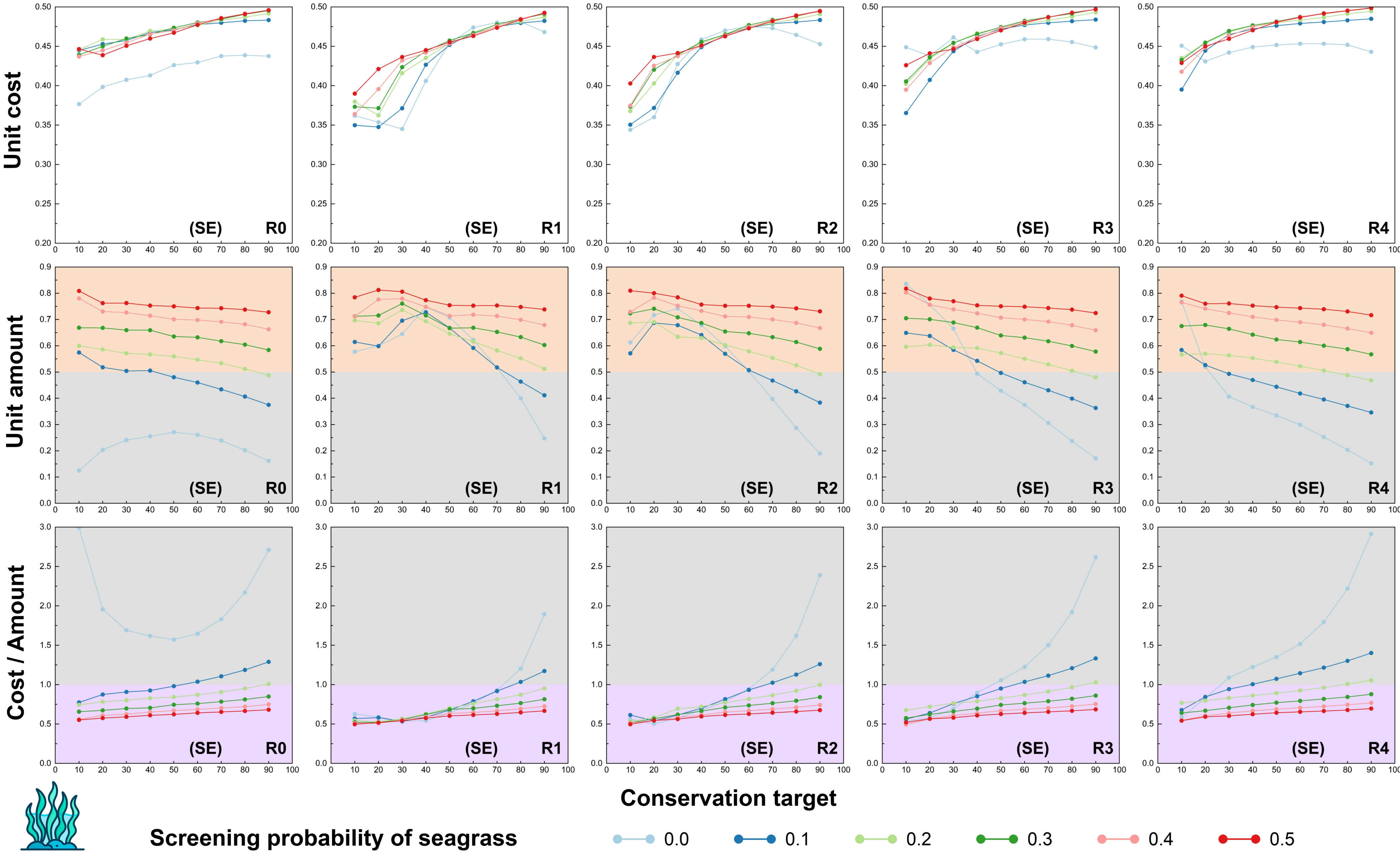

Supplementary Fig. S17 Comparison of unit amount, unit cost, and unit amount cost under different schemes and protection targets (seagrass).

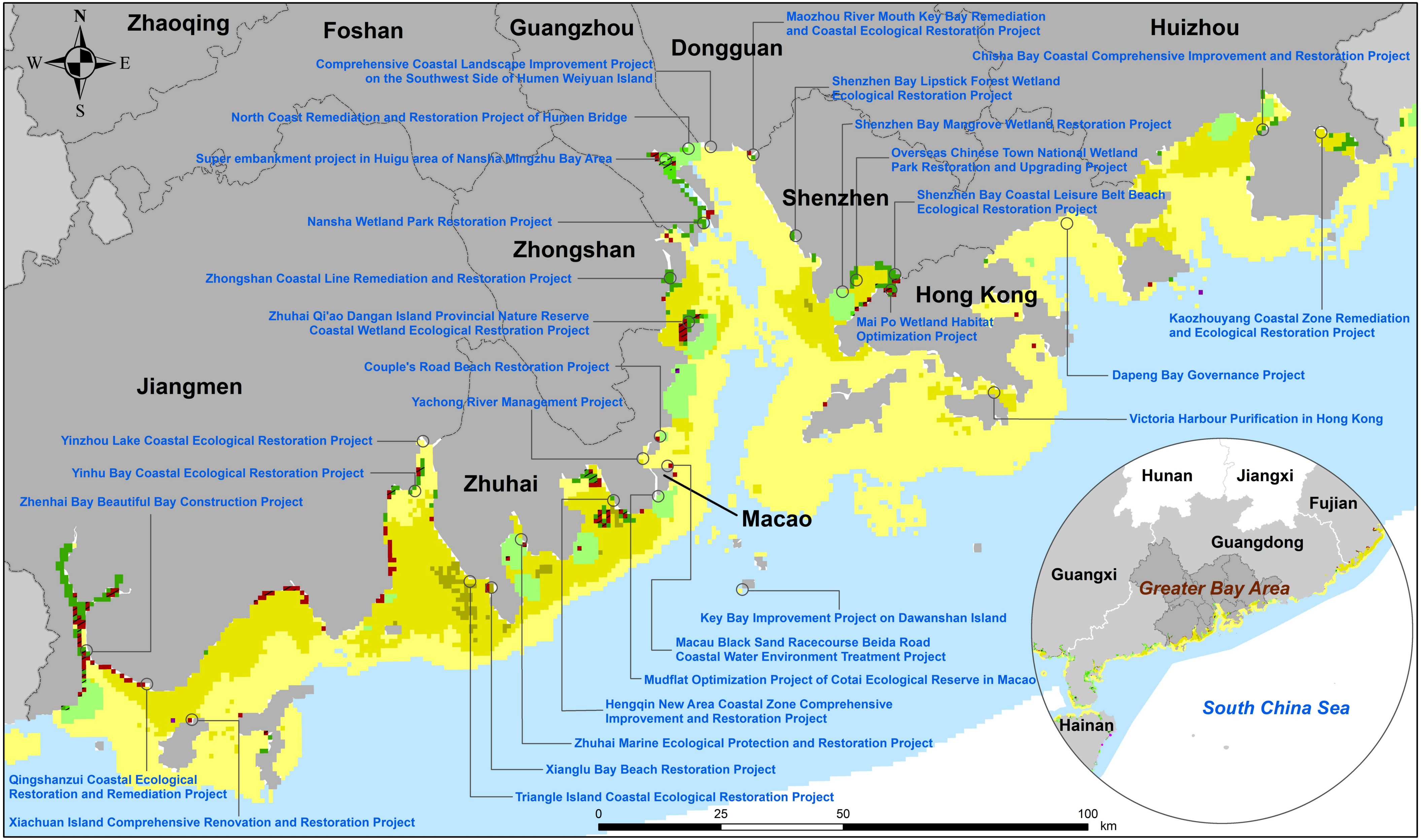

Supplementary Fig. S18 The launched protection and restoration projects in Greater Bay Area.

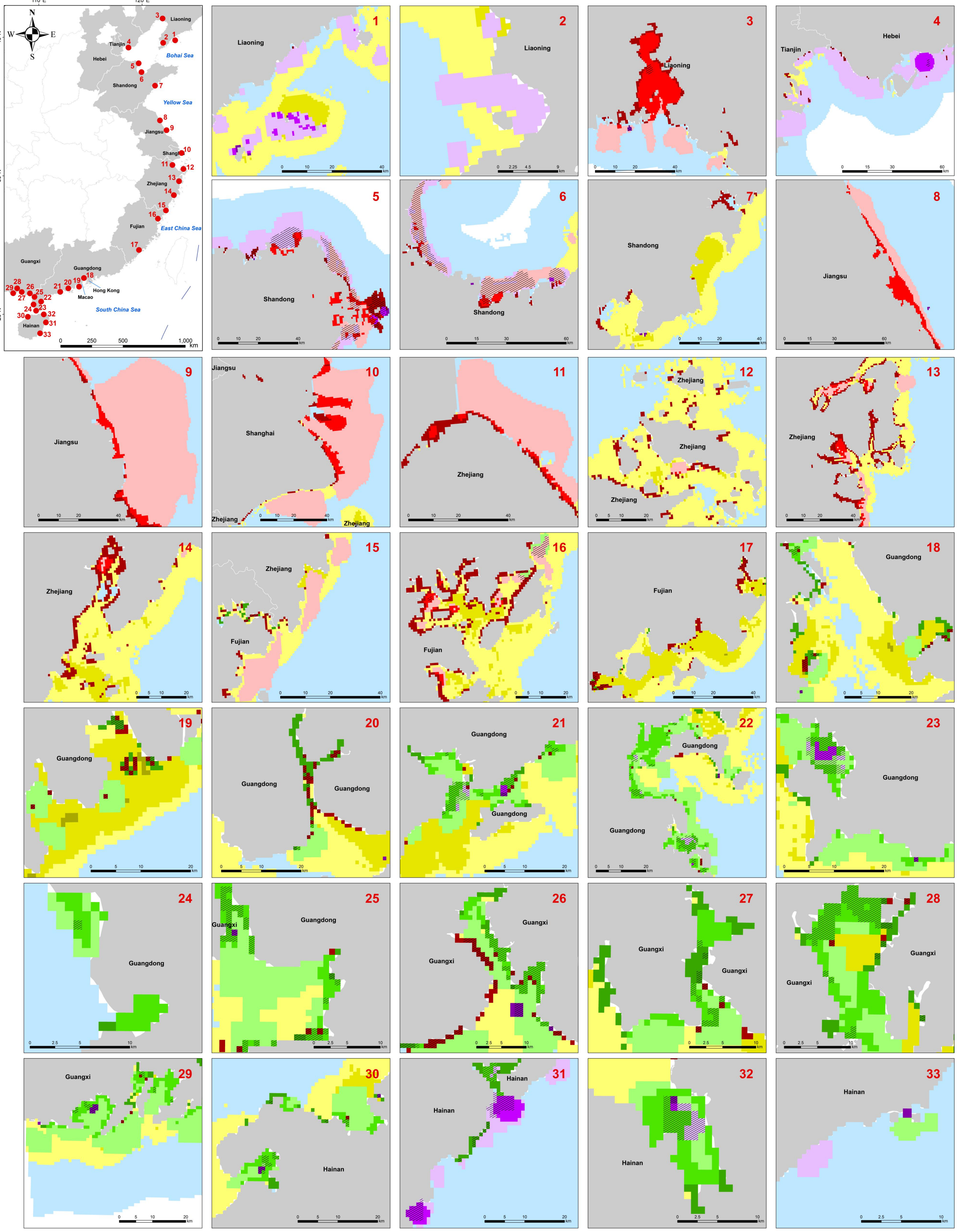

Supplementary Fig. S19 Localized enlargement of spatial protection planning strategies in different regions (1-33).

Supplementary Table S1. List of abbreviations and their full names used in the article.

Abbreviation	Full Name
AUC	Area under the curve
BLM	Boundary length modifier
CAR	Carbon accumulation rate
CBCEs	Coastal blue carbon ecosystems
ECP	Exist CBCEs and protected
ECU	Exist CBCEs and unprotected
MA	Mangrove
Marxan	A portmanteau of “marine” and the acronym SPEXAN, which stands for Spatially Explicit ANnealing
MaxEnt	Maximum entropy
NCP	Not exist CBCEs and protected
PSDAs	Potential suitable distribution areas
PU	Planning unit
R0	The best solution with the lowest cost among 100 iterations
R1	The solution with frequency range of 80-100 among 100 iterations
R2	The solution with frequency range of 60-100 among 100 iterations
R3	The solution with frequency range of 40-100 among 100 iterations
R4	The solution with frequency range of 20-100 among 100 iterations
ROC	Receiver operating characteristic
SA	Salt marsh
SCP	Systematic conservation planning
SE	Seagrass

Note: The abbreviations of variables related to environmental predictors datasets are shown in **Supplementary Table S4**.

Supplementary Table S2. List of seagrass distribution data.

Number	Province	Longitude	Latitude	Area (ha)	Number	Province	Longitude	Latitude	Area (ha)
1	Liaoning	121.608	40.823	120.32	65	Shandong	120.340	36.053	9.84
2	Liaoning	120.746	40.538	1070.87	66	Shandong	120.363	36.051	1.12
3	Liaoning	120.833	40.673	45.00	67	Shandong	120.358	36.042	1.01
4	Liaoning	120.610	40.400	21.76	68	Shandong	120.195	35.934	0.98
5	Liaoning	119.896	39.997	76.21	69	Shandong	120.169	35.898	1.12
6	Liaoning	122.298	40.467	100.00	70	Jiangsu	120.761	33.450	25.00
7	Liaoning	122.596	39.280	212.76	71	Jiangsu	119.209	34.750	25.00
8	Liaoning	122.672	39.268	23.37	72	Jiangsu	119.214	34.689	0.03
9	Liaoning	122.634	39.286	11.85	73	Jiangsu	119.445	34.659	0.02
10	Liaoning	122.549	39.288	236.10	74	Zhejiang	122.346	29.947	10.00
11	Liaoning	122.522	39.297	53.68	75	Fujian	119.821	25.578	7.02
12	Liaoning	122.622	39.265	169.52	76	Fujian	119.610	25.471	126.94
13	Liaoning	122.750	39.232	39.50	77	Fujian	118.864	25.112	2.69
14	Liaoning	122.698	39.228	122.27	78	Fujian	118.367	24.583	0.23
15	Liaoning	122.683	39.232	28.80	79	Fujian	117.436	23.917	59.42
16	Liaoning	122.672	39.241	55.04	80	Fujian	117.231	23.656	273.48
17	Liaoning	122.666	39.233	17.23	81	Guangdong	116.905	23.546	417.95
18	Liaoning	122.654	39.233	21.30	82	Guangdong	116.875	23.470	36.85
19	Liaoning	122.637	39.240	12.20	83	Guangdong	114.638	22.467	1.77
20	Liaoning	122.387	39.213	25.41	84	Guangdong	114.515	22.549	3.05
21	Liaoning	122.342	39.194	111.81	85	Guangdong	113.593	22.337	2.85
22	Liaoning	122.324	39.184	223.63	86	Guangdong	113.481	22.084	25.28
23	Liaoning	122.389	39.170	45.95	87	Guangdong	113.392	22.014	1.84
24	Liaoning	122.599	39.220	21.44	88	Guangdong	112.778	21.652	0.01
25	Liaoning	122.520	39.234	145.40	89	Guangdong	112.590	21.703	0.17
26	Liaoning	123.160	39.066	44.92	90	Guangdong	111.907	21.681	128.82
27	Liaoning	122.727	39.038	30.88	91	Guangdong	111.765	21.634	2.18
28	Liaoning	122.939	39.558	9.44	92	Guangdong	111.047	21.493	1.66
29	Liaoning	122.115	39.178	6.84	93	Guangdong	110.452	21.186	2.30
30	Liaoning	122.157	39.157	90.04	94	Guangdong	110.504	20.916	54.23
31	Liaoning	122.115	39.145	1.45	95	Guangdong	110.403	20.600	4.62
32	Liaoning	122.080	39.121	4.73	96	Guangdong	110.128	20.241	1.53

33	Liaoning	122.011	39.073	1.23	97	Guangdong	109.952	20.435	852.60
34	Liaoning	121.769	39.002	1.24	98	Guangxi	108.046	21.544	3.65
35	Liaoning	121.333	38.816	3.29	99	Guangxi	108.201	21.585	274.59
36	Hebei	118.696	39.095	9025.56	100	Guangxi	108.379	21.636	3.13
37	Hebei	119.560	39.905	45.00	101	Guangxi	108.547	21.657	16.53
38	Hebei	118.979	39.182	100.00	102	Guangxi	108.558	21.879	2.37
39	Tianjin	117.529	38.625	466.00	103	Guangxi	108.646	21.734	3.48
40	Shandong	119.279	37.758	1031.80	104	Guangxi	109.097	21.565	1.35
41	Shandong	119.215	37.723	800.00	105	Guangxi	109.095	21.019	0.42
42	Shandong	118.970	37.766	0.12	106	Guangxi	109.282	21.434	65.43
43	Shandong	118.609	37.998	0.50	107	Guangxi	109.622	21.521	278.75
44	Shandong	118.568	38.042	0.30	108	Guangxi	109.690	21.493	9.53
45	Shandong	118.734	37.396	2.00	109	Guangxi	109.768	21.497	6.24
46	Shandong	122.517	36.900	272.84	110	Hainan	110.568	20.014	64.11
47	Shandong	122.568	37.296	37.30	111	Hainan	110.854	19.526	3100.00
48	Shandong	122.567	37.260	37.26	112	Hainan	110.637	19.285	1600.00
49	Shandong	122.595	37.225	37.22	113	Hainan	110.383	18.688	27.91
50	Shandong	122.572	37.179	37.18	114	Hainan	110.055	18.413	240.00
51	Shandong	122.517	37.083	469.72	115	Hainan	109.984	18.402	175.00
52	Shandong	121.967	37.450	442.19	116	Hainan	109.679	18.266	13.08
53	Shandong	122.570	37.349	295.05	117	Hainan	109.484	18.216	4.31
54	Shandong	122.330	37.404	2.00	118	Hainan	109.948	19.948	99.69
55	Shandong	120.327	37.687	0.05	119	Hainan	110.093	19.975	0.50
56	Shandong	119.677	37.154	100.00	120	Hainan	109.814	19.956	27.42
57	Shandong	121.360	37.601	51.90	121	Hainan	109.587	19.988	12.04
58	Shandong	121.448	37.516	2.68	122	Hainan	109.550	19.921	8.04
59	Shandong	121.447	37.516	10.21	123	Hainan	109.267	19.744	102.87
60	Shandong	121.417	37.537	49.83	124	Hainan	108.650	18.967	3.72
61	Shandong	120.704	38.109	50.00	125	Hainan	108.719	18.510	46.09
62	Shandong	118.112	38.139	300.00	126	Hainan	110.575	19.149	2.95
63	Shandong	119.233	37.170	146.81	127	Hainan	112.342	16.828	1200.00
64	Shandong	120.316	36.061	1.90					

Supplementary Table S3. List of nature reserve selection.

Number	Name of nature reserve	Primary conservation objects	Province	Protection level
1	Xiamen Rare Marine Species	Chinese white dolphin (Sousa chinensis), egret, lancelet (Branchiostoma belcheri)	Fujian	National-level
2	Zhangjiang River Estuary Mangrove	Mangrove ecosystem	Fujian	National-level
3	Guanjingyang Larimichthys Crocea	Large yellow croaker (Larimichthys crocea) and its habitat	Fujian	Provincial-level
4	Changle Minjiang River Estuary Wetland	Coastal wetland and waterbirds	Fujian	Provincial-level
5	Daya Bay Fishery Resources	Pearl oyster, lobster, grouper, and other species, as well as coral reef ecosystem	Guangdong	Provincial-level
6	Guangdong Huidong Port Sea Turtle	Sea turtles and their nesting and breeding grounds	Guangdong	National-level
7	Guangdong Zhanjiang Mangrove	Mangrove ecosystem	Guangdong	National-level
8	Pearl River Estuary Chinese White Dolphin	Chinese white dolphin (Sousa chinensis) and its habitat	Guangdong	National-level
9	Zhanjiang Leizhou Bay Chinese White Dolphin	Chinese white dolphin (Sousa chinensis) and its habitat	Guangdong	Municipal-level
10	Guangdong Leizhou Rare Marine Organisms	White dolphins and other rare marine organisms, as well as their habitats	Guangdong	National-level
11	Guangdong Nan'ao Migratory Birds	Migratory birds and their habitat	Guangdong	Provincial-level
12	Guangdong Nanpeng Islands	Fishery germplasm resources, rare and endangered wildlife	Guangdong	National-level
13	Naozhou Island Coastal Resources	Abalone, lobster, pen shell and other marine fishery resources	Guangdong	Municipal-level
14	Guangdong Neilingding Island-Futian	Rhesus monkey, birds, mangrove wetland ecosystem	Guangdong	National-level
15	Zhanjiang Xuwen Wailuo Bay Horseshoe Crab	Horseshoe crab and its habitat	Guangdong	Municipal-level
16	Wushi Dengtujiao Coral	Coral reef and its ecosystem	Guangdong	Municipal-level
17	Xuwen Coral Reef	Coral reef ecosystem	Guangdong	National-level
18	Guangxi Shankou Mangrove	Mangrove ecosystem	Guangxi	National-level
19	Yingpan Port-Yingluo Port Dugong	Dugong, Chinese white dolphin, other rare aquatic animals, and seagrass beds	Guangxi	National-level
20	Weizhou Island Birds	Migratory birds and their habitat	Guangxi	Provincial-level
21	Wanning Dahuajiao	Rhesus monkey and its habitat	Hainan	Municipal-level
22	Danzhou White Butterfly Shell	White butterfly shell (Pteria penguin) and its coral reef ecosystem	Hainan	Provincial-level
23	Hainan Dongzhai Port	Mangrove ecosystem and rare waterbirds	Hainan	National-level
24	Hainan Wanning Dazhou Island	Edible-nest swiftlet and its habitat	Hainan	National-level
25	Hainan Tongguling	Tropical evergreen monsoon dwarf forest ecosystem	Hainan	National-level
26	Nanwan Monkey Island	Rhesus monkey and its habitat	Hainan	Provincial-level
27	Sanya River Birds	Chinese egret (Egretta eulophotes) and mangrove ecosystem	Hainan	Municipal-level
28	Sanya Coral Reef	Coral reef and its ecosystem	Hainan	National-level
29	Sanya Tielu Port Mangrove	Mangrove ecosystem, rare and endangered plants, as well as their habitats	Hainan	Municipal-level

30	Hainan Eucheuma	Eucheuma and its habitat	Hainan	Provincial-level
31	Danzhou Xinying Bay Mangrove	Mangrove ecosystem	Hainan	Municipal-level
32	Yalong Bay Qingmei Port Mangrove	Mangrove ecosystem	Hainan	Municipal-level
33	Hebei Nanda Port Wetland and Birds	Wetland ecosystem, rare and endangered birds	Hebei	Provincial-level
34	Jiangsu Yancheng Wetland Rare Birds	Red-crowned crane and other rare birds, as well as their habitat	Jiangsu	National-level
35	Liaoning Yalu River Estuary Coastal Wetland	Coastal wetland ecosystem, rare birds	Liaoning	National-level
36	Dalian Spotted Seal	Spotted seal (Phoca largha) and its habitat	Liaoning	National-level
37	Liaoning Shuangtai River Estuary	Red-crowned crane, Saunders's gull, other rare waterbirds, as well as their habitat	Liaoning	National-level
38	Liaoning Snake Island-Laotie Mountain	Red-crowned crane, hooded crane, as well as their habitat	Liaoning	National-level
39	Changhai Marine Precious Organisms	Sea cucumber (Apostichopus japonicus), abalone (Haliotis discus hannai), scallop (Chlamys farreri)	Liaoning	Provincial-level
40	Binzhou Shell Dike Island and Wetland	Shell dike islands, wetland, rare birds	Shandong	National-level
41	Shandong Yellow River Delta	Yellow River estuary wetland, rare and endangered birds	Shandong	National-level
42	Rongcheng Swan	Whooper swan and its habitat	Shandong	National-level
43	Sanggou Bay	Rare marine animals and their habitat	Shandong	Municipal-level
44	Shandong Changdao Islands	Eagles, falcons, other raptors, as well as migratory bird habitats	Shandong	National-level
45	Shanghai Chongming Dongtan Birds	Migratory birds and wetland ecosystem	Shanghai	National-level
46	Jinshan Three Islands Marine Ecology	Island ecosystem and forest	Shanghai	Provincial-level
47	Shanghai Jiuduansha Wetland	Oriental white stork, black-faced spoonbill, tundra swan, as well as their habitat	Shanghai	National-level
48	Zhejiang Xiangshan Jiushan Islands Marine Ecology	Large yellow croaker, birds, as well as their habitat	Zhejiang	National-level

Supplementary Table S4. Source and description of environmental predictors datasets.

Category	Predictor	Number	Description	Unit	Salt marsh	Mangrove	Seagrass	Source	Resolution	Time span	Representation
Sea surface temperature (SST)	SST1	1	Annual Mean Sea Surface Temperature	°C	○	○	○○	RSS GCOM-W1 AMSR2 Monthly Environmental Suite on 0.25 deg grid, Version 8.2, Remote Sensing Systems (https://www.remss.com)	0.25 degrees	2012-2024	Long-term marine environmental conditions
	SST2	2	Sea Surface Temperature Seasonality	/	○	○	○○				
	SST3	3	Max Sea Surface Temperature of Warmest Month	°C	○○	○○	○○				
	SST4	4	Min Sea Surface Temperature of Coldest Month	°C	○	○	○				
	SST5	5	Sea Surface Temperature Annual Range	°C	○○	○	○				
Bioclimate	BOI1	6	Annual Mean Temperature	°C	○	○		WorldClim2.1 (https://www.worldclim.org)	30 arc-seconds	1970-2000	Long-term climatic condition
	BOI2	7	Mean Diurnal Range	°C	○○	○○					
	BOI3	8	Isothermality	/	○○	○○					
	BOI4	9	Temperature Seasonality	/	○	○					
	BOI5	10	Max Temperature of Warmest Month	°C	○○	○○					
	BOI6	11	Min Temperature of Coldest Month	°C	○	○					
	BOI7	12	Temperature Annual Range	°C	○	○					
	BOI8	13	Mean Temperature of Wettest Quarter	°C	○○	○○					
	BOI9	14	Mean Temperature of Driest Quarter	°C	○	○					
	BOI10	15	Mean Temperature of Warmest Quarter	°C	○	○					
	BOI11	16	Mean Temperature of Coldest Quarter	°C	○	○					
	BOI12	17	Annual Precipitation	mm	○	○○					
	BOI13	18	Precipitation of Wettest Month	mm	○○	○					
	BOI14	19	Precipitation of Driest Month	mm	○	○					
	BOI15	20	Precipitation Seasonality	/	○○	○					
	BOI16	21	Precipitation of Wettest Quarter	mm	○	○					
	BOI17	22	Precipitation of Driest Quarter	mm	○	○○					
	BOI18	23	Precipitation of Warmest Quarter	mm	○	○					
	BOI19	24	Precipitation of Coldest Quarter	mm	○	○					
Soil	ESP	25	Exchangeable Sodium Percentage	%	○○	○	○	Harmonized World Soil Database v2.0 (https://www.fao.org/soils-portal/data-hub/soil-maps-and-databases/harmonized-world-s	1km	/	Stable condition
	ELEC	26	Electric Conductivity	dS/m	○○	○○	○○				
	GYPSUM	27	Gypsum Content	%	○	○	○○				
	TCARBON	28	Calcium Carbonate content	%	○	○	○				
	ALUM	29	Aluminium Saturation	%	○	○○	○○				

	BSAT	30	Base Saturation	%	○○	○	○○	oil-database-v20/)			
	TEB	31	Total Exchangeable Base	coml$_c$/kg	○○	○○	○○				
	CN	32	Carbon/Nitrogen Ratio	C/N	○○	○○	○○				
	TN	33	Total Nitrogen Content	g/kg	○	○○	○○				
	PH	34	pH In Water	-log(H^+)	○	○	○				
	ORGC	35	Organic Carbon Content	%	○	○	○				
	BULK	36	Bulk Density	g/cm^3	○○	○○	○○				
	CLAY	37	Clay Content	%	○○	○○	○○				
	SILT	38	Silt Content	%	○○	○	○○				
	SAND	39	Sand Content	%	○○	○○	○○				
	COARSE	40	Coarse Fragments	%	○○	○○	○○				
	CECS	41	Cation Exchange Capacity Soil	coml$_c$/kg	○○	○	○				
Offshore environment	CC	42	Annual Mean Chlorophyll Concentration	mg/m^3			○○	National Marine Science Data Center, National Science and Technology Resource Sharing Service Platform (https://mds.nmdis.org.cn)	500m	2007-2008	Best available marine environmental condition
	SSC	43	Annual Mean Suspended Sediment Concentration	mg/L			○○				
	AOT	44	Annual Mean Aerosol Optical Thickness	mg/m^3			○				
	WR	45	Annual Mean Water-leaving Radiance	W/sr m^2			○○				
	SWT	46	Annual Mean Sea Water Transparency	m			○				
	WDR	47	Annual Mean Water Diffuse Reflection	/			○				
Weather	WS	48	Annual Mean Wind Speed	m/s	○	○		WorldClim2.1 (https://www.worldclim.org)	30 arc-seconds	1970-2000	Long-term climatic condition
	WVP	49	Annual Mean Water Vapor Pressure	kPa	○○	○○					
	SR	50	Annual Mean Solar Radiation	kJ/m^2 day	○○	○○					
Terrain	ELEVATION	51	Elevation	m	○○	○○	○○	WorldClim2.1 (https://www.worldclim.org)	30 arc-seconds	1970-2000	Stable condition
	SLOPE	52	Slope	°	○○	○○	○○				
Sea surface salinity (SSS)	SSS1	53	Annual Mean Sea Surface Salinity	‰	○	○○	○	Ocean and Climate (http://www.ocean.iap.ac.cn/?navAnchor=home)	0.25 degrees	1993-2018	Long-term marine environmental conditions
	SSS2	54	Sea Surface Salinity Seasonality	/	○○	○○	○○				
	SSS3	55	Max Sea Surface Salinity	‰	○○	○○	○○				
	SSS4	56	Min Sea Surface Salinity	‰	○	○	○				
	SSS5	57	Sea Surface Salinity Annual Range	‰	○○	○○	○○				
Biotic interaction	SMII	58	Salt marsh interaction index	/		○○	○○	/	30 arc-seconds	2015-2020	/
	MII	59	Mangrove interaction index	/	○○		○○				
	SGII	60	Seagrass interaction index	/	○○	○○					

Note: ○ represents the applicability of the compiled variables in different CBCEs, ○○ represents the final variable set for each CBCEs.

Supplementary Table S5. Source, description, and processing of stressor dataset.

Category	Subcategories	Unit	Source	Processing	Resolution	Time span
Land-based	Population density	people	The LandScan Program at Oak Ridge National Laboratory (ORNL) (https://landscan.ornl.gov/about)	For each grid cell, a moving circular window with a 10 km radius was applied, centered on the cell. The total population within this window was aggregated to represent the coastal population exposure for that location.	30 arc-seconds	2000-2024
	Light pollution	nW/sr cm²	Yangtze River Delta Science Data Center, National Earth System Science Data Sharing Infrastructure, National Science & Technology Infrastructure of China (http://geodata.nnu.edu.cn/data/datadetails.html?dataguid=8213124601985&docid=0)	A 10 km radius circular moving window was centered on each grid cell, and the total night-time light intensity within the window was summed to represent local artificial light exposure.	500m	2000-2024
	Port activities	/	National Coastal Port Layout Plan (https://www.gov.cn)	Port activities were interpolated into a continuous raster surface using Inverse Distance Weighting, which assigns values to unmeasured locations based on the proximity and value of known port points.	/	2000-2024
Ocean	Mariculture	/	National Earth System Science Data Center, National Science & Technology Infrastructure of China (https://www.geodata.cn/data/datadetails.html?dataguid=122606915581534&docId=0)	A 10 km influence radius was applied around each mariculture point. The total number of mariculture units within each 10 km grid zone was summed to quantify mariculture intensity.	/	2020
	Bulk shipping	/	Ship News Network (https://www.shipxy.com/)	Shipping intensity was quantified by counting the number of vessel transits passing through each grid cell.	/	2000-2024
	Cruise shipping	/				
	Container shipping	/				
Fishing	Commercial demersal destructive	hours	Global Fishing Watch (https://globalfishingwatch.org/)	For each vessel, the total annual fishing hours were calculated by summing all fishing activity records. These values were then aggregated by fishing category to produce total fishing effort per grid cell.	1km	2012-2024
	Commercial demersal nondestructive high bycatch	hours				
	Commercial demersal nondestructive low bycatch	hours				
	Pelagic high bycatch	hours				
	Pelagic low bycatch	hours				
	Artisanal	hours				

Supplementary Table S6. List of gear types classified into each of the 5 types of commercial fishing mapped.

Gear type	Bycatch	Type	Destruction
Dredge fishing	/	demersal	destructive
Drifting longlines	high	pelagic	/
Fixed gear	high	demersal	nondestructive
Purse seines	low	pelagic	/
Seines	high	demersal	nondestructive
Pole and line	low	pelagic	/
Pots and traps	low	demersal	nondestructive
Set gillnets	high	demersal	nondestructive
Set longlines	high	pelagic	/
Squid jigger	low	pelagic	/
Trawlers	/	demersal	destructive
Trollers	low	pelagic	/
Tune purse seines	low	pelagic	/
Other purse seines	low	pelagic	/

Supplementary Table S7. Anthropogenic pressure influence weight matrix.

Stressor	Subcategories	Original weight			Normalized weight		
		Mangrove	Salt marsh	Seagrass	Mangrove	Salt marsh	Seagrass
Land-based	Population density	3.30	1.60	2.50	0.24	0.14	0.20
	Light pollution	0.90	1.80	0.50	0.06	0.15	0.04
	Port activities	1.00	1.20	2.80	0.07	0.10	0.22
Ocean	Mariculture	3.30	1.60	2.50	0.24	0.14	0.20
	Bulk shipping	0.67	0.47	0.63	0.05	0.04	0.05
	Cruise shipping	0.67	0.47	0.63	0.05	0.04	0.05
	Container shipping	0.67	0.47	0.63	0.05	0.04	0.05
Commercial fishing	Demersal destructive	0.00	1.00	0.20	0.00	0.08	0.02
	Demersal nondestructive high bycatch	0.90	1.00	1.10	0.06	0.08	0.09
	Demersal nondestructive low bycatch	0.80	0.70	0.70	0.06	0.06	0.06
	Pelagic high bycatch	0.00	0.50	0.00	0.00	0.04	0.00
	Pelagic low bycatch	0.00	0.40	0.00	0.00	0.03	0.00
	Artisanal fishing	1.70	0.60	0.30	0.12	0.05	0.02

Note: Refer to the research of Halpern et al. (2008, 2019) [1,2].

Supplementary Table S8. Optimal BLM estimation results.

Target (%)		10		20		30		40		50		60		70		80		90	
SP	Category	Mid	SR	Mid	SR	Mid	SR	Mid	SR	Mid	SR	Mid	SR	Mid	SR	Mid	SR	Mid	SR
0.0	Mangrove	0.43	0.15-0.51	0.43	0.17-0.51	0.43	0.19-0.52	0.43	0.21-0.52	0.45	0.24-0.54	0.45	0.28-0.53	0.48	0.32-0.56	0.53	0.37-0.61	0.65	0.46-0.75
0.0	Salt marsh	0.41	0.14-0.51	0.42	0.17-0.56	0.41	0.19-0.58	0.41	0.23-0.58	0.42	0.27-0.56	0.47	0.33-0.62	0.58	0.41-0.82	0.78	0.57-1.13	1.16	0.89-1.82
0.0	Seagrass	0.58	0.22-0.70	0.58	0.24-0.71	0.58	0.27-0.72	0.58	0.30-0.72	0.61	0.35-0.74	0.64	0.41-0.77	0.72	0.49-0.89	0.91	0.6-1.23	1.31	0.91-1.87
0.1	Mangrove	1.15	0.55-9.31	6.27	0.15-1.74	3.30	0.21-3.55	9.90	0.27-3.04	6.17	0.19-5.25	5.66	0.22-6.44	6.69	0.20-15.45	8.80	0.4-37.23	21.21	0.26-83.94
0.1	Salt marsh	0.60	0.19-0.80	0.60	0.25-1.86	0.58	0.30-3.81	0.64	0.38-1.26	0.77	0.48-0.85	1.11	0.39-0.62	1.82	0.30-0.86	3.11	0.36-1.35	4.78	0.35-2.53
0.1	Seagrass	1.29	0.64-5.01	1.70	0.37-0.73	2.14	0.33-0.83	2.49	0.35-0.99	3.47	0.26-1.31	4.82	0.26-1.71	5.52	0.22-2.28	5.39	0.15-2.40	3.79	0.30-2.49
0.2	Mangrove	0.57	0.55-0.60	1.85	0.20-2.61	4.41	0.11-2.78	5.43	0.17-4.58	4.05	0.16-7.47	5.21	0.28-55.94	8.80	0.24-298.81	45.41	0.03-60.44	12.15	0.20-8.30
0.2	Salt marsh	0.69	0.21-1.05	0.71	0.30-2.08	0.79	0.20-0.38	0.91	0.2-0.45	1.29	0.11-0.62	2.01	0.07-0.83	2.84	0.08-1.17	3.87	0.07-1.73	3.23	0.05-1.89
0.2	Seagrass	52.73	0.32-1.33	8.29	0.05-1.44	23.10	0.07-1.86	11.47	0.05-1.89	12.73	0.07-2.71	11.36	0.01-1.96	6.98	0.15-3.18	5.35	0.11-3.03	3.54	0.18-2.92
0.3	Mangrove	0.16	0.11-0.21	1.09	0.25-4.87	3.07	0.21-9.33	5.66	0.19-16.95	6.70	0.22-65.39	9.04	0.11-19.85	16.11	0.52-45.4	14.02	0.05-6.80	4.03	0.29-3.67
0.3	Salt marsh	1.09	0.33-2.13	1.23	0.13-0.38	1.55	0.01-0.48	1.89	0.02-0.58	2.77	0.03-0.84	3.52	0.04-1.16	2.83	0.01-1.30	2.22	0.02-1.35	1.78	0.09-1.39
0.3	Seagrass	4.81	0.57-2.26	3.89	0.07-2.35	4.81	0.01-3.06	10.48	0.14-6.46	14.89	0.32-16.35	14.04	0.24-5.12	6.37	0.27-3.53	3.35	0.18-2.57	2.49	0.34-2.30
0.4	Mangrove	0.10	0.05-0.14	0.70	0.19-1.54	2.00	0.07-4.99	9.09	0.26-21.97	23.91	0.00-14.94	9.12	0.34-7.49	5.46	0.40-4.74	3.53	0.55-3.14	2.37	0.59-2.27
0.4	Salt marsh	0.66	0.18-2.06	4.55	0.00-0.70	69.65	0.08-1.85	32.03	0.14-1.81	7.41	0.02-1.94	3.69	0.01-1.50	2.14	0.03-1.25	1.53	0.04-1.17	1.21	0.06-1.08
0.4	Seagrass	1.25	0.03-2.87	2.29	0.01-4.83	3.94	0.02-8.12	6.13	0.01-12.27	53.68	0.1-365.96	8.18	0.21-4.84	4.30	0.23-3.31	2.80	0.28-2.40	1.93	0.23-1.84
0.5	Mangrove	0.06	0.03-0.09	0.42	0.38-0.57	0.99	0.18-1.69	3.12	0.11-4.64	15.09	0.72-12.97	3.94	1.25-3.33	2.31	2.29-21.4	1.92	1.86-2.59	1.67	1.62-1.78
0.5	Salt marsh	0.12	0.05-0.18	0.67	0.00-0.66	1.64	0.06-0.64	4.36	0.09-2.17	12.27	0.07-2.93	2.89	0.00-1.52	1.67	0.02-1.15	1.19	0.08-0.99	1.03	0.10-0.95
0.5	Seagrass	1.87	0.01-2.80	7.52	0.08-8.29	2.99	0.06-41.25	8.47	0.08-48.61	25.91	0.55-87.3	8.10	0.20-6.85	3.78	0.15-3.33	2.35	0.32-2.16	1.86	0.21-1.77

Note: SP represents screening probability, Mid represents midpoint, SR represents sensitive range.

Supplementary Table S9. Planning unit, cost, and amount under different schemes and protection targets (screening probability 0.0).

Category	Target	Total			Planning unit					Cost					Amount				
	(%)	PU	Cost	Amount	R0	R1	R2	R3	R4	R0	R1	R2	R3	R4	R0	R1	R2	R3	R4
	10				17448	406	467	697	1764	4756.06	198.42	227.35	331.38	797.68	1383.67	283.16	326.19	485.69	1224.29
	20				20218	474	614	1561	10604	6053.60	230.62	293.99	674.72	4358.31	2766.89	331.21	426.65	1057.16	5417.19
	30				24746	528	1061	6762	21411	7764.82	255.80	466.06	2929.23	9051.89	4150.81	369.33	713.39	3984.87	9055.96
	40				28511	653	4150	14279	27819	9370.37	305.93	1806.12	6065.16	12034.71	5534.41	449.04	2563.55	7035.74	10774.30
Mangrove	50	398362	113760.23	13835.94	33152	2092	9706	20504	31661	11373.79	927.75	4380.20	8956.12	13737.33	6917.27	1369.89	5494.68	9128.54	11529.00
	60				36794	6748	15667	25814	34836	13093.16	3090.03	7160.53	11421.58	15115.72	8301.36	3974.39	7910.21	10491.10	12023.48
	70				43752	12736	22973	31955	39859	15797.18	5906.63	10460.83	14077.02	17111.94	9685.18	6604.72	9928.55	11616.40	12551.27
	80				51156	21609	32104	39415	45527	18776.56	9949.40	14184.65	16920.40	19230.52	11068.89	9410.00	11542.70	12470.46	12955.34
	90				63428	36132	44256	48367	53582	23380.90	15951.99	18688.49	20175.73	22080.88	12452.58	12045.94	12749.07	13097.53	13324.19
	10				22185	1344	1484	1764	6191	5684.05	362.41	396.21	468.28	1728.07	4938.15	803.60	884.45	1059.59	4137.76
	20				34551	1498	1760	5935	45562	9094.35	399.98	466.28	1273.61	10437.61	9878.07	895.00	1049.20	3549.12	19872.80
	30				47343	1604	4300	24735	83624	12728.39	424.69	942.44	5643.86	21640.57	14816.64	957.46	2609.38	13362.16	31856.11
	40				60617	2164	18957	52463	106613	16617.87	534.26	4102.20	12711.34	30376.63	19755.59	1312.02	10558.21	23552.20	38559.57
Salt marsh	50	398362	110563.76	49389.75	73026	10815	35120	72121	120129	20708.82	2401.68	9028.25	19589.30	35767.82	24695.32	6688.07	18796.63	31195.78	41795.74
	60				89461	24891	53532	91757	134582	26186.43	6485.65	15399.01	27285.76	41226.74	29633.82	14505.98	26804.74	37128.50	44194.43
	70				110842	42388	78334	120162	154446	33599.92	12222.00	24409.26	37421.86	48167.85	34572.75	22217.27	34591.43	42560.91	46240.20
	80				140998	70166	119277	157064	181862	43274.34	21980.76	37605.85	49151.44	56859.33	39513.09	31137.51	42060.73	46137.58	47718.71
	90				187741	125231	180754	199793	215471	57344.50	39765.02	56455.10	61939.27	66598.03	44450.79	41498.38	47047.20	47956.21	48485.26
	10				18415	65	80	612	3830	6935.71	23.52	27.52	274.69	1726.09	2295.99	37.52	48.98	511.58	2941.83
	20				22547	80	189	4231	18294	8982.81	28.29	68.03	1851.52	7881.96	4591.79	47.98	135.47	3198.25	9510.15
	30				28577	109	3212	11277	35050	11643.90	37.61	1373.15	5201.59	15492.35	6889.04	70.22	2382.87	7506.80	14235.25
	40				35964	1321	9237	23472	44444	14854.72	536.39	4235.93	10392.78	19956.55	9184.50	987.65	6266.67	11583.46	16315.95
Seagrass	50	398362	154311.42	22963.01	42365	6620	16409	33325	52654	18052.73	3007.43	7713.30	15077.62	23783.55	11482.05	4684.29	9816.35	14281.93	17621.51
	60				52822	13797	25669	43689	62816	22690.89	6538.27	12198.56	20051.35	28470.45	13777.78	8588.83	12912.92	16382.52	18794.36
	70				67208	23266	40150	60634	79576	29412.04	11172.61	18989.83	27837.69	36068.11	16075.97	12011.28	15948.89	18549.07	20082.57
	80				90843	38914	65872	86106	104384	39850.55	18724.51	30596.28	39210.92	47166.72	18370.37	15562.61	18892.21	20420.22	21250.88
	90				127970	79766	112114	127526	146088	55985.99	37335.29	50756.76	57208.83	64715.74	20666.58	19719.19	21250.08	21856.23	22212.53

Supplementary Table S10. Planning unit, cost, and amount under different schemes and protection targets (screening probability 0.1).

Category	Target	Total			Planning unit					Cost					Amount				
	(%)	PU	Cost	Amount	R0	R1	R2	R3	R4	R0	R1	R2	R3	R4	R0	R1	R2	R3	R4
Mangrove	10	35080	16107.25	12343.98	2326	599	696	896	2176	974.72	290.08	334.81	412.12	840.26	1234.40	416.64	482.37	594.67	1146.31
	20				5662	657	884	3632	11623	2566.24	317.57	410.40	1526.78	5308.75	2468.79	450.81	576.37	1603.21	4646.97
	30				8821	814	3442	9530	16900	4011.05	392.14	1536.96	4387.23	7885.30	3703.27	545.43	1630.24	3918.03	6875.78
	40				11125	1338	6782	13812	20599	5229.72	610.09	3103.47	6414.68	9594.13	4937.61	822.60	3193.26	6009.70	8582.69
	50				14346	4503	11422	17189	22732	6706.80	2068.92	5312.89	8005.91	10573.32	6172.06	2407.33	5294.70	7502.89	9430.32
	60				17507	9247	15410	20164	25008	8125.84	4342.69	7200.79	9405.64	11627.27	7406.39	4542.14	6910.20	8640.83	10171.70
	70				20969	14689	19503	23201	27253	9772.24	6894.56	9123.01	10829.53	12648.54	8640.82	6624.32	8391.93	9603.08	10770.00
	80				24580	19905	23749	26726	29809	11451.77	9328.74	11094.80	12426.00	13778.66	9875.19	8505.89	9763.27	10624.19	11380.52
	90				29053	25894	28718	30770	32555	13455.55	12074.88	13327.18	14212.48	14984.05	11109.61	10387.27	11132.32	11589.34	11934.47
Salt marsh	10	129736	42250.16	44657.11	9979	1515	1709	1994	10642	2302.90	404.54	451.30	521.66	2015.38	4465.66	905.62	1019.94	1178.60	5426.61
	20				20762	1659	2090	10622	41790	4982.75	437.82	533.33	2102.04	8505.48	8931.22	990.47	1241.06	6003.28	17455.18
	30				30563	1864	9163	28366	59361	7679.29	484.18	1848.47	5838.83	14366.45	13396.99	1115.38	5410.22	13755.98	25461.33
	40				40141	4865	20593	44678	74644	10732.27	1040.76	4452.13	10487.22	20389.68	17862.54	3041.69	11315.72	20839.32	31770.06
	50				50869	13591	33525	58906	88915	14254.29	3036.30	8260.23	15574.65	26314.37	22328.27	8251.76	17629.67	27030.13	36247.81
	60				62801	24266	48259	77097	101540	18518.11	6085.37	13099.14	22398.53	31566.95	26794.29	13883.28	23842.65	32959.53	39459.69
	70				77139	39616	70034	95847	110255	23522.44	10770.76	20716.09	29614.29	35048.94	31259.95	20403.73	30814.64	37809.42	41437.48
	80				93177	66168	94852	107691	117435	28996.75	19608.89	29361.30	34171.30	37821.49	35725.58	28735.15	37040.29	40646.96	42815.32
	90				109622	97739	110818	118305	125733	34995.85	30486.05	35420.49	38200.61	40837.06	40192.37	37508.03	41325.22	42862.49	44104.47
Seagrass	10	56392	27417.05	18722.63	3260	90	112	305	5307	1449.95	31.48	39.25	111.41	2096.82	1872.32	55.31	63.96	197.95	3099.17
	20				7231	103	535	4375	14988	3274.27	35.80	198.90	1781.89	6661.78	3744.59	61.63	367.65	2788.86	7892.92
	30				11143	214	3471	10654	22476	5096.68	79.46	1445.50	4727.62	10446.69	5617.05	148.83	2354.87	6224.73	11081.71
	40				14819	2492	8061	16861	27281	6929.10	1062.97	3618.87	7797.82	12874.45	7489.04	1813.85	5167.09	9146.17	12811.09
	50				19494	6479	14219	22607	31479	9179.98	2926.93	6606.64	10668.32	14988.45	9361.47	4326.51	8096.39	11223.80	13964.96
	60				24420	12209	20721	28055	36062	11658.26	5696.16	9808.82	13381.79	17274.35	11233.57	7222.65	10516.45	12931.56	15086.02
	70				30203	19647	27176	33652	40673	14492.72	9333.88	13008.94	16147.62	19555.74	13105.93	10168.72	12701.51	14490.36	16089.42
	80				36840	27881	34643	40291	46416	17771.43	13369.11	16655.79	19417.14	22415.27	14978.17	12925.33	14774.88	16067.43	17221.19
	90				44942	38097	43970	48576	52722	21719.03	18372.32	21251.95	23506.30	25562.51	16850.27	15673.82	16861.99	17633.49	18242.23

Supplementary Table S11. Planning unit, cost, and amount under different schemes and protection targets (screening probability 0.2).

Category	Target (%)	Total PU	Total Cost	Total Amount	Planning unit R0	Planning unit R1	Planning unit R2	Planning unit R3	Planning unit R4	Cost R0	Cost R1	Cost R2	Cost R3	Cost R4	Amount R0	Amount R1	Amount R2	Amount R3	Amount R4
Mangrove	10	25213	11940.85	10917.31	1632	620	730	961	1850	746.75	298.43	349.01	439.60	797.73	1091.69	434.56	511.12	660.24	1258.46
	20				4033	710	919	2068	7245	1856.77	343.78	432.53	919.05	3363.13	2183.46	491.76	615.00	1245.23	3996.60
	30				6375	883	2428	6371	11694	2974.72	422.22	1117.46	3018.98	5573.54	3275.27	593.82	1416.66	3425.17	5945.18
	40				8570	2211	5844	9788	14119	4089.67	1035.11	2790.48	4694.63	6718.09	4367.03	1330.19	3192.12	5100.70	7001.86
	50				10841	4829	8931	12399	16410	5157.72	2291.88	4278.09	5918.44	7767.76	5458.67	2729.12	4729.21	6301.18	8009.00
	60				13331	8669	11859	14781	17953	6358.92	4174.82	5679.81	7032.86	8495.74	6550.39	4581.72	6028.38	7295.13	8570.84
	70				16011	12013	14951	17300	19911	7599.89	5775.94	7126.12	8209.97	9418.35	7642.22	6056.94	7319.62	8279.61	9265.84
	80				18758	15983	18241	19928	21561	8925.92	7610.09	8665.10	9449.81	10216.01	8733.90	7674.36	8578.39	9233.20	9832.40
	90				21587	19734	21538	22599	23691	10226.78	9361.28	10200.47	10702.91	11216.35	9825.62	9222.85	9852.62	10197.09	10518.12
Salt marsh	10	89062	30255.28	38833.05	7609	1443	1647	1955	10592	1721.17	391.15	442.03	514.44	2017.07	3883.29	891.66	1015.24	1194.90	5525.98
	20				15037	1628	2138	11014	30925	3679.09	434.95	540.30	2169.10	6653.52	7766.76	1003.17	1301.17	6212.32	15055.88
	30				23015	1807	8225	24830	43150	5960.37	477.80	1680.89	5280.79	11082.69	11650.06	1113.20	4958.34	12822.19	21581.20
	40				30863	4284	19074	36434	53657	8390.14	945.72	4151.00	8879.31	15438.16	15533.24	2733.01	10685.03	18502.07	26801.32
	50				38843	11723	30112	46640	63201	11212.05	2650.54	7389.54	12835.46	19605.10	19416.68	7298.43	16141.71	23516.36	30625.34
	60				47640	23649	42743	56793	69776	14389.57	5878.16	11559.74	17097.74	22391.62	23300.06	13440.76	21555.96	27683.10	32916.66
	70				57082	38431	54499	65435	75718	17690.69	10321.69	16259.18	20700.13	24852.58	27183.27	19718.48	26480.57	31086.00	34922.62
	80				67009	53790	65441	73719	82136	21430.12	16011.24	20731.54	24097.40	27469.95	31066.60	25766.22	30894.86	34140.68	36916.21
	90				77209	68245	77473	84013	87532	25530.04	21914.61	25661.25	28254.03	29654.65	34949.87	32018.30	35460.18	37448.74	38445.11
Seagrass	10	34233	16987.61	15577.62	2597	58	82	1610	5349	1157.90	22.02	30.15	647.63	2327.88	1557.80	40.40	56.34	959.40	3030.19
	20				5319	79	845	4934	10646	2440.41	28.63	340.44	2141.52	4832.81	3115.61	54.16	583.51	2977.32	6067.06
	30				8178	821	4543	8807	14665	3750.52	341.49	1998.02	3998.34	6862.99	4673.37	604.45	2880.35	5230.06	8263.20
	40				10991	2999	7487	12461	18130	5160.25	1305.23	3396.49	5815.03	8637.05	6231.08	2079.43	4712.19	7367.23	10027.50
	50				13919	6910	11358	15581	20309	6569.19	3135.60	5288.25	7385.41	9749.93	7788.88	4462.63	6852.01	8913.00	10937.14
	60				17093	10873	15042	18765	22882	8145.41	5076.25	7126.58	8977.01	11054.55	9346.57	6697.12	8704.10	10328.83	11947.39
	70				20443	15103	18939	22304	25809	9879.94	7172.16	9080.90	10769.57	12561.86	10904.34	8795.81	10491.45	11798.31	13048.32
	80				24347	19647	23278	26246	28995	11857.64	9455.56	11279.77	12800.14	14244.22	12462.09	10850.32	12235.26	13247.16	14139.68
	90				28736	25532	28435	30352	32210	14128.96	12447.05	13957.75	14964.89	15925.56	14019.86	13079.06	13991.35	14565.55	15077.43

Supplementary Table S12. Planning unit, cost, and amount under different schemes and protection targets (screening probability 0.3).

Category	Target	Total			Planning unit					Cost					Amount				
	(%)	PU	Cost	Amount	R0	R1	R2	R3	R4	R0	R1	R2	R3	R4	R0	R1	R2	R3	R4
Mangrove	10	17561	8374.30	8999.14	1273	544	715	1077	1947	562.45	253.03	320.51	464.23	812.40	899.89	381.86	501.77	761.75	1363.58
	20				2862	766	978	1762	4534	1317.78	368.20	455.62	802.23	2079.18	1799.81	534.80	674.23	1184.30	2923.12
	30				4596	967	2168	4459	7935	2210.61	462.02	1029.48	2133.72	3800.03	2699.78	656.83	1391.53	2732.02	4602.67
	40				6311	2417	4578	6848	9832	3047.48	1167.37	2213.28	3305.34	4712.09	3599.71	1535.96	2755.78	3984.54	5502.69
	50				7935	4510	6739	8843	11320	3827.80	2187.00	3262.63	4261.35	5417.67	4499.57	2699.53	3914.21	4986.04	6225.20
	60				9781	6813	8958	10777	12533	4736.46	3308.63	4318.25	5173.75	5993.57	5399.56	3928.10	5024.52	5940.05	6808.07
	70				11561	9276	11040	12399	13785	5522.86	4483.53	5299.26	5937.18	6591.86	6299.42	5166.95	6053.37	6731.63	7413.56
	80				13358	11581	13017	14167	15293	6379.79	5549.78	6230.84	6762.16	7279.84	7199.46	6329.41	7043.33	7611.26	8133.33
	90				15252	14155	15164	15853	16631	7263.79	6749.70	7220.83	7544.43	7922.44	8099.25	7631.86	8096.23	8378.52	8678.53
Salt marsh	10	63736	22045.66	32561.15	5521	1363	1554	1872	8958	1341.93	370.82	417.11	490.87	1805.40	3256.17	865.99	984.17	1170.28	5114.97
	20				11697	1548	1945	9579	22771	2835.07	416.26	505.16	1929.07	5307.55	6512.26	980.50	1212.06	5527.09	12554.83
	30				17235	1718	6213	20236	31575	4630.09	458.62	1312.29	4616.35	8658.85	9768.43	1085.36	3827.29	11370.68	17631.84
	40				23120	3048	16733	27586	38957	6532.58	715.04	3795.10	7257.58	11711.55	13024.52	1960.25	9783.68	15581.12	21821.61
	50				29068	12442	25602	33790	43399	8481.10	2857.15	6662.76	9723.65	13578.03	16280.69	7695.53	14554.90	18984.47	24009.81
	60				35373	23700	31973	39294	47606	10685.35	6085.94	9065.30	11971.26	15318.32	19536.72	13582.98	17986.55	21900.94	25978.87
	70				41621	30981	38680	45337	52878	13035.10	8718.83	11749.30	14424.86	17505.94	22792.90	17476.84	21609.76	24938.71	28361.31
	80				48499	38901	46282	52494	58740	15760.40	11845.85	14837.99	17363.88	19947.48	26049.11	21685.08	25408.11	28205.77	30746.11
	90				55609	49073	55616	59952	62610	18651.63	15973.37	18670.04	20461.69	21556.15	29305.06	26684.01	29502.63	31204.12	32173.52
Seagrass	10	23003	11509.22	12817.72	1917	64	83	916	3611	841.79	23.88	30.99	371.37	1562.32	1281.90	45.59	60.02	645.65	2437.49
	20				3837	78	1205	3518	7052	1726.58	28.97	506.45	1533.77	3206.53	2563.55	55.79	892.77	2465.37	4790.16
	30				5831	1149	3499	6213	9697	2681.76	486.56	1535.99	2823.08	4551.83	3845.32	873.94	2478.33	4276.98	6443.01
	40				7776	3542	6135	8555	11706	3615.89	1572.41	2795.92	3982.08	5578.57	5127.14	2533.70	4212.70	5723.63	7518.74
	50				10086	6721	8779	10922	13189	4775.43	3073.16	4081.05	5180.65	6347.13	6408.96	4482.83	5744.42	6982.49	8227.87
	60				12169	8566	11092	13228	15602	5844.29	3999.96	5289.63	6379.42	7606.76	7690.66	5725.99	7184.60	8345.85	9583.98
	70				14532	11233	13587	15556	17685	7040.98	5366.98	6569.62	7579.27	8689.33	8972.44	7333.33	8601.23	9603.01	10616.34
	80				16966	14195	16333	18036	19684	8323.94	6882.76	7975.89	8864.01	9745.84	10254.19	8987.18	10032.17	10810.90	11551.57
	90				19761	17792	19494	20752	21942	9786.47	8736.71	9648.74	10316.14	10935.86	11535.94	10725.03	11469.95	11992.30	12447.96

Supplementary Table S13. Planning unit, cost, and amount under different schemes and protection targets (screening probability 0.4).

Category	Target (%)	Total PU	Total Cost	Total Amount	Planning unit R0	Planning unit R1	Planning unit R2	Planning unit R3	Planning unit R4	Cost R0	Cost R1	Cost R2	Cost R3	Cost R4	Amount R0	Amount R1	Amount R2	Amount R3	Amount R4
Mangrove	10	12537	5980.06	7259.56	1032	487	638	921	1496	437.32	224.54	282.87	395.57	622.89	725.72	341.81	450.56	659.58	1062.54
	20				2145	756	946	1507	3114	981.74	362.07	445.85	689.01	1416.75	1451.90	532.01	667.99	1066.47	2154.12
	30				3381	1013	1919	3149	5385	1619.17	486.59	921.45	1510.43	2558.36	2177.85	705.05	1315.72	2109.21	3454.12
	40				4588	2177	3394	4949	6889	2218.56	1064.81	1651.59	2403.22	3327.59	2903.87	1466.90	2222.42	3132.47	4249.97
	50				5861	3596	5021	6368	7829	2846.39	1753.53	2448.09	3096.96	3774.11	3629.84	2328.51	3159.44	3937.05	4775.22
	60				7127	5038	6447	7672	9118	3424.23	2453.85	3122.74	3699.27	4364.93	4355.76	3169.84	3985.66	4686.84	5499.80
	70				8372	6470	7865	9076	10265	4005.31	3123.85	3777.19	4336.19	4897.56	5081.77	4007.94	4805.63	5485.06	6140.68
	80				9676	8182	9334	10323	11320	4614.65	3910.24	4453.87	4913.46	5380.14	5807.64	4993.63	5633.25	6177.76	6695.18
	90				11044	10060	11076	11646	12077	5256.76	4788.97	5265.76	5536.53	5747.88	6533.62	6040.74	6570.94	6852.76	7057.16
Salt marsh	10	44242	15362.41	25781.87	4112	1307	1469	1783	6474	987.75	358.92	398.21	463.52	1345.44	2578.13	859.31	961.48	1149.96	4016.72
	20				8494	1459	1875	8632	15971	2092.16	396.39	490.64	1873.99	4008.56	5156.49	954.59	1209.40	5294.53	9731.77
	30				12494	2014	9695	15127	20331	3338.31	529.91	2240.23	3813.83	5566.60	7734.76	1293.63	6040.92	9295.73	12470.65
	40				16615	7346	14689	19295	24515	4647.53	1687.62	3698.04	5222.66	7143.69	10312.82	4734.27	9098.37	11900.27	15049.77
	50				20814	14114	18964	22981	27742	5907.18	3517.34	5111.78	6566.36	8414.99	12891.01	8812.44	11740.32	14176.91	17016.33
	60				25249	18700	23016	27023	31465	7527.74	5048.09	6608.33	8155.46	9932.61	15469.12	11630.60	14242.12	16646.16	19209.35
	70				29578	23224	27539	31271	35822	9223.69	6699.07	8370.51	9844.78	11766.53	18047.33	14398.98	16993.37	19135.99	21689.46
	80				34164	28299	32642	36223	40254	11102.03	8682.00	10425.36	11944.41	13633.87	20625.56	17455.28	19944.69	21918.92	23949.26
	90				38961	34818	38766	41591	43444	13113.16	11350.68	13020.51	14184.88	15002.83	23203.66	21153.55	23210.02	24575.63	25435.48
Seagrass	10	16430	8230.14	10540.24	1351	75	91	430	2476	590.34	27.29	34.09	169.80	1034.35	1054.01	53.38	66.35	345.26	1894.88
	20				2886	189	1184	2643	5043	1283.56	74.79	503.46	1133.19	2261.00	2108.03	146.74	926.99	1998.42	3737.74
	30				4353	1307	2864	4617	6995	1978.40	564.60	1253.09	2071.61	3241.40	3162.14	1018.47	2154.28	3409.43	5072.39
	40				5900	2959	4698	6401	8562	2746.50	1307.57	2116.15	2953.23	4061.71	4216.09	2214.01	3442.24	4631.53	6080.44
	50				7522	5239	6671	8036	9663	3536.28	2375.18	3094.15	3803.20	4645.82	5270.15	3738.14	4748.46	5684.20	6748.28
	60				9059	6683	8298	9675	11323	4341.94	3104.49	3938.04	4654.25	5517.27	6324.24	4800.59	5887.87	6774.88	7810.27
	70				10676	8477	10078	11362	12727	5186.85	4032.09	4860.64	5532.60	6260.91	7378.26	6045.96	7057.45	7861.17	8653.30
	80				12366	10610	11950	13027	14316	6078.42	5143.36	5848.74	6415.85	7095.43	8432.34	7414.57	8211.56	8832.66	9528.36
	90				14315	12953	14121	14928	15811	7098.17	6372.14	6992.43	7414.67	7885.20	9486.24	8795.53	9429.79	9839.46	10265.20

Supplementary Table S14. Planning unit, cost, and amount under different schemes and protection targets (screening probability 0.5).

Category	Target	Total			Planning unit					Cost					Amount				
	(%)	PU	Cost	Amount	R0	R1	R2	R3	R4	R0	R1	R2	R3	R4	R0	R1	R2	R3	R4
Mangrove	10	8592	4090.84	5488.45	762	407	494	704	1087	330.18	190.46	224.71	305.51	456.64	548.71	289.31	353.32	510.24	788.66
	20				1544	723	885	1229	2108	701.80	345.40	414.53	555.76	932.99	1097.62	516.46	634.72	887.86	1503.55
	30				2374	900	1405	2208	3614	1122.18	430.76	673.61	1048.35	1697.59	1646.58	644.20	1013.77	1571.53	2514.37
	40				3226	1723	2459	3230	4543	1551.61	850.01	1201.32	1568.94	2181.57	2195.57	1223.79	1723.75	2227.94	3059.84
	50				4082	2650	3413	4323	5309	1980.36	1304.34	1669.47	2103.95	2563.42	2744.24	1840.26	2329.72	2903.79	3527.77
	60				4927	3340	4387	5226	6422	2356.77	1627.41	2121.73	2511.92	3068.02	3293.25	2288.87	2951.19	3476.96	4226.42
	70				5813	4408	5330	6207	7308	2781.17	2123.51	2552.46	2962.89	3469.11	3842.12	2963.07	3551.67	4096.94	4768.19
	80				6692	5461	6344	7273	8084	3184.43	2612.18	3022.10	3453.83	3833.60	4390.88	3636.73	4181.29	4745.35	5214.10
	90				7634	6826	7726	8174	8465	3621.43	3245.89	3666.32	3882.35	4023.36	4939.69	4472.05	5001.82	5261.24	5421.67
Salt marsh	10	30066	10471.11	19447.27	2930	1154	1557	2342	4770	642.43	312.43	374.88	520.28	979.76	1944.77	774.51	1010.65	1559.70	3160.57
	20				5950	1386	2405	6272	9916	1408.40	372.73	554.66	1319.26	2268.44	3889.45	927.89	1579.16	4073.92	6482.17
	30				8872	2208	6876	10137	13432	2206.87	525.44	1495.05	2426.77	3517.50	5834.27	1460.52	4474.48	6659.04	8909.48
	40				11785	7264	10337	12911	16178	3158.33	1652.56	2542.27	3402.37	4617.26	7778.91	4771.38	6820.22	8580.29	10768.08
	50				14744	10845	13398	15676	18268	4144.29	2733.06	3603.07	4441.63	5462.79	9723.77	7180.37	8911.64	10424.75	12127.05
	60				17576	13423	16149	18459	21343	5238.82	3620.16	4637.10	5540.96	6722.07	11668.36	8943.70	10760.73	12288.39	14155.03
	70				20559	16434	19170	21525	24454	6433.76	4753.59	5829.21	6798.07	8031.92	13613.09	10965.92	12766.01	14299.09	16168.87
	80				23562	19796	22538	24886	27706	7679.02	6083.54	7216.52	8220.68	9408.82	15557.82	13185.40	14955.58	16450.10	18140.36
	90				26728	24068	26553	28320	29609	9018.53	7876.45	8929.45	9687.85	10260.32	17502.60	15931.13	17456.41	18487.95	19207.59
Seagrass	10	12058	6029.30	8579.42	1061	54	88	525	1862	473.67	21.06	35.45	223.64	798.49	858.01	42.36	71.26	429.30	1472.20
	20				2251	436	1239	2190	3737	987.44	183.60	540.74	965.96	1681.61	1715.90	354.22	991.47	1707.95	2840.66
	30				3375	1135	2225	3537	5333	1520.72	495.36	982.15	1579.50	2450.22	2573.87	914.66	1745.42	2722.04	4058.72
	40				4557	2558	3780	4919	6260	2095.24	1138.59	1704.78	2258.90	2944.18	3431.98	1978.94	2861.13	3708.59	4713.17
	50				5719	4014	5158	6012	7333	2671.52	1827.48	2386.53	2826.92	3524.86	4289.72	3027.74	3879.83	4512.38	5478.68
	60				6923	5238	6309	7292	8506	3303.94	2425.74	2982.44	3499.63	4139.06	5147.72	3942.83	4746.49	5459.60	6323.00
	70				8083	6539	7612	8551	9540	3925.76	3096.05	3667.87	4163.19	4690.57	6005.67	4926.69	5699.99	6358.60	7052.91
	80				9304	8061	9025	9736	10548	4567.88	3901.04	4412.10	4795.94	5221.48	6863.62	6027.44	6701.22	7181.71	7706.45
	90				10612	9679	10436	11071	11708	5262.47	4766.64	5160.53	5501.00	5838.85	7721.48	7147.11	7629.60	8022.06	8390.00

Supplementary Table S15. Comprehensive spatial protection strategy statistics by province.

Category	Strategy		Liaoning	Tianjin	Hebei	Shandong	Jiangsu	Shanghai	Zhejiang	Fujian	Guangdong	Guangxi	Hainan	Total
Mangrove	Single	ECP	0	0	0	0	0	0	0	0	395.66	240.33	112.63	748.63
		ECU	0	0	0	0	0	0	1.55	48.63	262.13	105.01	129.83	547.15
		NCP	0	0	0	0	0	0	0	46.3	1452.4	849.7	285	2633.4
	Joint	ECP	0	0	0	0	0	0	0	7.9	147.51	135.22	45.12	335.75
		ECU	0	0	0	0	0	0	0	0.78	24.62	31.04	15.32	71.77
		NCP	0	0	0	0	0	0	0	0	28.76	0	0.81	29.56
Salt marsh	Single	ECP	498.58	0	47.37	129.15	519.1	388.24	224.82	67.93	0	0	0	1875.2
		ECU	245.42	20.2	23.64	209.68	239.6	225.71	1095.9	649.7	70.7	24.67	5.64	2810.86
		NCP	520.81	0	0	189.76	5708.45	2379.36	1804.98	509.57	0	0	0	11112.92
	Joint	ECP	13.87	0	0	102.46	0	0	0	10.16	0	0	0	126.49
		ECU	4.67	14.13	12.77	96.12	0	0	0.78	95.06	77.79	48.54	0.8	350.67
		NCP	0	0	0	538.17	0	0	0	36.62	0	0	0	574.79
Seagrass	Single	ECP	48.34	0	101.65	13.22	0	0	0	0	0	0	33.07	196.29
		ECU	42.98	4.73	4.02	50.87	2.19	0	0	3.94	3.96	4.78	9.71	127.18
		NCP	775.35	451.77	1012.81	680.9	0	0	0	0	0	0	196.4	3117.23
	Joint	ECP	0	0	8.08	19.98	0	0	0	0	14.4	0	41.95	84.4
		ECU	5.3	0	0	0	0	0	0	0	9.53	11.94	4.83	31.6
		NCP	0	0	0	220.83	0	0	0	0	21.57	0	34.74	277.13
Anthropogenic activity	First class		0	0	0	115.12	5.71	0	0	4.73	65.1	0	0	190.67
	Second class		944.19	0.67	298.44	4128.36	600.54	0	354.57	3771.87	2618.05	286.54	39.44	13042.65
	Third class		3840.22	127.68	1035.38	7191.08	909.61	104.18	6485.6	4953.97	8676.12	930.85	1945.64	36200.33

Supplementary Text S1. Validation and robustness assessment of PSDAs.

Because the identification of PSDAs forms the basis for subsequent conservation prioritization analyses, additional validation and robustness assessments were conducted to evaluate the reliability of the MaxEnt predictions. Specifically, we performed three complementary evaluations: (1) comparison with previously published habitat suitability studies, (2) assessment of model stability across repeated resampling runs, and (3) sensitivity analysis based on occurrence-record subsampling.

1.1 Comparison with previous studies

To independently evaluate the predicted PSDAs, we compared the spatial distribution patterns and dominant environmental drivers with results from previous habitat suitability assessments conducted at regional, national, and global scales.

For mangroves, the predicted PSDAs were concentrated in the southern coastal regions of China, particularly Guangdong, Guangxi, and Hainan Provinces, with suitability decreasing rapidly toward higher latitudes. These patterns are highly consistent with previous studies [3-5]. In addition to the broad geographic pattern, the locations of major suitability hotspots and the boundaries of highly suitable regions showed strong agreement with the published suitability maps. Temperature-related variables, precipitation, sea-surface conditions, and biotic interactions were identified as major drivers in both this study and previous investigations.

For salt marshes, the predicted PSDAs were mainly distributed along the central and northern coasts of China, particularly around the Yellow Sea and East China Sea regions. This pattern closely matched the national-scale distribution framework reported by Cui et al. (2025) [3], including the geographic transition between mangrove-dominated southern coasts and salt-marsh-dominated northern coasts. Similar environmental controls, including temperature and terrain-related variables, were identified across studies.

For seagrasses, the predicted PSDAs identified major suitability hotspots around Hainan Island and the Leizhou Peninsula, consistent with the habitat suitability patterns reported by Hu et al. (2021) [6]. Beyond the overall distribution pattern, the spatial locations and extents of highly suitable areas were also highly comparable. Salinity and offshore environmental conditions were identified as dominant predictors in both studies.

Overall, despite differences in occurrence datasets, environmental variables, modelling frameworks, and spatial scales, our predicted PSDAs exhibited strong agreement with previous independent studies in terms of both spatial distribution patterns and dominant environmental drivers, providing external support for the reliability of the model predictions.

1.2 Stability of MaxEnt predictions

To evaluate model stability, MaxEnt was run using ten replicate subsampling iterations for each ecosystem type. For each iteration, 75% of occurrence records were used for training and 25% for testing. Model performance was assessed using regularized training gain, unregularized training gain, test gain, training AUC, and test AUC (**Supplementary Table S16**).

Across all ten replicates, model performance remained highly stable. Mangrove models achieved mean training and test AUC values of approximately 0.926. Salt marsh models achieved mean training and test AUC values of approximately 0.834, while seagrass models achieved the highest predictive performance, with mean training and test AUC values exceeding 0.95. Variations among replicate runs were minimal for both gain and AUC metrics, indicating that model outputs were not sensitive to random partitioning of occurrence records. These results demonstrate strong predictive ability and high reproducibility of the MaxEnt models for all three CBCEs.

Supplementary Table S16. Model performance of ten repeated experiments on CBCEs.

Category	Number	Regularized training gain	Unregularized training gain	Test gain	Training AUC	Test AUC
Mangrove	1	1.6720	1.7281	1.7541	0.9265	0.9297
	2	1.6572	1.7109	1.7122	0.9258	0.9256
	3	1.6720	1.7284	1.7058	0.9262	0.9250
	4	1.6725	1.7289	1.7111	0.9267	0.9235
	5	1.6626	1.7152	1.7293	0.9258	0.9263
	6	1.6702	1.7227	1.7118	0.9263	0.9241
	7	1.6681	1.7240	1.6833	0.9261	0.9223
	8	1.6655	1.7205	1.7223	0.9260	0.9256
	9	1.6773	1.7303	1.7421	0.9264	0.9278
	10	1.6643	1.7228	1.7445	0.9261	0.9294
Salt marsh	1	0.8247	0.8765	0.8846	0.8345	0.8371
	2	0.8301	0.8807	0.8727	0.8345	0.8334
	3	0.8198	0.8720	0.8580	0.8339	0.8295
	4	0.8300	0.8813	0.8704	0.8354	0.8346
	5	0.8169	0.8661	0.8671	0.8330	0.8330
	6	0.8258	0.8756	0.8775	0.8342	0.8357
	7	0.8269	0.8785	0.8375	0.8346	0.8257
	8	0.8253	0.8762	0.8650	0.8349	0.8322
	9	0.8214	0.8722	0.8951	0.8341	0.8394
	10	0.8192	0.8704	0.8577	0.8338	0.8322
Seagrass	1	1.9408	2.1503	1.8811	0.9578	0.9444
	2	1.9299	2.1362	2.2053	0.9576	0.9616
	3	1.9042	2.1373	2.1081	0.9581	0.9573
	4	1.9688	2.1708	2.1009	0.9595	0.9561
	5	1.9884	2.2245	1.9762	0.9608	0.9490
	6	1.9099	2.1458	2.0508	0.9590	0.9526
	7	1.9445	2.1671	2.0941	0.9596	0.9557
	8	1.9362	2.1520	2.1473	0.9589	0.9587
	9	1.8831	2.1079	2.0536	0.9569	0.9547
	10	1.9168	2.1487	2.0512	0.9583	0.9530

1.3 Sensitivity analysis based on occurrence-record subsampling

Because species distribution models may be influenced by the quantity and spatial distribution of occurrence records, we further conducted a sensitivity analysis by randomly subsampling occurrence records. For each ecosystem type, 90%, 80%, and 70% of the original occurrence records were retained, corresponding to the removal of 10%, 20%, and 30% of occurrence records, respectively. MaxEnt models were then rerun using the reduced datasets and the resulting suitability was compared with those derived from the complete dataset (**Supplementary Fig. S20**).

Comparison showed that the predicted spatial patterns remained highly consistent across all subsampling scenarios. Major suitability hotspots, latitudinal gradients, and overall distribution boundaries were largely preserved even after removing 30% of occurrence records. Differences were mainly confined to small localized areas with intermediate suitability values.

To quantitatively evaluate agreement between the original and subsampled predictions, pixel-wise comparisons were conducted using coefficient of determination (R^2), root mean square error (RMSE), mean absolute error (MAE), and Schoener's D overlap metric (**Supplementary Fig. S21**). Across all ecosystem types, agreement remained high under all subsampling scenarios. When 10% of occurrence records were removed, Schoener's D values exceeded 0.92 for mangroves, salt marshes, and seagrasses, indicating extremely high similarity between predictions. Even when 30% of occurrence records were removed, Schoener's D values remained above 0.83 for all ecosystems. Correspondingly, R^2 values remained high and prediction errors (RMSE and MAE) remained low.

These results indicate that the predicted PSDAs are robust to moderate reductions in occurrence-record availability and that the major conclusions of this study are not driven by a limited subset of occurrence observations.

1.4 Conclusion

The reliability of the predicted PSDAs was supported by three independent lines of evidence. First, predicted spatial patterns and environmental drivers were consistent with previously published studies. Second, repeated MaxEnt runs produced stable gain and AUC values, indicating strong predictive performance and reproducibility. Third, sensitivity analyses demonstrated that substantial reductions in occurrence records resulted in only minor changes to predicted suitability patterns. Collectively, these results provide strong evidence that the PSDAs used in subsequent conservation prioritization analyses are robust and reliable.

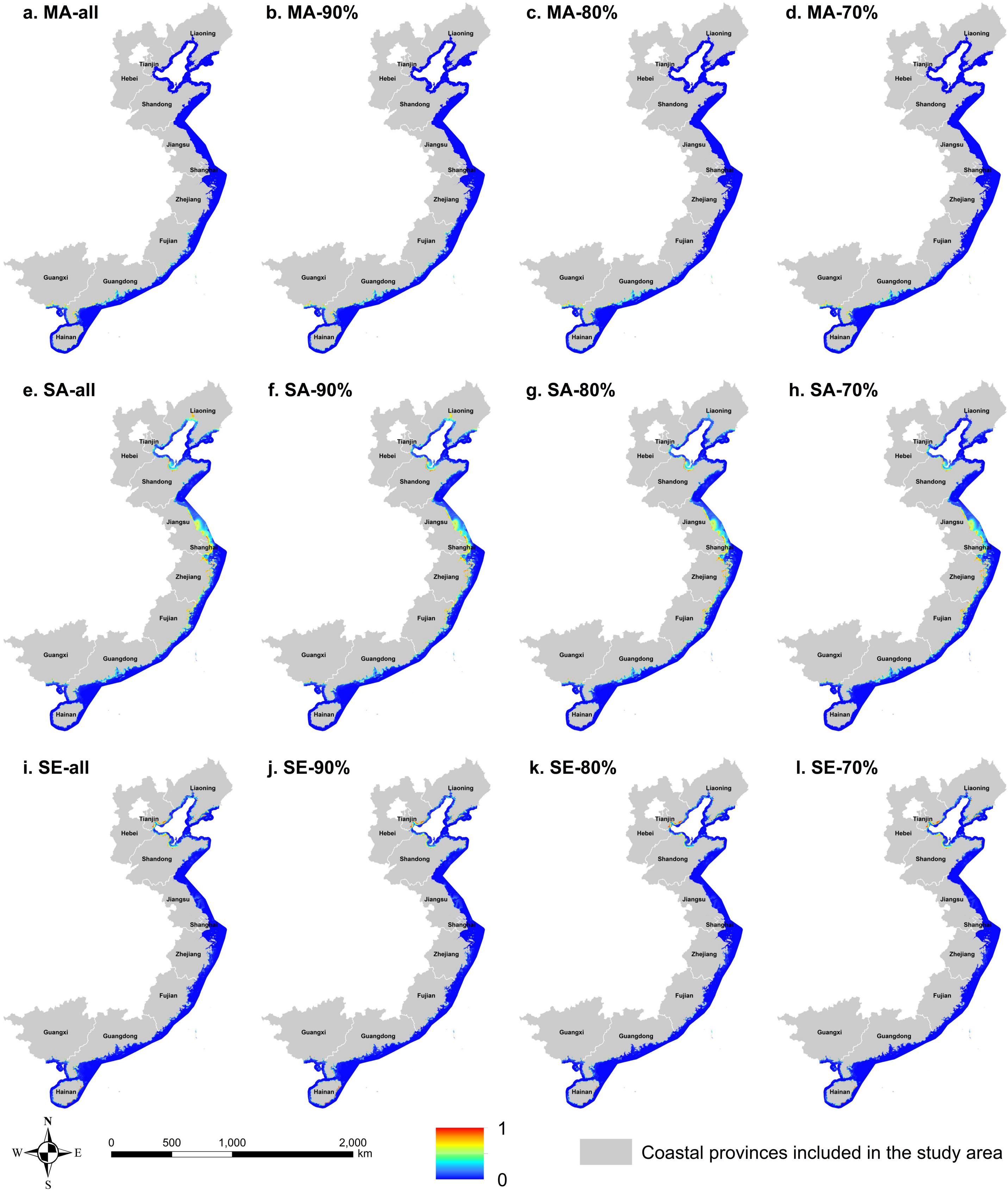

Supplementary Fig. S20 Comparison of suitability between the complete dataset and the deleted dataset.

Note: MA-mangrove; SA-salt marsh; SE-seagrass.

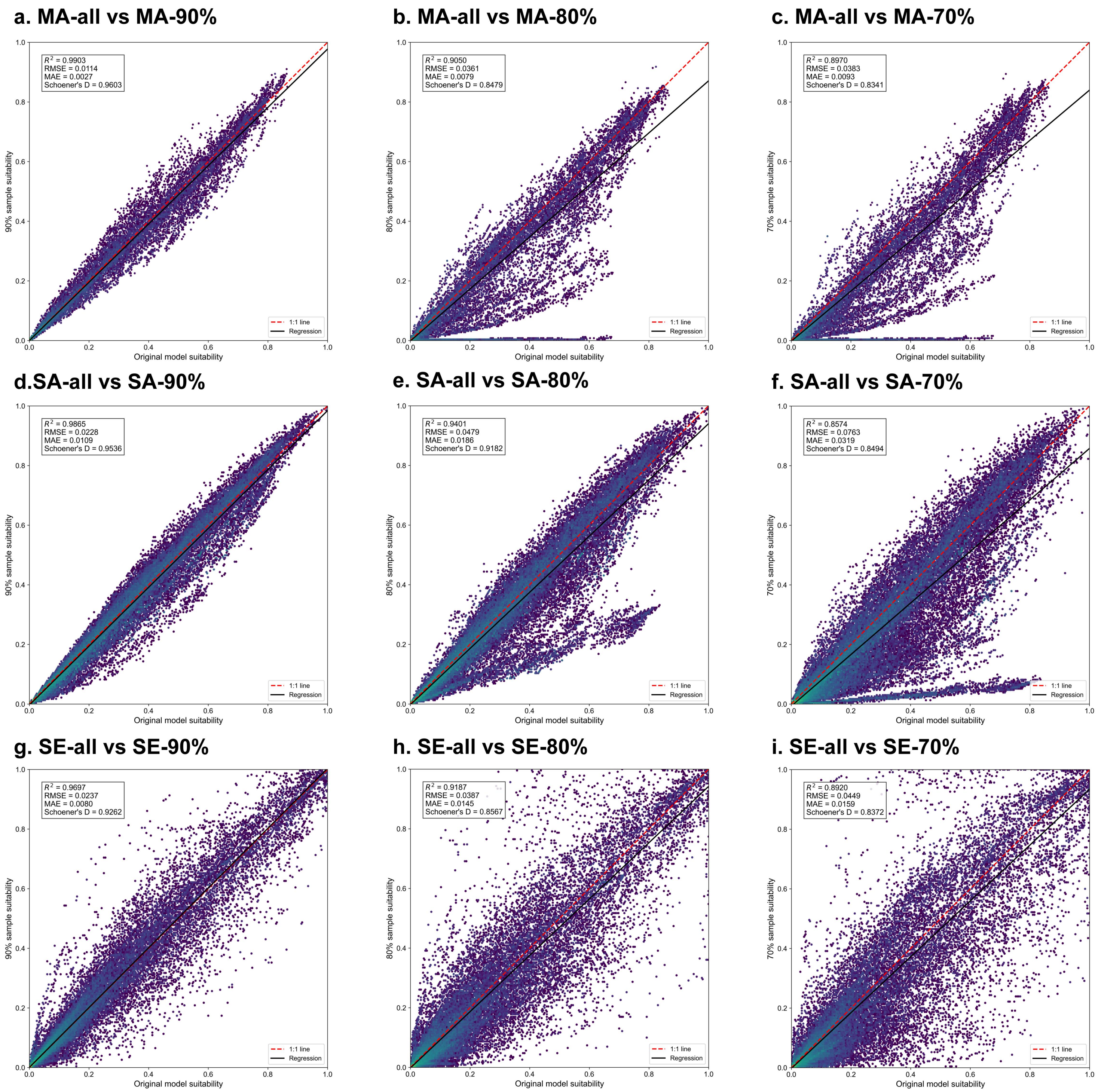

Supplementary Fig. S21 Comparison of agreement between the original estimations and the subsampled estimations.

Note: MA-mangrove; SA-salt marsh; SE-seagrass.

Supplementary Text 2. Economic feasibility assessment of establishing protected CBCE areas.

To provide a preliminary evaluation of the implementation feasibility of the proposed CBCE conservation network, we estimated the restoration costs and potential carbon sequestration values associated with conservation and restoration actions under a nature-based solutions framework. This assessment was not intended to constitute a comprehensive cost-benefit analysis. Rather, it serves as a first-order approximation of the economic resources required for implementation and the potential climate-mitigation benefits generated by protected and restored CBCEs.

The estimation of the construction cost of protected areas is based on median restoration costs of various CBCEs (mangrove \$8,961 ha^{-1}; salt marsh \$67,128 ha^{-1}; seagrass \$106,782 ha^{-1}) [7]. The total construction cost is obtained by multiplying the restoration cost per unit area by the area that needs to be repaired. The carbon sequestration resulting from restoration and conservation measures was estimated by multiplying the per-unit-area carbon accumulation rate (CAR) by the area of the restoration and conservation zone, based on monitoring data from CAR sampling points within CBCEs. CAR values were derived from field observations and published studies (primarily sourced from Fan & Li (2024) [8], Wang et al. (2021) [9], Taillardat et al. (2018) [10], Duarte et al. (2010) [11]). For zones lacking direct CAR monitoring points, values from adjacent sites of the same CBCE type were used as substitutes. The total carbon sequestration value was then calculated by multiplying the estimated carbon sequestration amount by the unit carbon price. The detailed calculation formula is as follows:

$$S_{SOC} = \sum_{i=1}^{n} Area_i \times CAR_i \times Value$$

where S_{SOC} denotes the annual total carbon sequestration value, n represents the total number of grids designated for CBCE restoration and protection, $Area_i$ indicates the area of the ith grid, CAR_i refers to the carbon accumulation rate of the ith grid, and *Value* represents the unit carbon price, for which this study adopts the Swedish carbon tax rate of 130 USD per ton of carbon. To account for the presence of degraded areas, the estimated value range is derived based on their degradation status.

It should be noted that the estimated carbon sequestration value represents only one component of the benefits generated by CBCE conservation. Other ecosystem services, including coastal protection, biodiversity conservation, fisheries enhancement, water purification, and human well-being benefits, were not monetized in this study. Therefore, the estimated values should be regarded as conservative lower-bound estimates of the potential societal benefits associated with the proposed conservation network.

Supplementary Table S17. The restoration cost, annual carbon sequestration and carbon sequestration value of CBCEs.

Province	Restoration cost (Unit: 10^8 USD)				Annual carbon sequestration (Unit: 10^6 t)				Annual carbon sequestration value (Unit: 10^8 USD)			
	Mangrove	Salt marsh	Seagrass	Total	Mangrove	Salt marsh	Seagrass	Total	Mangrove	Salt marsh	Seagrass	Total
Liaoning	0	34.96	82.79	117.75	0.00	1.07-1.33	0.24-0.26	1.31-1.59	0.00	1.39-1.73	0.31-0.33	1.71-2.06
Tianjin	0	0	48.24	48.24	0.00	0.00-0.03	0.13-0.13	0.13-0.16	0.00	0.00-0.04	0.17-0.17	0.17-0.21
Hebei	0	0	108.15	108.15	0.00	0.04-0.07	0.33-0.33	0.37-0.40	0.00	0.05-0.09	0.43-0.43	0.48-0.52
Shandong	0	48.86	96.29	145.15	0.00	0.81-1.07	0.61-0.64	1.42-1.71	0.00	1.05-1.39	0.79-0.83	1.84-2.22
Jiangsu	0	383.20	0	383.2	0.00	7.36-7.65	0.00	7.36-7.65	0.00	9.57-9.94	0.00	9.57-9.94
Shanghai	0	159.72	0	159.72	0.00	3.63-3.92	0.00	3.63-3.92	0.00	4.71-5.1	0.00	4.71-5.10
Zhejiang	0	121.16	0	121.16	0.00	2.52-3.89	0.00	2.52-3.89	0.00	3.28-5.05	0.00	3.28-5.05
Fujian	0.41	36.66	0	37.08	0.06-0.11	0.91-1.99	0.00	0.96-2.10	0.08-0.15	1.18-2.58	0.00	1.25-2.73
Guangdong	13.27	0	2.3	15.58	1.91-2.19	0.00-0.18	0.02-0.03	1.94-2.40	2.49-2.84	0.00-0.23	0.03-0.04	2.52-3.12
Guangxi	7.61	0	0	7.61	1.09-1.21	0.00-0.09	0.00-0.01	1.09-1.31	1.42-1.57	0.00-0.12	0.00-0.01	1.42-1.70
Hainan	2.56	0	24.68	27.24	0.42-0.55	0.00-0.01	0.18-0.19	0.60-0.75	0.54-0.72	0.00-0.01	0.24-0.25	0.78-0.98
Total	23.86	784.57	362.46	1170.89	3.48-4.06	16.33-20.21	1.52-1.60	21.33-25.87	4.52-5.28	21.24-26.27	1.97-2.08	27.73-33.63

Supplementary References

1. Halpern, B. S., et al. A Global Map of Human Impact on Marine Ecosystems. *Science* **319**, 948-952 (2008).

2. Halpern, B. S., et al. Recent pace of change in human impact on the world's ocean. *Scientific Reports* **9**, 11609 (2019).

3. Cui, L. et al. Comparing and quantifying the ecological niches of the saltmarsh grass Spartina alterniflora and major mangrove species in China. *Sci Rep* **15**, 23604 (2025).

4. Cui, L. et al. Global potential distribution of mangroves: Taking into account salt marsh interactions along latitudinal gradients. *J. Environ. Manage.* **351**, 119892 (2024).

5. Feng, B., et al. Identification of suitable mangrove distribution areas and estimation of carbon stocks for mangrove protection and restoration action plan in China. *J Mar Sci Eng* **12**(3), 445 (2024).

6. Hu, W. et al. Mapping the Seagrass Conservation and Restoration Priorities: Coupling Habitat Suitability and Anthropogenic Pressures. *Ecol. Indic.* **129**, 107960 (2021).

7. Bayraktarov, E. et al. The cost and feasibility of marine coastal restoration. *Ecol. Appl.* **26**, 1055-1074 (2016).

8. Fan, B., & Li, Y. China's conservation and restoration of coastal wetlands offset much of the reclamation-induced blue carbon losses. *Global Change Biology* **30**, e17039 (2024).

9. Wang F, et al. Global blue carbon accumulation in tidal wetlands increases with climate change. *National Science Review* **8**(9), nwaa296 (2021).

10. Taillardat P, et al. Mangrove blue carbon strategies for climate change mitigation are most effective at the national scale. *Biol. Lett.* **14**, 20180251 (2018).

11. Duarte, C. M. et al. Seagrass community metabolism: Assessing the carbon sink capacity of seagrass meadows. *Global Biogeochem. Cycles* **24**, GB4032 (2010).
